# Plasma Protein Characteristics of Long-Term Hemodialysis Survivors

**DOI:** 10.1371/journal.pone.0040232

**Published:** 2012-07-06

**Authors:** Yao-Ping Lin, Chih-Yu Yang, Chen-Chung Liao, Wen-Chung Yu, Chin-Wen Chi, Chao-Hsiung Lin

**Affiliations:** 1 Institute of Clinical Medicine, School of Medicine, National Yang-Ming University, Taipei, Taiwan; 2 Department of Medicine, Taipei Veterans General Hospital, Taipei, Taiwan; 3 Proteomics Research Center, National Yang-Ming University, Taipei, Taiwan; 4 Department of Medical Research and Education, Taipei Veterans General Hospital, Taipei, Taiwan; 5 Department of Life Sciences and Institute of Genomic Sciences, National Yang-Ming University, Taipei, Taiwan; 6 Department of Education and Research, Taipei City Hospital, Taipei, Taiwan; Ottawa Hospital Research Institute, Canada

## Abstract

Hemodialysis (HD) patients are under recurrent circulatory stress, and hemodialysis has a high mortality rate. The characteristics of plasma proteomes in patients surviving long-term HD remain obscure, as well as the potential biomarkers in predicting prognoses. This study reports the proteome analyses of patient plasma from non-diabetic long-term HD (LHD, dialysis vintage 14.9±4.1 years, n = 6) and the age/sex/uremic etiology-comparable short-term HD (SHD, dialysis vintage 5.3±2.9 years, n = 6) using 2-DE and mass spectrometry. In addition, a 4-year longitudinal follow-up of 60 non-diabetic HD patients was subsequently conducted to analyze the baseline plasma proteins by ELISA in predicting prognosis. Compared to the SHD, the LHD survivors had increased plasma vitamin D binding proteins (DBP) and decreased clusterin, apolipoprotein A-IV, haptoglobin, hemopexin, complement factors B and H, and altered isoforms of α1-antitrypsin and fibrinogen gamma. During the 45.7±15 months for follow-up of the 60 HD patient cases, 16 patients died. Kaplan-Meier analysis demonstrated that HD patients with the lowest tertile of the baseline plasma DBP level have a significantly higher mortality rate. Multivariate Cox regression analysis further indicated that DBP is an independent predictor of mortality. In summary, the altered plasma proteins in LHD implicated accelerated atherosclerosis, defective antioxidative activity, increased inflammation/infection, and organ dysfunction. Furthermore, lower baseline plasma DBP in HD patients is related to mortality. The results suggest that the proteomic approach could help discover the potential biomarker in HD prognoses.

## Introduction

A global incidence and prevalence of end-stage renal disease (ESRD) patients requiring maintenance dialysis has been on the rise [1]. Although hemodialysis (HD) is a major treatment modality to maintain their lives, these patients are under recurrent circulatory stress caused by intermittent dialysis sessions [2]. In addition, ESRD patients still have substantially reduced life expectancy, mostly because of cardiovascular disease [3].

For ease of obtaining research specimens, plasma has the advantage over cells or tissue because it can be collected in a relatively non-invasive manner. Moreover, all the protein components are readily accessible in a single compartment without requiring additional extraction procedures. Advances in proteomic techniques have facilitated the investigation of global changes in human plasma proteomes. Because of their pivotal clinical implications, intensive efforts and large-scale collaborative studies have been devoted to compiling human plasma proteomes for further integrated studies [4]. Applications of plasma proteome analysis are emerging in studies on cancer [5] and cardiovascular [6] and infectious diseases [7]. In HD patients, Chu et al. focused on the identification of toxins that accumulate in the uremic sera [8]. Langlois et al. aimed to clarify differences in the serum proteome between HD patients and healthy participants [9]. Weissinger et al. studied the effects of oral vitamin C supplementation on plasma polypeptide profiles in HD patients by CE-ESI-TOF MS [10]. Profiling plasma proteome in patients surviving long-term HD (LHD) and identifying potential biomarkers for prognoses is worthy of investigation.

The present study hypothesizes that plasma proteomes of LHD survivors might be different from those who received short-term HD (SHD). In addition, certain protective or beneficial markers might be evolved to reflect the dialysis-related circulatory stress. To address this issue, we conducted a 2-DE-based comparison of the plasma proteomes of ESRD patients receiving different maintenance HD vintage. Subsequently, the differentially expressed proteins were identified by MALDI-TOF mass spectrometry. Specifically, the plasma level of a potential protein marker, vitamin D binding protein (DBP), was measured using ELISA in another 60 HD patients to characterize the clinical significance of DBP during a 4-year prospective follow-up. Our study on plasma protein profiles in HD patients with different dialysis vintage might act as a reference for mining potential biomarkers for prognoses.

## Materials and Methods

### Human Subjects

Each patient gave written informed consent, and the study protocol was approved by the Institutional Review Board of Taipei Veterans General Hospital. Non-diabetic ESRD patients under maintenance HD were enrolled in the present study if they met the following criteria: (1) had undergone maintenance HD for more than 6 months, (2) had no recent cerebrovascular or cardiovascular events, and (3) had no symptoms or signs of concurrent infection, active autoimmune/collagen vascular disease, liver dysfunction, or malignancy. For a comparison of plasma proteomes, we enrolled long-term non-diabetic HD (dialysis vintage 14.9±4.1 years, n = 6) patients and the age/sex/uremic etiology comparable short-term HD (dialysis vintage 5.3±2.9 years, n = 6). To determine the clinical significance of our identified proteins, another 60 prevalent non-diabetic HD patients were further enrolled for prospective follow-up.

**Table 1:** Demographics and biochemistry of the studied HD population.

	Screening subjects		*P*
	Short-term hemodialysis n = 6	Long-term hemodialysis n = 6	Short vs. Long-term hemodialysis
Age, years	68.4±15.2	60.0±17.1	0.250
Gender, female/male	4/2	4/2	
Dialysis vintage, years	5.3±2.9	14.9±4.1	0.001
Etiology			
Chronic glomerulonephritis	3	3	
Chronci interstitial nephritis	3	3	
Body weight, kg	55.1±7.8	52.6±5.8	0.540
Height, cm	156.7±10.0	157.0±7.2	0.948
Body mass index, kg/m^2^	21.6±2.9	20.3±1.8	0.359
Systolic blood pressure, mmHg	145.3±11.7	141.0±16.1	0.609
Diastolic blood pressure, mmHg	80.3±6.2	77.1±4.9	0.347
Blood chemistry			
Leukocyte count, ×10^3^/µL	6.303±1.725	5.688±1.258	0.496
Hemoglobin, g/dL	11.1±2.1	10.1±1.9	0.425
Hematocrit, %	34.9±7.5	31.8±5.6	0.432
Albumin, g/dL	4.1±0.2	4.3±0.3	0.351
Blood urea nitrogen, mg/dL	84.2±10.3	92.9±6.8	0.113
Creatinine, mg/dL	10.8±1.9	12.1±1.3	0.202
Sodium, meq/dL	141.5±1.8	142.0±2.2	0.674
Potassium, meq/dL	5.1±0.6	5.0±0.8	0.875
Fasting blood sugar, mg/dL	99.7±6.6	98.1±16.3	0.827
Cholesterol, mg/dL	171.1±40.5	195.1±43.7	0.347
HDL-C, mg/dL	55.5±31.6	51.7±14.7	0.793
LDL-C, mg/dL	129.7±54.6	129.0±36.6	0.981
ApoB, mg/dL	106.3±41.9	104.3±24.3	0.922
Lp(a), mg/dL	8.1±4.3	12.3±8.8	0.594
Triglyceride, mg/dL	149.6±47.5	147.4±75.3	0.955
Uric acid, mg/dL	8.9±1.2	9.4±1.4	0.505
Alanine aminotransferase, U/L	19.1±5.7	23.3±5.0	0.200
γ-glutamyl transferase, U/L	31.1±29.5	14.6±5.2	0.207
Alkaline phosphatase, U/L	88.5±28.5	127.8±50.1	0.125
Calcium, mg/dL	9.5±0.9	9.6±0.8	0.675
Phosphate, mg/dL	5.3±0.7	5.6±1.4	0.643
Intact-parathyroid hormone, pg/mL	269.7±295.3	315.2±264.4	0.553
25(OH) Vitamin D, ng/mL	16.6±5.5	16.9±4.6	0.916
Kt/V	1.7±0.2	1.7±0.1	0.982

HD was prescribed as 4-h dialysis sessions 3× weekly using 1.8-m^2^ surface area dialyzers with bicarbonate-based dialysate (HCO_3_^−^ 39 mEq/L, Na^+^140 mEq/L, K^+^2.0 mEq/L, Ca^2+^3 mEq/L and Mg^2+^1 mEq/L). All patients received subcutaneous recombinant erythropoietin at a mean dosage of 20 000 units monthly to maintain a hematocrit level of 30%, according to the guidelines set by the National Health Insurance Bureau of Taiwan. Vitamin D supplements and statin were prescribed according to the Kidney Disease Outcome Quality Initiative (KDOQI) clinical practice guidelines. The efficacy (adequacy) of HD was indexed by the clearance index Kt/V [11]. Adherence to diet was assessed by serum K^+^, PO4^3−^, and albumin [12].

### Preparation of Plasma Samples

All plasma samples were collected on a mid-week non-dialysis day to avoid possible acute generation or clearance of proteins by the dialysis procedure. The patients’ blood was drawn in EDTA-tubes (BD, Franklin Lakes, NJ, USA) and immediately centrifuged at 3000 rpm at 4°C for 15 min. The plasma were thereafter divided into aliquots and stored at −80°C. Samples were centrifuged after thawing without visible fibrinogen precipitation, and subjected to subsequent 2D and ELISA analyses.

### Two-dimensional Gel Electrophoresis (2-DE)

The 2-DE analyses of the collected plasma were performed individually. Before electrophoresis, all plasma samples were carefully inspected to ensure that no hemolysis was present, which might have affected the analysis results. Protein concentration was determined using the PlusOne 2-D Quant Kit (GE Healthcare, Piscataway, NJ, USA), according to the manufacturer’s manual. To detect the less abundant proteins present in the plasma, Qproteome Albumin/IgG Depletion Kits (Qiagen, Hilden, Germany) were used as a prefractionation step to remove abundant human albumin and immunoglobulins with similar efficiency among samples. The 2-DE was performed using an IPGphor IEF system (GE Healthcare, Piscataway, NJ, USA) and a PROTEAN IIxi Cell electrophoresis unit (Bio-Rad, Hercules, CA, USA). Plasma protein was first dissolved in a lysis buffer containing 7 M urea, 2 M thiourea, 4% 3-[(3-cholamidopropyl)dimethylammonio]-1-propanesulfonate, and 2 mM triphenylphosphine. Thereafter, approximately 120 µg of protein was loaded into 18 cm, linear pH 3–10 Immobiline DryStrips (GE Health Care, Uppsala, Sweden) for in-gel rehydration. Isoelectric focusing was conducted using the following program: 0 V×3.5 h; 50 V×3.5 h; 200 V×1 h; 500 V×1 h; 1000 V×1 h. The gradient was raised from 1000 V to 8000 V within 30 min, and the focusing was terminated after 70 000 Vh. After isoelectric focusing, the IPG strips were equilibrated in an equilibrium buffer (50 mM Tris-HCl (pH 8.8), 6 M urea, 2% w/v SDS, 30% v/v glycerol, and 1% w/v dithiothreitol) for 15 min, and then soaked in the same buffer containing 2.5% w/v iodoacetamide for another 15 min. The strips were then placed on a 12% SDS-PAGE gel and immobilized with agarose sealing solution (0.5% w/v agarose, 25 mM Tris, 192 mM glycine, 0.1% w/v SDS, and a trace of bromophenol blue). SDS-PAGE was performed with this strip, and the resulting gel was subsequently fixed in the buffer (40% v/v methanol, 10% v/v acetic acid) for 30 min. The fixed gel was then incubated in 30% methanol for 15 min, washed by Milli-Q water, and placed in 0.05% w/v sodium thiosulfate for 2 min. After washing with Milli-Q water, gels were incubated in 0.2% silver nitrate for 25 min. The gels were then washed quickly and developed with 3% w/v sodium carbonate, 0.001% w/v sodium thiosulfate, and 0.02% formaldehyde, until the desired spot profiles were obtained. Development was stopped by 1.4% EDTA. Gel images were obtained using a flatbed scanner and were quantitatively analyzed by PDQuest v7.2 software (Bio-Rad, Hercules, CA, USA) for the MWs, pIs, and relative intensities of all protein spots.

**Figure 1:**
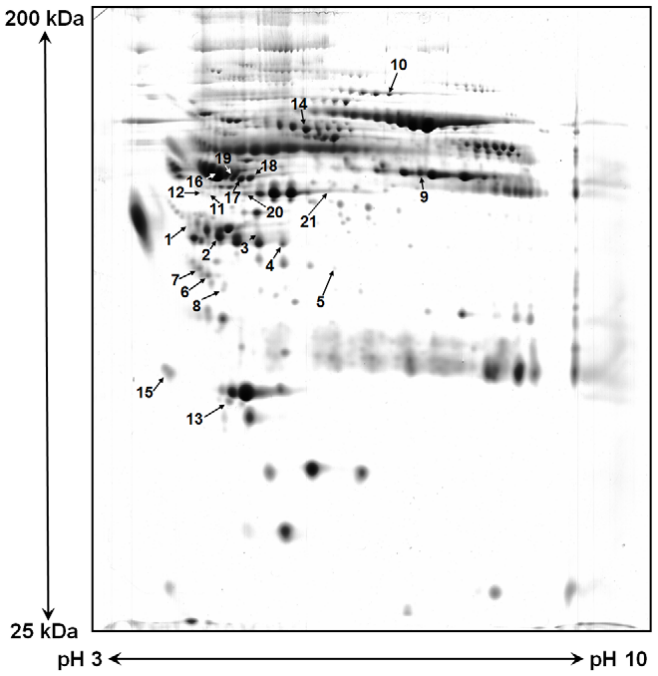
A representative 2D gel of plasma proteins from a long term HD patient. Plasma proteins were pre-treated with an albumin depletion kit and then separated according to their *p*I (IEF) and mass (SDS-PAGE). An average of 598 spots were detected per gel and further analyzed by MS or 2D database comparisons. The identified protein spots with differential expression related to HD duration were numbered and summarized in Table 2.

### In-gel Digestion

Selected gel pieces were manually excised and placed into 1.5 mL Eppendorf tubes. For destaining, the gel pieces were washed 3× with 100 µL 25 mM NH_4_HCO_3_/50% (v/v) acetonitrile (ACN) for 15 min. The solution was removed and 100 µL of 100% ACN was added to dehydrate the gel pieces. Thereafter, 1.6 µL of 20 ng/µL sequencing grade trypsin (Promega, Madison, WI, USA) in 25 mM NH_4_HCO_3_ was added to the dried gel pellets, and the reaction was incubated at 4°C for 40 min. An extra 2 µL of 25 mM NH_4_HCO_3_ was added, and the tube was kept at 55°C for 1 h. Finally, the gel pieces were sonicated with 7 µL 1% formic acid for 15 min to release the peptides into the solution, and these final products were ready for protein identification by using the following mass-spectrometric analysis.

**Table 2:** Lists of differentially expressed proteins in the plasma of long-term HD compared to the short-term HD patients.

Spot No.	Protein names	Theoretical Mr (kDa)/pI	Observed Mr (kDa)/pI	Identification method^a^	Sequence coverage	MASCOT score	Fold changes
1	Haptoglobin	38/6.27	44/4.74	sequencing	37	617	↓ 2.6±0.8
2	Haptoglobin	38/6.27	43/5.04	pmf	22	79	↓ 4.3±1.4
3	Haptoglobin	38/6.27	42/5.32	sequencing	24	337	↓ 3.9±1.5
4	Haptoglobin	38/6.27	42/5.50	sequencing	27	349	↓ 2.0±0.5
5	Haptoglobin	38/6.27	38/5.90	pmf	17	47	↓ 2.9±0.4
6	Clusterin	52/6.06	37/4.92	sequencing	6	97	↓ 1.9±0.4
7	Clusterin	52/6.06	38/4.84	sequencing	6	86	↓ 1.8±0.3
8	Clusterin	52/6.06	35/5.08	sequencing	4	59	↓ 3.1±0.1
9	Complement factor H	51/6.77	60/6.32	sequencing	10	180	↓ 2.0±0.4
10	Complement factor B	85/6.55	102/6.17	sequencing	12	68	↓ 2.5±1.0
11	α1-antitrypsin	44/5.37	51/4.93	pmf	21	87	↓ 2.8±0.4
12	α1-antitrypsin	44/5.37	51/4.86	pmf	28	96	↓ 2.3±0.8
13	Apolipoprotein A-IV	28/5.39	22/5.11	pmf	41	130	↓ 3.2±0.8
14	Hemopexin precursor	51/6.55	80/5.69	pmf	18	69	↓ 2.2±0.1
15	α1-antitrypsin	44/5.37	25/4.56	pmf	17	56	↑ 3.6±1.4
16	α1-antitrypsin	44/5.37	59/5.02	pmf	35	89	↑ 2.6±0.8
17	Vitamin D binding protein	53/5.40	57/5.18	sequencing	19	313	↑ 2.8±1.0
18	Vitamin D binding protein	53/5.33	58/5.27	pmf	30	133	↑ 5.2±0.7
19	Vitamin D binding protein	53/5.33	57/5.25	pmf	47	140	↑ 2.3±0.5
20	Fibrinogen gamma chain	49/5.61	50/5.61	pmf	21	108	↑ 1.9±0.2
21	Fibrinogen gamma chain	52/5.37	50/5.61	pmf	36	134	↓ 2.1±0.4

a Protein identification was done by the peptide mass fingerprinting (pmf) using MALDI-TOF MS, or by sequencing amino acids using Q-TOF tandem MS, as shown in the supplementary materials including Figure S1 and Data S1.

### Mass Spectrometry

Protein identification by peptide mass fingerprinting was performed on an Ultraflex MALDI-TOF MS (Bruker Daltonics, Bremen, Germany). The tryptic peptides were mixed 1∶1 with 2 mg/mL of α-cyano-4-hydroxycinnamic acid (CHCA) in a water/ACN/TFA (50∶50:0.1 v/v) solution and deposited on an AnchorChip 600/384 (Brucker). A peptide mixture of Angiotensin II (MH^+^1046.5418), Angiotensin I (MH^+^1296.6848), Substance P (MH^+^1347.7354), Bombesin (MH^+^1619.8223), ACTH (1–17, MH^+^2093.0862), and ACTH (18–39, MH^+^2465.1983) was used as the external standard for molecular weight calibration. Peptide samples were analyzed over a detection range from 800 to 3500 m/z. The batch process for peak list analysis was conducted using flexAnalysis 2.0 (Bruker Daltonics, Bremen, Germany). During the batch process, the signal detection algorithm SNAP (Bruker Daltonics, Bremen, Germany) was used for peak detection, and the criterion for the signal-to-noise ratio was set to 4. Each recorded mass spectrum was further analyzed by the MASCOT program (http://www.matrixscience.com) by searching the NCBInr database with the following settings: digested enzyme, trypsin; missed cleavage site, one; variable modification: carbamidomethylation (Cysteine) and oxidation (Methionine, Histidine and Tryptophan); peptide tolerance, less than 150 ppm and mass values, MH^+^, and monoisotopic. Only proteins with MOWSE scores above the significance level were considered identified.

For those spots that could not be identified by MALDI-TOF, further sequencing of peptide amino acids was performed using LC-MS/MS (QTOF-2 from Micromass, Manchester, UK). Briefly, tryptic peptides were separated on a reversed-phase C18 capillary column then delivered into to the electrospray source of mass spectrometer. The MS was operated in positive ion mode with source temperature at 80°C and cone voltage set to 45 V. A voltage of 3.2 kV was applied to the source capillary. Resulting MS/MS spectra were recorded in the data-dependent acquisition mode whereby the four most abundant doubly or triply-charged ions were selected for collision-induced dissociation of which collision energies were set to 10 and 30 V for MS and MS/MS scans respectively. Mass spectra were processed using the MassLynx 4.0 software (Micromass) and the protein identities were analyzed using the MS/MS peak lists generated from MassLynx. Protein identification was carried out by interpreting MS/MS data based on NCBInr gene database with the following parameters: taxonomy: Homo sapiens; digested enzyme: trypsin; missed cleavage site: one; variable modification: carbamidomethylation (cysteine) and oxidation (methionine); peptide mass tolerance: 1 Da; MS/MS tolerance: 0.6 Da; data format: micromass pkl file; monoisotopic mass and peptide charge of 2^+^/3^+^ were chosen.

### Enzyme Linked Immunosorbent Assay (ELISA)

The blood concentrations of human DBP and clusterin were measured using ELISA kits (Immun Diagnostik, Bensheim, Germany; BioVendor Laboratory Medicine Inc., Brno, Czech Republic), with a sensitivity of 0–60 ng/mL and 0–640 U, respectively. All reagents, samples, and working standards were prepared according to the manufacturer’s instruction. Each measurement was performed in duplicate. In brief, a 100 µL assay dilution buffer was added to each well prior to the addition of the standard or sample in the capture antibody-coating microplate. Thereafter, 100 µL of samples, standard or control, were incubated at room temperature for 1 h with horizontal shaking. Unbound residue was washed off by a 350 µL wash buffer for 4 times; subsequently, 100 µL of HRP-conjugated antibody was added, and the mixture was incubated for 1 h. After 4 more washes with the buffer, a 100 µL premixed enhanced luminal and hydrogen peroxide substrate was added and incubated for 10 min to 20 min at ambient temperature. The relative light units of each sample reaction were then read within 30 min by an automated reader. Sample concentrations were calculated using the machine program by comparing with a best-fit 4-parameter logistic standard curve.

### Statistics

SPSS 15.0 was used for statistical analysis. Continuous data were expressed as mean ± standard deviation (SD). Comparisons between different groups were analyzed by the Student’s *t* test for continuous variables or χ2 test for categorical variables. Kaplan-Meier analysis was used to compare the mortality rate related to the tertile of the DBP protein level. The Cox proportional hazards model was used to investigate significant factors for determining mortality. Factors related to mortality under univariate Cox regression analysis with *p*-values less than 0.10 were entered for further multivariate Cox regression analysis. A *p*-value of less than 0.05 was considered statistically significant.

**Figure 2:**
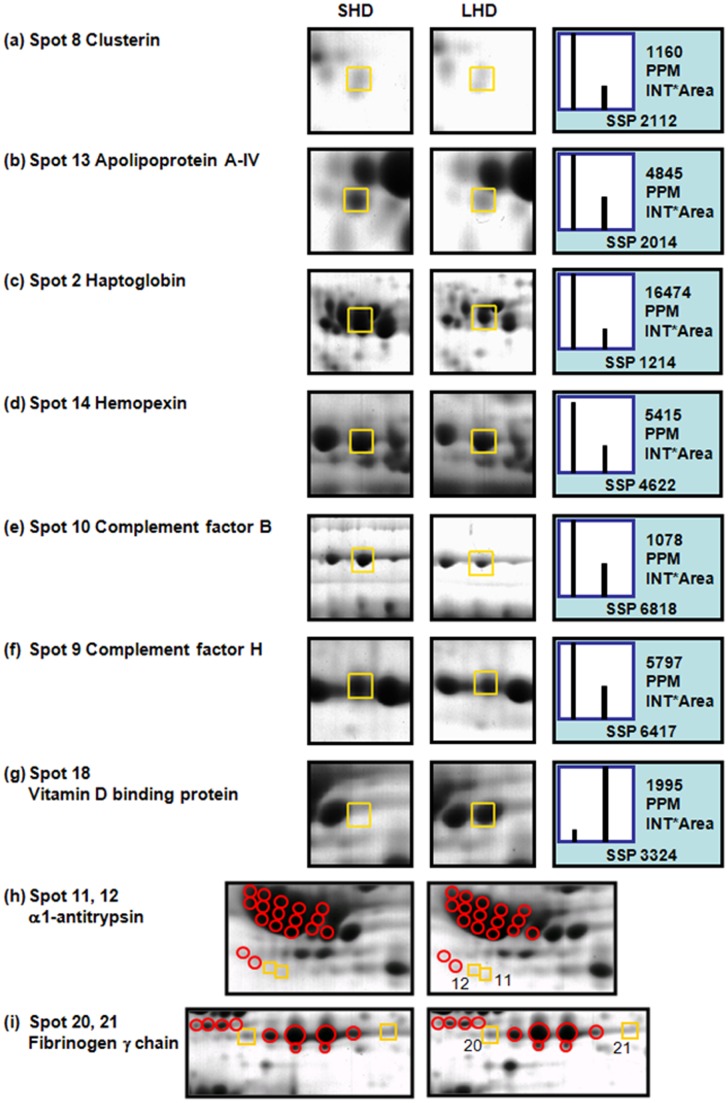
Representative gel sections of protein alterations related to HD duration. Each bar represents the intensity of the squared spot quantitatively analyzed by PDQuest software. Circles were used to indicate the α1-antitrypsin and fibrinogen γ chain respectively in (h) and (i) according to SWISS-2DPAGE (http://tw.expasy.org/ch2d/).

## Results

The demographic and biochemical data of the participating HD patients are summarized in Table 1. Except for the dialysis vintage, the backgrounds of the SHD and LHD patients were similar for age, sex, and anthropometrical indices such as height, weight and body mass index, blood pressure, lipid profile, calcium, phosphate, and the dialysis adequacy index Kt/V.

**Figure 3:**
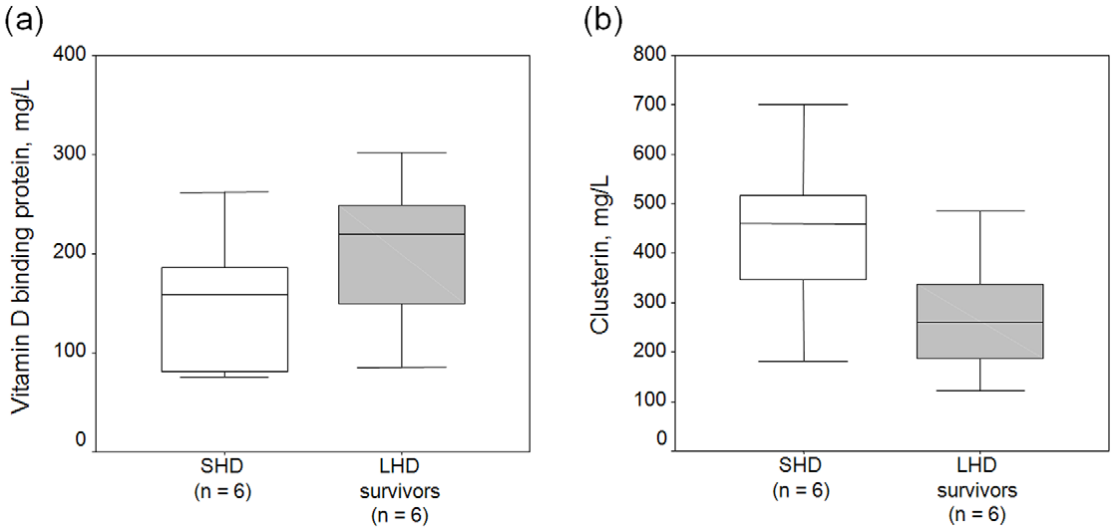
Box plots of plasma vitamin D binding protein and clusterin levels in HD patients. ELISA validation of vitamin D binding protein and clusterin concentration for patients’ plasma originally analyzed by 2-DE. Long-term HD survivors have (a) higher vitamin D binding protein (204.5±63.9 mg/L vs. 149.0±71.6 mg/L, p = 0.036) and (b) lower clusterin (282.3±105.8 mg/L vs. 438.5±174.2 mg/L, p = 0.011) than the short-term HD patients, which are concordant with 2-DE expression pattern.

Patient plasma samples were analyzed by 2-DE over a pH ranged between 3 and 10 to obtain optimal resolution and protein separation. An average of 598±116 spots in each silver-stained gel was visualized and subsequently quantified using PDQuest software (Bio-Rad). After deliberate inspection of each gel image and comparative analysis, a consistent trend of 2 increased spots (≥1.5 folds) and 19 decreased spots (≤1.5 folds) were observed in the LHD patients, compared to those of the other 6 age- and sex-comparable SHD counterparts. The locations of the differential spots are indicated with numbers in a representative gel of LHD patient plasma (Fig. 1).

**Table 3:** Comparison between the surviving and deceased HD patients in a four-year follow-up.

	Survivorsn = 44*	Deceasedn = 16*	*P*
ELISA - Vitamin D binding protein,mg/L	214.7±60.8	165.6±59.6	0.007
Age, years	58.2±14.5	67.8±12.5	0.021
Gender, male %	35	44	0.530
Dialysis vintage, years	7.9±5.5	5.9±4.2	0.158
Etiology			0.981
Chronic glomerulonephritis	32	11	
Chronci interstitial nephritis	5	2	
Hypertensive nephrosclerosis	4	2	
Lupus nephritis	3	1	
Body weight, Kg	53.4±7.9	56.8±8.8	0.273
Height, cm	157.2±7.9	160.6±8.7	0.157
Body mass index, kg/m^2^	21.2±2.8	21.9±2.9	0.365
Systolic blood pressure, mmHg	141.8±14.4	145.4±16.7	0.422
Diastolic blood pressure, mmHg	79.1±6.9	79.5±9.2	0.850
Laboratory data			
Leukocyte count, ×10^3^/µL	6.231±1.345	5.696±1.180	0.163
Hemoglobin, g/dL	11.8±1.9	11.2±1.7	0.517
Hematocrit, %	31.8±3.2	31.8±5.9	0.994
Albumin, g/dL	4.2±0.2	4.1±0.3	0.113
Blood urea nitrogen, mg/dL	90.8±15.4	92.7±17.2	0.683
Creatinine, mg/dL	10.6±1.8	10.3±1.6	0.843
Sodium, meq/dL	140.6±2.4	140.6±2.2	0.983
Potassium, meq/dL	5.1±0.7	5.3±0.7	0.303
Fasting blood sugar, mg/dL	103.6±18.8	110.8±17.6	0.189
Cholesterol, mg/dL	177.3±36.4	173.5±35.6	0.717
HDL-C, mg/dL	54.8±20.9	55.4±16.1	0.921
LDL-C, mg/dL	125.1±45.5	109.5±54.8	0.309
ApoB, mg/dL	102.7±30.7	90.4±38.1	0.243
Lp(a), mg/dL	8.1±8.7	10.2±9.2	0.457
Triglyceride, mg/dL,	154.7±85.5	177.3±157	0.523
Uric acid, mg/dL	8.7±1.4	8.3±1.3	0.255
Alanine aminotransferase, U/L	19.5±14.4	23.2±11.1	0.360
γ-glutamyl transferase, U/L	26.6±24.2	26.5±22.3	0.992
Alkaline phosphatase, U/L	112.4±55.1	139.1±71.9	0.128
Calcium, mg/dL	9.4±0.9	9.2±0.8	0.153
Phosphate, mg/dL	5.5±1.3	5.8±1.3	0.458
Calcium × Phosphate product, mg^2^/dL^2^	53.2±12.2	53.6±14.5	0.932
Intact-parathyroid hormone, pg/mL	268.5±294.1	360.3±231.3	0.285
25(OH) Vitamin D, ng/mL	16.9±7.7	19.0±7.8	0.388
Kt/V	1.69±0.17	1.63±0.22	0.397

* Values are expressed as mean ± SD or percent unless otherwise listed.

Protein spots with greater than 1.5-fold intensity change upon HD duration were excised, trypsin-digested, and determined by mass spectrometry. Table 2 shows a summary of the protein identification results. The sequence coverage of the identified proteins ranges from 4% to 47%, depending on the protein size and quantity. The enlarged gel sections displayed in Fig. 2 show the expression patterns of plasma proteins, including clusterin, apolipoprotein A-IV, haptoglobin, hemopexin, complement factor B, complement factor H, DBP, α1-antitrypsin, and fibrinogen γ, in the representative SHD and LHD patients. In each bar representation, the intensities of the squared spots were quantitatively analyzed using PDQuest software (Fig. 2). In the plasma of LHD patients, we observed the presence of increased DBP protein, as well as downregulation of a number of proteins, including clusterin, apolipoprotein A-IV, haptoglobin, hemopexin, complement factor B, and complement factor H, compared with those of the SHD counterparts. In addition, the isoform profiles of α1-antitrypsin and fibrinogen γ were altered, as shown in Fig. 2.

**Figure 4:**
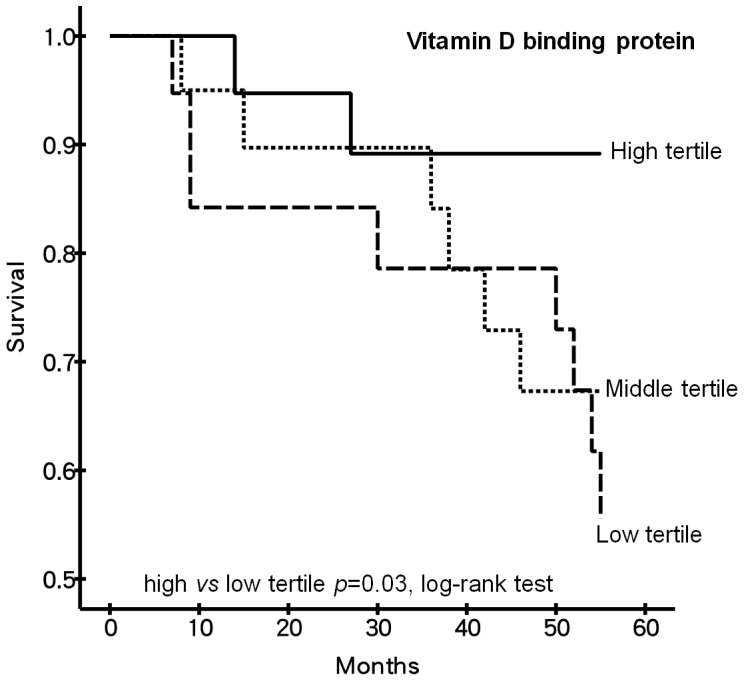
Kaplan-Meier analysis of plasma DBP level in HD patients. HD patients with the lowest plasma vitamin D binding protein level were at the highest risk for mortality than those with the top tertile plasma levels (p = 0.03; log-rank test).

Among the identified proteins, we further used ELISA to validate the plasma concentrations of DBP and clusterin in the same patients’ plasma analyzed by 2-DE. Concordant with the 2-DE expression patterns, the LHD patients (n = 6, Group II in Table 1) have significantly higher DBP levels (204.5±63.9 mg/L vs 149.0±71.6 mg/L, *p* = 0.036) and lower clusterin (282.3±105.8 mg/L vs 438.5±174.2 mg/L, *p* = 0.011) than SHD patients (n = 6, Group I in Table 1) (Fig. 3).

To explore the clinical implication of plasma levels of DBP in HD patients, we followed up another 60 prevalent HD patient s based on their initial plasma DBP detected by ELISA. During the follow-up of 45.7±15.9 months, 16 patients died (10 acute coronary events and 6 heart failures). The baseline plasma ELISA data of the dead patients (n = 16) disclosed their lower DBP compared to the surviving patients (n = 44) (165.6±59.6 vs 214.7±60.8 mg/L, *p* = 0.007). No significant differences existed for HD duration, age, sex, anthropometrical indices, blood pressure, lipid profile, calcium, phosphate, and Kt/V between the dead and living patients (Table 3).

Kaplan-Meier analysis demonstrated that HD patients with the lowest tertile of baseline plasma DBP level have a significantly higher mortality rate compared to patients with the top tertile level (*p* = 0.030; log-rank test) (Fig 4). In addition, initial univariate Cox regression analysis of factors significantly associated with mortality (*p*<0.1) disclosed that older age, lower plasma DBP, hypoalbuminemia, and decreased uremic solute clearance (as indexed by lower Kt/V) were at an increased of dying. Considering all these variables for multivariate Cox regression analysis, DBP remains an independent predictor of 4-year mortality in the current study’s HD patients (Table 4).

**Table 4:** Cox proportional hazard model for prognosis of HD patients at a four-year follow-up.

		Univariate^a^				Multivariate^a^		
Factor^b^		95% CI^c^				95% CI		
	HR^d^	Lower	Upper	*p* Value	HR	Lower	Upper	*p* Value
ELISA - Vitamin D binding protein, 10 mg/L	0.895	0.817	0.980	0.013*	0.843	0.756	0.951	0.006
Age, year	1.045	1.003	1.089	0.037*	1.048	1.005	1.092	0.027
Male gender, %	1.445	0.538	3.883	0.465				
Dialysis vintage, years	0.900	0.800	1.013	0.080*				
Body weight, Kg	1.046	0.986	1.109	0.137				
Height, cm	1.048	0.987	1.113	0.129				
Body mass index, kg/m^2^	1.040	0.889	1.217	0.620				
Systolic blood pressure, mmHg	1.011	0.978	1.045	0.514				
Diastolic blood pressure, mmHg	1.008	0.942	1.079	0.823				
Smoking index	1.167	0.795	1.712	0.430				
Medications								
ACE inhibitor/ARB, %	1.132	0.403	3.183	0.936				
Vitamin D_3_, %	1.079	0.347	3.353	0.895				
Laboratory data								
Leukocyte count, ×10^3^/µL	1.000	0.999	1.000	0.117				
Hemoglobin, g/dL	1.011	0.684	1.495	0.954				
Hematocrit, %	1.012	0.887	1.154	0.863				
Albumin, g/dL	0.162	0.020	1.328	0.090*	0.064	0.008	0.574	0.012
Blood urea nitrogen, mg/dL	1.005	0.973	1.038	0.752				
Creatinine, mg/dL	1.134	0.648	1.983	0.660				
Sodium, meq/dL	1.023	0.835	1.254	0.826				
Potassium, meq/dL	1.464	0.721	2.973	0.291				
Fasting blood sugar, mg/dL	1.016	0.990	1.042	0.226				
Total cholesterol, mg/dL	0.998	0.985	1.012	0.777				
HDL-C, mg/dL	1.001	0.972	1.032	0.939				
LDL-C, mg/dL	0.992	0.980	1.004	0.210				
ApoB, mg/dL	0.985	0.966	1.005	0.147				
Lp(a), mg/dL	1.005	0.947	1.067	0.875				
Triglycerides, mg/dL	1.001	0.997	1.005	0.557				
Uric acid, mg/dL	0.821	0.576	1.170	0.275				
Alanine aminotransferase, U/L	1.008	0.982	1.036	0.542				
γ-glutamyl transferase, U/L	0.999	0.982	1.017	0.906				
Alkaline phosphatase, U/L	1.004	0.997	1.011	0.231				
Calcium, mg/dL	0.591	0.332	1.052	0.074*				
Phosphate, mg/dL	1.162	0.789	1.713	0.447				
Calcium × Phosphate product	1.003	0.964	1.044	0.876				
Intact-parathyroid hormone, pg/mL	1.001	1.000	1.003	0.157				
25(OH) Vitamin D, ng/mL	1.033	0.973	1.096	0.289				
Kt/V	0.026	0.002	0.545	0.124				

a Values are expressed as mean ± SD or percent.

b ACE, angiotensin converting enzyme; ARB, angiotensin receptor blocker.

c CI, confidence interval.

d HR, hazard ratio.

* *p*<0.10 in univariate Cox regression analysis.

## Discussion

Chronic HD patients are under constant exposure to uremic toxins and hemodynamic stress caused by fluctuations in blood pressure and fluid status. HD patients who can survive long and remain in stable conditions might be likened to centenarians in the general population. We are curious as to whether their plasma proteomes are altered, or whether certain protective factors might exist to endure the prolonged deleterious uremic milieu. By harnessing the power of 2-DE-based comparative proteomic techniques, we profiled plasma protein changes in HD patients with different dialysis vintages and identified 9 differentially expressed proteins in the current study’s LHD survivors. These protein profiles include increased DBP and decreased clusterin, apolipoprotein A-IV, haptoglobin, hemopexin, complement factors B and H, and altered isoform profiles of α1-antitrypsin and fibrinogen γ. We speculated that these protein changes might be related to intricate influences caused by adsorption [13] or elimination [14] of proteins by HD procedure, that is, altered catabolism resultant from deranged enzyme activities in the uremic state, and post-translational modifications by increased oxidative [15] and carbonyl stress [16] throughout the disease process.

By binding vitamin D, DBP is well recognized for regulating calcium-phosphate homeostasis [17] and exerting great impacts on cardiovascular calcifications [18]. It has been noted that, in addition to transporting vitamin D and its metabolites, DBP plays crucial roles in inflammation and immune reactions by mediating chemotaxis [19] and macrophage activation [20]. Moreover, DBP constitutes an important extracellular actin-scavenger system that eliminates actin released from cell lysis [21]. Without clearance, these accumulated extracellular actin filaments are extremely detrimental because of microvasculature blockage, and consequently leads to organ dysfunction. Decreased plasma DBP has also been reported to affect patient prognoses in multiple organ failure [22], and to fulminant hepatic failure [23]. Because of our observations of higher DBP levels in long-term surviving HD patients, and because lower DBP is related to increased risk of 4-year mortality, we reasoned that DBP might exert protective effects on HD.

Clusterin is a highly conserved and ubiquitously expressed secretory glycoprotein that regulates lipid transport and local deposition. Augmented clusterin expression has been demonstrated in human atherosclerotic lesions [24] and in endothelial cell cultures exposed to laminar shear stress [25]. An elevated serum clusterin level was observed in patients with coronary artery disease and myocardial infarction [26]. Therefore, clusterin was proposed to be an index of underlying cardiovascular damage. Clusterin is also a senescence biomarker that is upregulated during replicative and stress-induced premature senescence [27]. It could interact with stress-induced extracellular amyloid aggregations [28]. Our observation of lower plasma clusterin expressed in LHD survivors implies that these specific patients might develop certain protective mechanisms to resist hemodynamics and uremic stress with less accumulated damages during the course of HD.

We found that the current study’s LHD patients remained not refrained from injuries imposed by the uremic milieu. Their plasma revealed downregulation of certain cardiovascular-protective proteins such as apolipoprotein A-IV, haptoglobin, and hemopexin, which could explain the accelerated atherosclerosis process accompanying HD [29]. Apolipoprotein A-IV is an anti-atherogenic factor that participates in the reverse transport of cholesterol from peripheral cells to the liver and other steroidogenic organs, and acts as a potent inhibitor of lipid oxidation [30]. Decreased plasma apolipoprotein A-IV has also been demonstrated in patients with coronary artery disease [31]. Both haptoglobin and hemopexin are heme-binding glycoproteins that protect the body from hemoglobin-induced oxidative damage, nitric oxide toxicity, and pro-inflammatory effects induced by intravascular hemolysis. Haptoglobin also functions as a chaperone that inhibits oxidation-induced misfolding of extracellular proteins, and thus, exerts anti-inflammatory effects. In addition to binding heme, hemopexin could suppress neutrophil adhesion and phagocytosis. We believe that the downregulation of haptoglobin and hemopexin might reflect an exhausted antioxidant reserve in LHD patients while counteracting the persistent deranged redox state and inflammatory stresses.

Repetitive contact of blood with HD devices could activate the complement system and contribute to the lower level of complement factors B and H in the current study’s LHD patients [32]. The complement system is the main mediator of innate immunity and contributes to the recognition, opsonization, and lysis of microorganisms. Therefore, HD patients with decreased factors B and H are immunocompromised, susceptible to bacterial infections, and in a state of chronic inflammation [33].

Altered isoform patterns of plasma α1-antitrypsin and fibrinogen γ were demonstrated in the study’s HD patients with different dialysis vintages. α1-Antitrypsin is a potent inhibitor of several proteolytic enzymes that inhibit neutrophil superoxide production [34]. α1-Antitrypsin is related to the atherogenesis process, and different genotypes/phenotypes were related to diverse ischemic cerebrovascular and cardiovascular disease risks. Furthermore, serum α1-antitrypsin has been reported to be an important index of chronic inflammation in HD patients [35]. Exogenous administration of α1-antitrypsin could confer protection against ischemic/reperfusion injury [36]. Because HD is an ischemia-reperfusion process, α1-antitrypsin might serve as another therapeutic target.

Fibrinogen, the precursor of fibrin, is a 6-chain protein comprising 2 sets of the 3 polypeptide chains α, β, and γ. The γ chain interacts with other fibrin(ogen) molecules, coagulation factors, growth factors, and integrins. Therefore, fibrinogen γ prominently participates in platelet aggregation, coagulation, clot retraction, thrombosis, and inflammation [37]. The differential expression patterns of plasma α1-antitrypsin and fibrinogen γ chain isoforms have also been observed in other diseases. Mateos-Caceres et al. reported the differential expression of α1-antitrypsin and fibrinogen γ chain isoforms between the plasma of acute myocardial infarction and unstable angina patients [38]. We believe that these isoforms might reflect the disparate host responses to diverse pathological processes of HD and they merit further investigation.

High performance HD or more prolonged HD were expected to cause more efficient removal of uremic toxins. However, it might concurrently lead to the loss of plasma proteins as DBP [39]. To explore the impacts of dialysis efficiency on serum protein profiles, Hallbauer et al. [40] conducted a 10-week cross-over study of high- and low-flux dialysis treatments with the identical membrane material in 16 ESRD patients. The serum protein profiles were not altered by the increased pore size of HD membrane in the high performance HD. They reasoned that there might be no significant permeability differences in the dialysis and the clearance of proteins by HD might be compensated by de novo production. Complimentary to the proteomic researches, the clinical landmark study of HEMO neither obtained significant beneficial effects on patients’ survival by increasing higher dialysis dose or adopting the high-flux dialysis membranes [41]. It is important to perform long-term studies by using more advanced proteomic techniques to elucidate the effects of HD procedure on plasma proteome.

In conclusion, we observed that several altered plasma proteins in LHD survivors were related to signaling moieties of accelerated atherosclerosis, defective antioxidative activity, increased inflammation/infection, and organ dysfunction. Specifically, lower plasma DBP levels might be a predictor of cardiovascular mortality in HD patients. Further large-scale studies are warranted to consolidate this protein as a biomarker in prognoses, and to elucidate their roles in salient uremic milieus.

## Supporting Information

Figure S1 **Protein identification details of each spot.**
(PDF)

Data S1 The compressed file of all MS raw data for protein identification.
(RAR)
