# Supplementary material for: Plasma Protein Characteristics of Long-Term Hemodialysis Survivors
Source: PLoS One. 2012 Jul 6;7(7):e40232. doi: 10.1371/journal.pone.0040232 (PMC3391220; doi:10.1371/journal.pone.0040232)
Supplement: Figure S1 — Protein identification details of each spot. (PDF) [file pone.0040232.s001.pdf]

Protein Identification Details

Spot 1

Haptoglobin

MASCOT Search Results

Protein View: gi|1212947

haptoglobin [Homo sapiens]

**Database:**	NCBInr
**Score:**	617
**Nominal mass (M_r):**	38427
**Calculated pI:**	6.27
**Taxonomy:**	Homo sapiens

This protein sequence matches the following other entries:

- gi|111306445 from Homo sapiens

Sequence similarity is available as an NCBI BLAST search of gi|1212947 against nr.

Search parameters

**MS data file:**	Spot-1-051004 Select 1-D11.pkl
**Enzyme:**	Trypsin: cuts C-term side of KR unless next residue is P.
**Variable modifications:**	Carbamidomethyl (C), Oxidation (M)

Protein sequence coverage: 37%

Matched peptides shown in ***bold red***.

```
  1 MSALGAVIAL LLWGQLFAVD SGNDVTDIAD DGCPKPPEIA HGYVEHSVRY
 51 QCKNYYKLRT EGDGVYTLND KKQWINKAVG DKLPECEAVC GKPKNPANPV
101 QRILGGHLDA KGSFPWQAKM VSHHNLTTGA TLINEQWLLT TAKNLFLNHS
151 ENATAKDIAP TLTLYVGKKQ LVEIEKVVLH PNYSQVDIGL IKLKQKVSVN
201 ERVMPICLPS KDYAEVGRVG YVSGWGRNAN FKFTDHLKYV MLPVADQDQC
251 IRHYEGSTVP EKKTPKSPVG VQPILNEHTF CAGMSKYQED TCYGDAGSAF
301 AVHDLEEDTW YATGILSFDK SCAVAEYGVY VKVTSIQDWV QKTIAEN
```

Unformatted sequence string: 347 residues (for pasting into other applications).

Sort peptides by ◉ Residue Number ○ Increasing Mass ○ Decreasing Mass

Show predicted peptides also

Query	Start – End	Observed	Mr(expt)	Mr(calc)	Delta	M	Score	Expect	Rank	U	Peptide
17	103 – 111	462.3876	922.7606	922.5236	0.2370	0	43	3	1	U	R.ILGGHLDAK.G
15	112 – 119	460.8518	919.6890	919.4552	0.2338	0	28	1.2e+02	1		K.GSFPWQAK.M
32	157 – 168	646.0010	1289.9874	1289.7231	0.2643	0	50	0.65	1	U	K.DIAPTLTLYVGK.K
19	195 – 202	480.3856	958.7566	958.5196	0.2371	1	38	13	5	U	K.QKVSVNER.V
22	203 – 211	530.9037	1059.7928	1059.5457	0.2472	0	19	8e+02	5		R.VMPICLPSK.D + Carbamidomethyl (C); Oxidation (M)
47	203 – 218	617.7728	1850.2966	1849.9066	0.3899	1	82	0.00028	1	U	R.VMPICLPSKDYAEVGR.V + Carbamidomethyl (C); Oxidation (M)
48	203 – 218	617.7743	1850.3011	1849.9066	0.3944	1	55	0.15	1	U	R.VMPICLPSKDYAEVGR.V + Carbamidomethyl (C); Oxidation (M)
21	219 – 227	490.8699	979.7252	979.4876	0.2377	0	51	0.51	1	U	R.VGYVSGWGR.N
39	239 – 252	854.5561	1707.0976	1706.8120	0.2856	0	65	0.016	1	U	K.YVMLPVADQDQCIR.H + Carbamidomethyl (C)
40	239 – 252	862.5505	1723.0864	1722.8069	0.2795	0	54	0.2	1	U	K.YVMLPVADQDQCIR.H + Carbamidomethyl (C); Oxidation (M)
41	239 – 252	862.5696	1723.1246	1722.8069	0.3177	0	32	29	1	U	K.YVMLPVADQDQCIR.H + Carbamidomethyl (C); Oxidation (M)
42	239 – 252	575.3962	1723.1668	1722.8069	0.3598	0	33	25	1	U	K.YVMLPVADQDQCIR.H + Carbamidomethyl (C); Oxidation (M)
24	253 – 262	573.8962	1145.7778	1145.5353	0.2426	0	30	67	1	U	R.HYEGSTVPEK.K
29	253 – 263	637.9422	1273.8698	1273.6302	0.2396	1	24	2.4e+02	1	U	R.HYEGSTVPEKK.T
30	253 – 263	425.6553	1273.9441	1273.6302	0.3138	1	34	24	1	U	R.HYEGSTVPEKK.T
50	267 – 286	730.1642	2187.4708	2187.0453	0.4255	0	51	0.35	1	U	K.SPVGVQPILNEHTFCAGMSK.Y + Carbamidomethyl (C); Oxidation (M)
51	267 – 286	730.1674	2187.4804	2187.0453	0.4351	0	30	44	1	U	K.SPVGVQPILNEHTFCAGMSK.Y + Carbamidomethyl (C); Oxidation (M)
34	321 – 332	673.4565	1344.8984	1344.6384	0.2601	0	45	2.1	1	U	K.SCAVAEYGVYVK.V + Carbamidomethyl (C)
35	321 – 332	673.4580	1344.9014	1344.6384	0.2631	0	18	9.6e+02	1	U	K.SCAVAEYGVYVK.V + Carbamidomethyl (C)
36	321 – 332	673.4712	1344.9278	1344.6384	0.2895	0	70	0.0058	1	U	K.SCAVAEYGVYVK.V + Carbamidomethyl (C)
26	333 – 342	602.4487	1202.8828	1202.6295	0.2533	0	56	0.17	1	U	K.VTSIQDWVQK.T

```
LOCUS       CAA25926                 347 aa            linear   PRI 14-NOV-2006
DEFINITION  haptoglobin [Homo sapiens].
ACCESSION   CAA25926
VERSION     CAA25926.1  GI:1212947
DBSOURCE    embl accession X01793.1
            embl accession X01786.1
            embl accession X02206.1
            embl accession X01789.1
            embl accession X01791.1
KEYWORDS    .
SOURCE      Homo sapiens (human)
  ORGANISM  Homo sapiens
            Eukaryota; Metazoa; Chordata; Craniata; Vertebrata; Euteleostomi;
            Mammalia; Eutheria; Euarchontoglires; Primates; Haplorrhini;
            Catarrhini; Hominidae; Homo.
REFERENCE   1  (residues 1 to 347)
  AUTHORS   Bensi,G., Raugei,G., Klefenz,H. and Cortese,R.
  TITLE     Structure and expression of the human haptoglobin locus
  JOURNAL   EMBO J. 4 (1), 119-126 (1985)
   PUBMED   4018023
FEATURES             Location/Qualifiers
     source          1..347
                     /organism="Homo sapiens"
                     /db_xref="taxon:9606"
     Protein         1..347
                     /product="haptoglobin"
     Region          33..87
                     /region_name="CCP"
                     /note="Complement control protein (CCP) modules (aka short
```

```
Site            order(43,53)
                /site_type="other"
                /note="receptor-ligand interactions"
                /db_xref="CDD:153056"
Region          103..343
                /region_name="Tryp_SPc"
                /note="Trypsin-like serine protease; Many of these are
                synthesized as inactive precursor zymogens that are
                cleaved during limited proteolysis to generate their
                active forms. Alignment contains also inactive enzymes
                that have substitutions of the catalytic triad...;
                cd00190"
                /db_xref="CDD:29152"
Site            103
                /site_type="cleavage"
                /db_xref="CDD:29152"
Site            order(143,187,296)
                /site_type="active"
                /db_xref="CDD:29152"
Site            order(290,317,319)
                /site_type="other"
                /note="substrate binding sites [chemical binding]"
                /db_xref="CDD:29152"
CDS             1..347
                /gene="Hp1"
                /coded_by="join(X01793.1:608..612,X01786.1:13..95,
                X02206.1:7..108,X01789.1:18..92,X01791.1:11..789)"
                /db_xref="GDB:119314"
                /db_xref="GOA:P00738"
                /db_xref="HGNC:5141"
                /db_xref="InterPro:IPR000436"
                /db_xref="InterPro:IPR001254"
                /db_xref="InterPro:IPR001314"
                /db_xref="InterPro:IPR008292"
                /db_xref="InterPro:IPR009003"
                /db_xref="InterPro:IPR016060"
                /db_xref="UniProtKB/Swiss-Prot:P00738"
```

Spot 2

Haptoglobin

MATRIX SCIENCE MASCOT Search Results

Protein View: gi|1212947

haptoglobin [Homo sapiens]

**Database:**	NCBInr
**Score:**	79
**Expect:**	0.003
**Nominal mass (M_r):**	38427
**Calculated pI:**	6.27
**Taxonomy:**	Homo sapiens

This protein sequence matches the following other entries:

- gi|111306445 from Homo sapiens

Sequence similarity is available as an NCBI BLAST search of gi|1212947 against nr.

Search parameters

**MS data file:**	peaklist.xml
**Enzyme:**	Trypsin: cuts C-term side of KR unless next residue is P.
**Variable modifications:**	Carbamidomethyl (C), Oxidation (M)
**Mass values searched:**	12
**Mass values matched:**	9

Protein sequence coverage: 22%

Matched peptides shown in ***bold red***.

```
  1 MSALGAVIAL LLWGQLFAVD SGNDVTDIAD DGCPKPPEIA HGYVEHSVRY
 51 QCKNYYKLRT EGDGVYTLND KKQWINKAVG DKLPECEAVC GKPKNPANPV
101 QRILGGHLDA KGSFPWQAKM VSHHNLTTGA TLINEQWLLT TAKNLFLNHS
151 ENATAKDIAP TLTLYVGKKQ LVEIEKVVLH PNYSQVDIGL IKLKQKVSVN
201 ERVMPICLPS KDYAEVGRVG YVSGWGRNAN FKFTDHLKYV MLPVADQDQC
251 IRHYEGSTVP EKKTPKSPVG VQPILNEHTF CAGMSKYQED TCYGDAGSAF
301 AVHDLEEDTW YATGILSFDK SCAVAEYGVY VKVTSIQDWV QKTIAEN
```

Unformatted sequence string: 347 residues (for pasting into other applications).

Sort peptides by ◉ Residue Number ○ Increasing Mass ○ Decreasing Mass

Show predicted peptides also

Start – End	Observed	Mr(expt)	Mr(calc)	ppm	M	Peptide
112 – 119	920.3712	919.3639	919.4552	-99.3	0	K.GSFPWQAK.M
203 – 218	1834.8245	1833.8172	1833.9117	-51.5	1	R.VMPICLPSKDYAEVGR.V + Carbamidomethyl (C)
203 – 218	1850.8180	1849.8107	1849.9066	-51.8	1	R.VMPICLPSKDYAEVGR.V + Carbamidomethyl (C); Oxidation (M)
219 – 227	980.4217	979.4144	979.4876	-74.7	0	R.VGYVSGWGR.N
239 – 252	1707.7252	1706.7179	1706.8120	-55.1	0	K.YVMLPVADQDQCIR.H + Carbamidomethyl (C)
239 – 252	1723.7126	1722.7054	1722.8069	-59.0	0	K.YVMLPVADQDQCIR.H + Carbamidomethyl (C); Oxidation (M)
267 – 286	2171.9331	2170.9258	2171.0504	-57.4	0	K.SPVGVQPILNEHTFCAGMSK.Y + Carbamidomethyl (C)
267 – 286	2187.9358	2186.9285	2187.0453	-53.4	0	K.SPVGVQPILNEHTFCAGMSK.Y + Carbamidomethyl (C); Oxidation (M)
321 – 332	1345.5759	1344.5687	1344.6384	-51.9	0	K.SCAVAEYGVYVK.V + Carbamidomethyl (C)

No match to: 984.4226, 1012.4109, 2133.8994

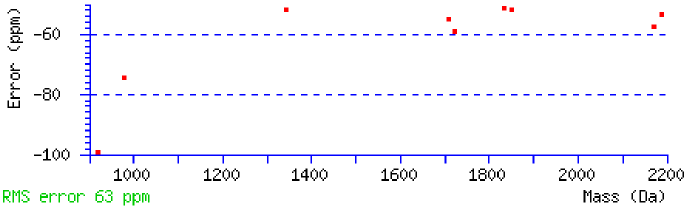


```
LOCUS       CAA25926                 347 aa            linear   PRI 14-NOV-2006
DEFINITION  haptoglobin [Homo sapiens].
ACCESSION   CAA25926
VERSION     CAA25926.1  GI:1212947
DBSOURCE    embl accession X01793.1
            embl accession X01786.1
            embl accession X02206.1
            embl accession X01789.1
            embl accession X01791.1
KEYWORDS    .
SOURCE      Homo sapiens (human)
  ORGANISM  Homo sapiens
            Eukaryota; Metazoa; Chordata; Craniata; Vertebrata; Euteleostomi;
```

```
  AUTHORS   Bensi,G., Raugei,G., Klefenz,H. and Cortese,R.
  TITLE     Structure and expression of the human haptoglobin locus
  JOURNAL   EMBO J. 4 (1), 119-126 (1985)
   PUBMED   4018023
FEATURES             Location/Qualifiers
     source          1..347
                     /organism="Homo sapiens"
                     /db_xref="taxon:9606"
     Protein         1..347
                     /product="haptoglobin"
     Region          33..87
                     /region_name="CCP"
                     /note="Complement control protein (CCP) modules (aka short
                     consensus repeats SCRs or SUSHI repeats) have been
                     identified in several proteins of the complement system;
                     cd00033"
                     /db_xref="CDD:153056"
     Site            order(43,53)
                     /site_type="other"
                     /note="receptor-ligand interactions"
                     /db_xref="CDD:153056"
     Region          103..343
                     /region_name="Tryp_SPc"
                     /note="Trypsin-like serine protease; Many of these are
                     synthesized as inactive precursor zymogens that are
                     cleaved during limited proteolysis to generate their
                     active forms. Alignment contains also inactive enzymes
                     that have substitutions of the catalytic triad...;
                     cd00190"
                     /db_xref="CDD:29152"
     Site            103
                     /site_type="cleavage"
                     /db_xref="CDD:29152"
     Site            order(143,187,296)
                     /site_type="active"
                     /db_xref="CDD:29152"
     Site            order(290,317,319)
                     /site_type="other"
                     /note="substrate binding sites [chemical binding]"
                     /db_xref="CDD:29152"
     CDS             1..347
                     /gene="Hp1"
                     /coded_by="join(X01793.1:608..612,X01786.1:13..95,
                     X02206.1:7..108,X01789.1:18..92,X01791.1:11..789)"
                     /db_xref="GDB:119314"
                     /db_xref="GOA:P00738"
                     /db_xref="HGNC:5141"
                     /db_xref="InterPro:IPR000436"
                     /db_xref="InterPro:IPR001254"
                     /db_xref="InterPro:IPR001314"
                     /db_xref="InterPro:IPR008292"
                     /db_xref="InterPro:IPR009003"
                     /db_xref="InterPro:IPR016060"
                     /db_xref="UniProtKB/Swiss-Prot:P00738"
```

Spot 3

Haptoglobin

MASCOT Search Results

Protein View: gi|1212947

haptoglobin [Homo sapiens]

**Database:**	NCBInr
**Score:**	337
**Nominal mass (M_r):**	38427
**Calculated pI:**	6.27
**Taxonomy:**	Homo sapiens

This protein sequence matches the following other entries:

- gi|111306445 from Homo sapiens

Sequence similarity is available as an NCBI BLAST search of gi|1212947 against nr.

Search parameters

**MS data file:**	Spot-3-060315 CYWu-5.pkl
**Enzyme:**	Trypsin: cuts C-term side of KR unless next residue is P.
**Variable modifications:**	Carbamidomethyl (C), Oxidation (M)

Protein sequence coverage: 24%

Matched peptides shown in ***bold red***.

1 MSALGAVIAL LLWGQLFAVD SGNDVTDIAD DGCPKPPEIA HGYVEHSVRY
51 QCKNYYKLRT EGDGVYTLND KKQWINKAVG DKLPECEAVC GKPKNPANPV
101 QR**ILGGHLDA KGSFPWQAK**M VSHHNLTTGA TLINEQWLLT TAKNLFLNHS
151 ENATAKDIAP TLTLYVGKKQ LVEIEKVVLH PNYSQVDIGL IKLKQKVSVN
201 ERVMPICLPS KDYAEVGRVG YVSGWGRNAN FKFTDHLK**YV MLPVADQDQC**
251 **IRHYEGSTVP EKK**TPK**SPVG VQPILNEHTF CAGMSK**YQED TCYGDAGSAF
301 AVHDLEEDTW YATGILSFDK **SCAVAEYGVY VKVTSIQDWV QK**TIAEN

Unformatted sequence string: 347 residues (for pasting into other applications).

Sort peptides by ◉ Residue Number ○ Increasing Mass ○ Decreasing Mass

Show predicted peptides also

Query	Start – End	Observed	Mr(expt)	Mr(calc)	Delta	M	Score	Expect	Rank	U	Peptide
10	103 – 111	462.1581	922.3016	922.5236	-0.2220	0	34	24	1		R.ILGGHLDAK.G
9	112 – 119	460.6281	919.2416	919.4552	-0.2136	0	23	3.8e+02	4		K.GSFPWQAK.M
24	239 – 252	862.1430	1722.2714	1722.8069	-0.5355	0	34	22	1		K.YVMLPVADQDQCIR.H + Carbamidomethyl (C); Oxidation (M)
18	253 – 262	573.6257	1145.2368	1145.5353	-0.2984	0	32	42	1		R.HYEGSTVPEK.K
21	253 – 263	425.4430	1273.3072	1273.6302	-0.3231	1	33	35	1		R.HYEGSTVPEKK.T
25	267 – 286	724.4756	2170.4050	2171.0504	-0.6454	0	26	1e+02	1	U	K.SPVGVQPILNEHTFCAGMSK.Y + Carbamidomethyl (C)
26	267 – 286	729.7969	2186.3689	2187.0453	-0.6764	0	79	0.00055	1	U	K.SPVGVQPILNEHTFCAGMSK.Y + Carbamidomethyl (C); Oxidation (M)
23	321 – 332	673.1230	1344.2314	1344.6384	-0.4069	0	55	0.21	1		K.SCAVAEYGVYVK.V + Carbamidomethyl (C)
19	333 – 342	602.1563	1202.2980	1202.6295	-0.3315	0	51	0.56	1		K.VTSIQDWVQK.T

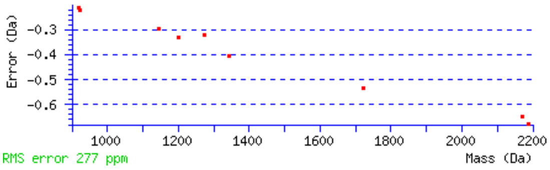

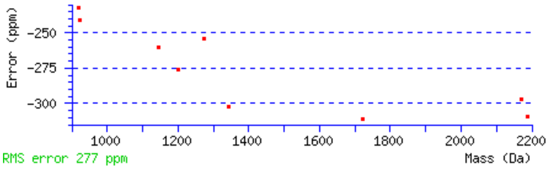


```
LOCUS       CAA25926                 347 aa            linear   PRI 14-NOV-2006
DEFINITION  haptoglobin [Homo sapiens].
ACCESSION   CAA25926
VERSION     CAA25926.1  GI:1212947
DBSOURCE    embl accession X01793.1
            embl accession X01786.1
            embl accession X02206.1
            embl accession X01789.1
            embl accession X01791.1
KEYWORDS    .
SOURCE      Homo sapiens (human)
  ORGANISM  Homo sapiens
            Eukaryota; Metazoa; Chordata; Craniata; Vertebrata; Euteleostomi;
            Mammalia; Eutheria; Euarchontoglires; Primates; Haplorrhini;
            Catarrhini; Hominidae; Homo.
REFERENCE   1  (residues 1 to 347)
  AUTHORS   Bensi,G., Raugei,G., Klefenz,H. and Cortese,R.
  TITLE     Structure and expression of the human haptoglobin locus
  JOURNAL   EMBO J. 4 (1), 119-126 (1985)
   PUBMED   4018023
FEATURES             Location/Qualifiers
     source          1..347
                     /organism="Homo sapiens"
                     /db_xref="taxon:9606"
     Protein         1..347
                     /product="haptoglobin"
     Region          33..87
                     /region_name="CCP"
                     /note="Complement control protein (CCP) modules (aka short
                     consensus repeats SCRs or SUSHI repeats) have been
                     identified in several proteins of the complement system;
                     cd00033"
                     /db_xref="CDD:153056"
     Site            order(43,53)
                     /site_type="other"
                     /note="receptor-ligand interactions"
                     /db_xref="CDD:153056"
     Region          103..343
                     /region_name="Tryp_SPc"
                     /note="Trypsin-like serine protease; Many of these are
                     synthesized as inactive precursor zymogens that are
                     cleaved during limited proteolysis to generate their
                     active forms. Alignment contains also inactive enzymes
```

```
     Site            103
                     /site_type="cleavage"
                     /db_xref="CDD:29152"
     Site            order(143,187,296)
                     /site_type="active"
                     /db_xref="CDD:29152"
     Site            order(290,317,319)
                     /site_type="other"
                     /note="substrate binding sites [chemical binding]"
                     /db_xref="CDD:29152"
     CDS             1..347
                     /gene="Hp1"
                     /coded_by="join(X01793.1:608..612,X01786.1:13..95,
                     X02206.1:7..108,X01789.1:18..92,X01791.1:11..789)"
                     /db_xref="GDB:119314"
                     /db_xref="GOA:P00738"
                     /db_xref="HGNC:5141"
                     /db_xref="InterPro:IPR000436"
                     /db_xref="InterPro:IPR001254"
                     /db_xref="InterPro:IPR001314"
                     /db_xref="InterPro:IPR008292"
                     /db_xref="InterPro:IPR009003"
                     /db_xref="InterPro:IPR016060"
                     /db_xref="UniProtKB/Swiss-Prot:P00738"
```

Spot 4

Haptoglobin

{MATRIX SCIENCE} MASCOT Search Results

Protein View: gi|1212947

haptoglobin [Homo sapiens]

**Database:**	NCBInr
**Score:**	349
**Nominal mass (M_r):**	38427
**Calculated pI:**	6.27
**Taxonomy:**	Homo sapiens

This protein sequence matches the following other entries:

- gi|111306445 from Homo sapiens

Sequence similarity is available as an NCBI BLAST search of gi|1212947 against nr.

Search parameters

**MS data file:**	Spot-4-060316 CYWu-5.pkl
**Enzyme:**	Trypsin: cuts C-term side of KR unless next residue is P.
**Variable modifications:**	Carbamidomethyl (C), Oxidation (M)

Protein sequence coverage: 27%

Matched peptides shown in ***bold red***.

```
  1 MSALGAVIAL LLWGQLFAVD SGNDVTDIAD DGCPKPPEIA HGYVEHSVRY
 51 QCKNYYKLRT EGDGVYTLND KKQWINKAVG DKLPECEAVC GKPKNPANPV
101 QRILGGHLDA KGSFPWQAKM VSHHNLTTGA TLINEQWLLT TAKNLFLNHS
151 ENATAKDIAP TLTLYVGKKQ LVEIEKVVLH PNYSQVDIGL IKLKQKVSVN
201 ERVMPICLPS KDYAEVGRVG YVSGWGRNAN FKFTDHLKYV MLPVADQDQC
251 IRHYEGSTVP EKKTPKSPVG VQPILNEHTF CAGMSKYQED TCYGDAGSAF
301 AVHDLEEDTW YATGILSFDK SCAVAEYGVY VKVTSIQDWV QKTIAEN
```

Unformatted sequence string: 347 residues (for pasting into other applications).

Sort peptides by ◉ Residue Number ○ Increasing Mass ○ Decreasing Mass

Show predicted peptides also

Query	Start – End	Observed	Mr(expt)	Mr(calc)	Delta	M	Score	Expect	Rank	U	Peptide
12	103 – 111	462.3409	922.6672	922.5236	0.1436	0	28	1.1e+02	2	U	R.ILGGHLDAK.G
7	170 – 176	429.8282	857.6418	857.4858	0.1560	0	29	1.1e+02	1	U	K.QLVEIEK.V
17	203 – 211	530.8455	1059.6764	1059.5457	0.1308	0	24	2.6e+02	1	U	R.VMPICLPSK.D + Carbamidomethyl (C); Oxidation (M)
34	203 – 218	617.6960	1850.0662	1849.9066	0.1595	1	67	0.0091	1	U	R.VMPICLPSKDYAEVGR.V + Carbamidomethyl (C); Oxidation (M)
13	219 – 227	490.8095	979.6044	979.4876	0.1169	0	24	2.3e+02	2	U	R.VGYVSGWGR.N
14	219 – 227	490.8133	979.6120	979.4876	0.1245	0	35	19	3	U	R.VGYVSGWGR.N
27	253 – 263	425.6238	1273.8496	1273.6302	0.2193	1	37	13	1	U	R.HYEGSTVPEKK.T
36	267 – 286	730.0641	2187.1705	2187.0453	0.1252	0	34	17	1	U	K.SPVGVQPILNEHTFCAGMSK.Y + Carbamidomethyl (C); Oxidation (M)
29	321 – 332	673.3860	1344.7574	1344.6384	0.1191	0	51	0.44	1	U	K.SCAVAEYGVYVK.V + Carbamidomethyl (C)
25	333 – 342	602.3801	1202.7456	1202.6295	0.1161	0	44	2.9	1	U	K.VTSIQDWVQK.T

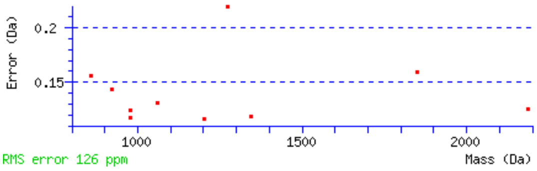

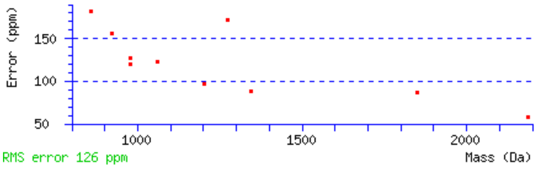


```
LOCUS       CAA25926                 347 aa            linear   PRI 14-NOV-2006
DEFINITION  haptoglobin [Homo sapiens].
ACCESSION   CAA25926
VERSION     CAA25926.1  GI:1212947
DBSOURCE    embl accession X01793.1
            embl accession X01786.1
            embl accession X02206.1
            embl accession X01789.1
            embl accession X01791.1
KEYWORDS    .
SOURCE      Homo sapiens (human)
  ORGANISM  Homo sapiens
            Eukaryota; Metazoa; Chordata; Craniata; Vertebrata; Euteleostomi;
            Mammalia; Eutheria; Euarchontoglires; Primates; Haplorrhini;
            Catarrhini; Hominidae; Homo.
REFERENCE   1  (residues 1 to 347)
  AUTHORS   Bensi,G., Raugei,G., Klefenz,H. and Cortese,R.
  TITLE     Structure and expression of the human haptoglobin locus
  JOURNAL   EMBO J. 4 (1), 119-126 (1985)
   PUBMED   4018023
FEATURES             Location/Qualifiers
     source          1..347
                     /organism="Homo sapiens"
                     /db_xref="taxon:9606"
     Protein         1..347
                     /product="haptoglobin"
     Region          33..87
                     /region_name="CCP"
                     /note="Complement control protein (CCP) modules (aka short
                     consensus repeats SCRs or SUSHI repeats) have been
                     identified in several proteins of the complement system;
                     cd00033"
                     /db_xref="CDD:153056"
     Site            order(43,53)
                     /site_type="other"
                     /note="receptor-ligand interactions"
                     /db_xref="CDD:153056"
     Region          103..343
                     /region_name="Tryp_SPc"
                     /note="Trypsin-like serine protease; Many of these are
                     synthesized as inactive precursor zymogens that are
                     cleaved during limited proteolysis to generate their
```

```
                    /db_xref="CDD:29152"
     Site           103
                    /site_type="cleavage"
                    /db_xref="CDD:29152"
     Site           order(143,187,296)
                    /site_type="active"
                    /db_xref="CDD:29152"
     Site           order(290,317,319)
                    /site_type="other"
                    /note="substrate binding sites [chemical binding]"
                    /db_xref="CDD:29152"
     CDS            1..347
                    /gene="Hp1"
                    /coded_by="join(X01793.1:608..612,X01786.1:13..95,
                    X02206.1:7..108,X01789.1:18..92,X01791.1:11..789)"
                    /db_xref="GDB:119314"
                    /db_xref="GOA:P00738"
                    /db_xref="HGNC:5141"
                    /db_xref="InterPro:IPR000436"
                    /db_xref="InterPro:IPR001254"
                    /db_xref="InterPro:IPR001314"
                    /db_xref="InterPro:IPR008292"
                    /db_xref="InterPro:IPR009003"
                    /db_xref="InterPro:IPR016060"
                    /db_xref="UniProtKB/Swiss-Prot:P00738"
```

Spot 5

Haptoglobin

MASCOT Search Results

Protein View: gi|1212947

haptoglobin [Homo sapiens]

**Database:**	NCBInr
**Score:**	47
**Expect:**	5.1
**Nominal mass (M_r):**	38427
**Calculated pI:**	6.27
**Taxonomy:**	Homo sapiens

This protein sequence matches the following other entries:

- gi|111306445 from Homo sapiens

Sequence similarity is available as an NCBI BLAST search of gi|1212947 against nr.

Search parameters

**MS data file:**	peaklist.xml
**Enzyme:**	Trypsin: cuts C-term side of KR unless next residue is P.
**Variable modifications:**	Carbamidomethyl (C), Oxidation (M)
**Mass values searched:**	9
**Mass values matched:**	5

Protein sequence coverage: 17%

Matched peptides shown in ***bold red***.

```
  1 MSALGAVIAL LLWGQLFAVD SGNDVTDIAD DGCPKPPEIA HGYVEHSVRY
 51 QCKNYYKLRT EGDGVYTLND KKQWINKAVG DKLPECEAVC GKPKNPANPV
101 QRILGGHLDA KGSFPWQAKM VSHHNLTTGA TLINEQWLLT TAKNLFLNHS
151 ENATAKDIAP TLTLYVGKKQ LVEIEKVVLH PNYSQVDIGL IKLKQKVSVN
201 ERVMPICLPS KDYAEVGRVG YVSGWGRNAN FKFTDHLKYV MLPVADQDQC
251 IRHYEGSTVP EKKTPKSPVG VQPILNEHTF CAGMSKYQED TCYGDAGSAF
301 AVHDLEEDTW YATGILSFDK SCAVAEYGVY VKVTSIQDWV QKTIAEN
```

Unformatted sequence string: 347 residues (for pasting into other applications).

Sort peptides by ◉ Residue Number ○ Increasing Mass ○ Decreasing Mass

Show predicted peptides also

Start – End	Observed	Mr(expt)	Mr(calc)	ppm	M	Peptide
203 – 218	1850.8667	1849.8594	1849.9066	-25.5	1	R.VMPICLPSKDYAEVGR.V + Carbamidomethyl (C); Oxidation (M)
219 – 227	980.4374	979.4301	979.4876	-58.7	0	R.VGYVSGWGR.N
239 – 252	1707.7500	1706.7427	1706.8120	-40.6	0	K.YVMLPVADQDQCIR.H + Carbamidomethyl (C)
239 – 252	1723.7589	1722.7516	1722.8069	-32.1	0	K.YVMLPVADQDQCIR.H + Carbamidomethyl (C); Oxidation (M)
267 – 286	2187.9885	2186.9812	2187.0453	-29.3	0	K.SPVGVQPILNEHTFCAGMSK.Y + Carbamidomethyl (C); Oxidation (M)

No match to: 1012.4271, 1725.7335, 1797.0228, 2210.0103

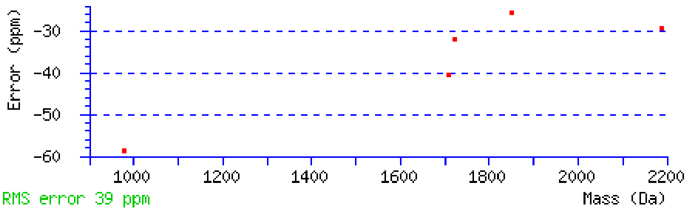

```
LOCUS       CAA25926                 347 aa            linear   PRI 14-NOV-2006
DEFINITION  haptoglobin [Homo sapiens].
ACCESSION   CAA25926
VERSION     CAA25926.1  GI:1212947
DBSOURCE    embl accession X01793.1
            embl accession X01786.1
            embl accession X02206.1
            embl accession X01789.1
            embl accession X01791.1
KEYWORDS    .
SOURCE      Homo sapiens (human)
  ORGANISM  Homo sapiens
            Eukaryota; Metazoa; Chordata; Craniata; Vertebrata; Euteleostomi;
            Mammalia; Eutheria; Euarchontoglires; Primates; Haplorrhini;
            Catarrhini; Hominidae; Homo.
REFERENCE   1  (residues 1 to 347)
  AUTHORS   Bensi,G., Raugei,G., Klefenz,H. and Cortese,R.
```

```
FEATURES             Location/Qualifiers
     source          1..347
                     /organism="Homo sapiens"
                     /db_xref="taxon:9606"
     Protein         1..347
                     /product="haptoglobin"
     Region          33..87
                     /region_name="CCP"
                     /note="Complement control protein (CCP) modules (aka short
                     consensus repeats SCRs or SUSHI repeats) have been
                     identified in several proteins of the complement system;
                     cd00033"
                     /db_xref="CDD:153056"
     Site            order(43,53)
                     /site_type="other"
                     /note="receptor-ligand interactions"
                     /db_xref="CDD:153056"
     Region          103..343
                     /region_name="Tryp_SPc"
                     /note="Trypsin-like serine protease; Many of these are
                     synthesized as inactive precursor zymogens that are
                     cleaved during limited proteolysis to generate their
                     active forms. Alignment contains also inactive enzymes
                     that have substitutions of the catalytic triad...;
                     cd00190"
                     /db_xref="CDD:29152"
     Site            103
                     /site_type="cleavage"
                     /db_xref="CDD:29152"
     Site            order(143,187,296)
                     /site_type="active"
                     /db_xref="CDD:29152"
     Site            order(290,317,319)
                     /site_type="other"
                     /note="substrate binding sites [chemical binding]"
                     /db_xref="CDD:29152"
     CDS             1..347
                     /gene="Hp1"
                     /coded_by="join(X01793.1:608..612,X01786.1:13..95,
                     X02206.1:7..108,X01789.1:18..92,X01791.1:11..789)"
                     /db_xref="GDB:119314"
                     /db_xref="GOA:P00738"
                     /db_xref="HGNC:5141"
                     /db_xref="InterPro:IPR000436"
                     /db_xref="InterPro:IPR001254"
                     /db_xref="InterPro:IPR001314"
                     /db_xref="InterPro:IPR008292"
                     /db_xref="InterPro:IPR009003"
                     /db_xref="InterPro:IPR016060"
                     /db_xref="UniProtKB/Swiss-Prot:P00738"
```

Mascot: http://www.matrixscience.com/

Spot 6

Clusterin

MATRIX SCIENCE MASCOT Search Results

Protein View: gi|32891795

clusterin (complement lysis inhibitor, SP-40,40, sulfated glycoprotein 2, testosterone-repressed prostate message 2, apolipoprotein J) [Homo sapiens]

**Database:**	NCBInr
**Score:**	97
**Nominal mass (M_r):**	52203
**Calculated pI:**	6.06
**Taxonomy:**	Homo sapiens

Sequence similarity is available as an NCBI BLAST search of gi|32891795 against nr.

Search parameters

**MS data file:**	Spot-6-060316 CYWu-8.pkl
**Enzyme:**	Trypsin: cuts C-term side of KR unless next residue is P.
**Variable modifications:**	Carbamidomethyl (C), Oxidation (M)

Protein sequence coverage: 6%

Matched peptides shown in ***bold red***.

1 MMKTLLLFVG LLLTWESGQV LGDQTVSDNE LQEMSNQGSK YVNK**EIQNAV**
51 **NGVK**QIKTLI EKTNEERK**TL** **LSNLEEAK**KK KEDALNETRE SETKLKELPG
101 VCNETMMALW EECKPCLKQT CMKFYARVCR SGSGLVGRQL EEFLNQSSPF
151 YFWMNGDRID SLLENDRQQT HMLDVMQDHF SRASSIIDEL FQDRFFTREP
201 QDTYHYLPFS LPHRRPHFFF PKSRIVRSLM PFSPYEPLNF HAMFQPFLEM
251 IHEAQQAMDI HFHSPAFQHP PTEFIREGDD DRTVCREIRH NSTGCLRMKD
301 QCDKCREILS VDCSTNNPSQ AKLRRELDES LQVAERLTRK YNELLKSYQW
351 KMLNTSSLLE QLNEQFNWVS RLANLTQGED QYYLRVTTVA SHTSDSDVPS
401 GVTEVVVKLF DSDPITVTVP VEVSRKNPK**F** **METVAEK**ALQ EYRKKHR

Unformatted sequence string: 447 residues (for pasting into other applications).

Sort peptides by (•) Residue Number ( ) Increasing Mass ( ) Decreasing Mass

Show predicted peptides also

Query	Start – End	Observed	Mr(expt)	Mr(calc)	Delta	M	Score	Expect	Rank	U	Peptide
8	45 – 54	536.2986	1070.5826	1070.5720	0.0106	0	20	10	1	U	K.EIQNAVNGVK.Q
9	69 – 78	559.2991	1116.5836	1116.6026	-0.0190	0	34	0.4	1	U	K.TLLSNLEEAK.K
6	430 – 437	485.7446	969.4746	969.4477	0.0269	0	43	0.04	1	U	K.FMETVAEK.A + Oxidation (M)

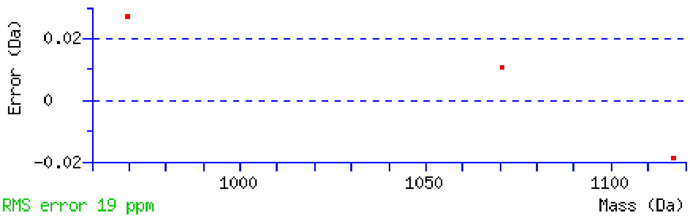

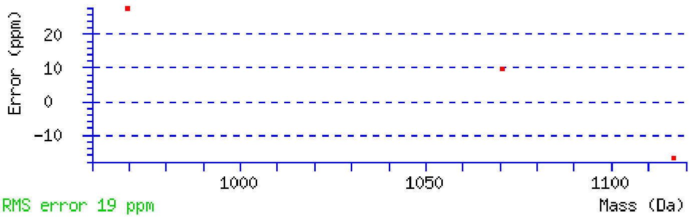


```
LOCUS       AAP88927                 447 aa            linear   PRI 22-JUL-2003
DEFINITION  clusterin (complement lysis inhibitor, SP-40,40, sulfated
            glycoprotein 2, testosterone-repressed prostate message 2,
            apolipoprotein J) [Homo sapiens].
ACCESSION   AAP88927
VERSION     AAP88927.1  GI:32891795
DBSOURCE    accession AY341244.1
KEYWORDS    .
SOURCE      Homo sapiens (human)
  ORGANISM  Homo sapiens
            Eukaryota; Metazoa; Chordata; Craniata; Vertebrata; Euteleostomi;
            Mammalia; Eutheria; Euarchontoglires; Primates; Haplorrhini;
            Catarrhini; Hominidae; Homo.
REFERENCE   1  (residues 1 to 447)
  AUTHORS   Rieder,M.J., Livingston,R.J., Daniels,M.R., Chung,M.-W.,
            Miyamoto,K.E., Nguyen,C.P., Nguyen,D.A., Poel,C.L., Robertson,P.D.,
            Schackwitz,W.S., Sherwood,J.K., Witrak,L.A. and Nickerson,D.A.
  TITLE     Direct Submission
  JOURNAL   Submitted (11-JUL-2003) Genome Sciences, University of Washington,
            1705 NE Pacific, Seattle, WA 98195, USA
COMMENT     Method: conceptual translation supplied by author.
FEATURES             Location/Qualifiers
     source          1..447
```

```
Protein         1..447
                /product="clusterin (complement lysis inhibitor, SP-40,40,
                sulfated glycoprotein 2, testosterone-repressed prostate
                message 2, apolipoprotein J)"
Region          23..227
                /region_name="CLb"
                /note="CLUSTERIN Beta chain; smart00030"
                /db_xref="CDD:128345"
Region          29..447
                /region_name="Clusterin"
                /note="Clusterin; pfam01093"
                /db_xref="CDD:189838"
Region          228..443
                /region_name="CLa"
                /note="CLUSTERIN alpha chain; smart00035"
                /db_xref="CDD:128350"
CDS             1..447
                /gene="CLU"
                /coded_by="join(AY341244.1:1915..2011,
                AY341244.1:3400..3548,AY341244.1:5962..6132,
                AY341244.1:7151..7562,AY341244.1:8091..8195,
                AY341244.1:12480..12709,AY341244.1:13852..14031)"
```

Mascot: http://www.matrixscience.com/

Spot 7

Clusterin

MASCOT Search Results

Protein View: gi|32891795

clusterin (complement lysis inhibitor, SP-40,40, sulfated glycoprotein 2, testosterone-repressed prostate message 2, apolipoprotein J) [Homo sapiens]

**Database:**	NCBInr
**Score:**	97
**Nominal mass (M_r):**	52203
**Calculated pI:**	6.06
**Taxonomy:**	Homo sapiens

Sequence similarity is available as an NCBI BLAST search of gi|32891795 against nr.

Search parameters

**MS data file:**	Spot-7-060316 CYWu-9.pkl
**Enzyme:**	Trypsin: cuts C-term side of KR unless next residue is P.
**Variable modifications:**	Carbamidomethyl (C), Oxidation (M)

Protein sequence coverage: 6%

Matched peptides shown in ***bold red***.

```
  1 MMKTLLLFVG LLLTWESGQV LGDQTVSDNE LQEMSNQGSK YVNKEIQNAV
 51 NGVKQIKTLI EKTNEERKTL LSNLEEAKKK KEDALNETRE SETKLKELPG
101 VCNETMMALW EECKPCLKQT CMKFYARVCR SGSGLVGRQL EEFLNQSSPF
151 YFWMNGDRID SLLENDRQQT HMLDVMQDHF SRASSIIDEL FQDRFFTREP
201 QDTYHYLPFS LPHRRPHFFF PKSRIVRSLM PFSPYEPLNF HAMFQPFLEM
251 IHEAQQAMDI HFHSPAFQHP PTEFIREGDD DRTVCREIRH NSTGCLRMKD
301 QCDKCREILS VDCSTNNPSQ AKLRRELDES LQVAERLTRK YNELLKSYQW
351 KMLNTSSLLE QLNEQFNWVS RLANLTQGED QYYLRVTTVA SHTSDSDVPS
401 GVTEVVVKLF DSDPITVTVP VEVSRKNPKF METVAEKALQ EYRKKHR
```

Unformatted sequence string: 447 residues (for pasting into other applications).

Sort peptides by ◉ Residue Number ○ Increasing Mass ○ Decreasing Mass

Show predicted peptides also

Query	Start – End	Observed	Mr(expt)	Mr(calc)	Delta	M	Score	Expect	Rank	U	Peptide
9	69 – 78	559.3799	1116.7452	1116.6026	0.1426	0	15	31	4	U	K.TLLSNLEEAK.K
10	183 – 194	697.4243	1392.8340	1392.6885	0.1456	0	30	0.85	1	U	R.ASSIIDELFQDR.F
6	430 – 437	485.7848	969.5550	969.4477	0.1073	0	54	0.0028	1	U	K.FMETVAEK.A + Oxidation (M)

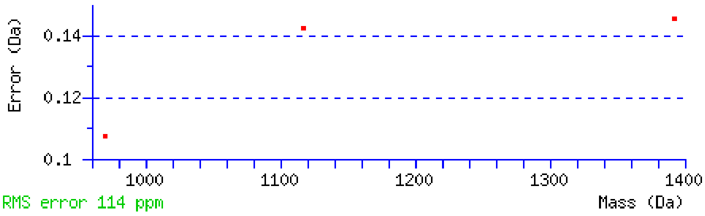

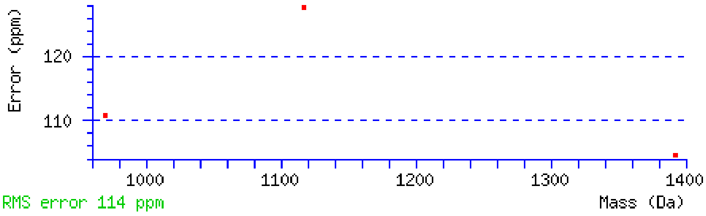

```
LOCUS       AAP88927                 447 aa            linear   PRI 22-JUL-2003
DEFINITION  clusterin (complement lysis inhibitor, SP-40,40, sulfated
            glycoprotein 2, testosterone-repressed prostate message 2,
            apolipoprotein J) [Homo sapiens].
ACCESSION   AAP88927
VERSION     AAP88927.1  GI:32891795
DBSOURCE    accession AY341244.1
KEYWORDS    .
SOURCE      Homo sapiens (human)
  ORGANISM  Homo sapiens
            Eukaryota; Metazoa; Chordata; Craniata; Vertebrata; Euteleostomi;
            Mammalia; Eutheria; Euarchontoglires; Primates; Haplorrhini;
            Catarrhini; Hominidae; Homo.
REFERENCE   1  (residues 1 to 447)
  AUTHORS   Rieder,M.J., Livingston,R.J., Daniels,M.R., Chung,M.-W.,
            Miyamoto,K.E., Nguyen,C.P., Nguyen,D.A., Poel,C.L., Robertson,P.D.,
            Schackwitz,W.S., Sherwood,J.K., Witrak,L.A. and Nickerson,D.A.
  TITLE     Direct Submission
  JOURNAL   Submitted (11-JUL-2003) Genome Sciences, University of Washington,
            1705 NE Pacific, Seattle, WA 98195, USA
COMMENT     Method: conceptual translation supplied by author.
FEATURES             Location/Qualifiers
     source          1..447
```

```
Protein         1..447
                /product="clusterin (complement lysis inhibitor, SP-40,40,
                sulfated glycoprotein 2, testosterone-repressed prostate
                message 2, apolipoprotein J)"
Region          23..227
                /region_name="CLb"
                /note="CLUSTERIN Beta chain; smart00030"
                /db_xref="CDD:128345"
Region          29..447
                /region_name="Clusterin"
                /note="Clusterin; pfam01093"
                /db_xref="CDD:189838"
Region          228..443
                /region_name="CLa"
                /note="CLUSTERIN alpha chain; smart00035"
                /db_xref="CDD:128350"
CDS             1..447
                /gene="CLU"
                /coded_by="join(AY341244.1:1915..2011,
                AY341244.1:3400..3548,AY341244.1:5962..6132,
                AY341244.1:7151..7562,AY341244.1:8091..8195,
                AY341244.1:12480..12709,AY341244.1:13852..14031)"
```

Spot 8

Clusterin

MATRIX SCIENCE MASCOT Search Results

Protein View: gi|32891795

clusterin (complement lysis inhibitor, SP-40,40, sulfated glycoprotein 2, testosterone-repressed prostate message 2, apolipoprotein J) [Homo sapiens]

**Database:**	NCBInr
**Score:**	59
**Nominal mass (M_r):**	52203
**Calculated pI:**	6.06
**Taxonomy:**	~~Homo sapiens~~

Sequence similarity is available as ~~an NCBI BLAST search of gi|32891795 against nr~~.

Search parameters

**MS data file:**	Spot-8-060316 CYWu-10.pkl
**Enzyme:**	Trypsin: cuts C-term side of KR unless next residue is P.
**Variable modifications:**	~~Carbamidomethyl (C)~~, ~~Oxidation (M)~~

Protein sequence coverage: 4%

Matched peptides shown in ***bold red***.

```
  1 MMKTLLLFVG LLLTWESGQV LGDQTVSDNE LQEMSNQGSK YVNKEIQNAV
 51 NGVKQIKTLI EKTNEERKTL LSNLEEAKKK KEDALNETRE SETKLKELPG
101 VCNETMMALW EECKPCLKQT CMKFYARVCR SGSGLVGRQL EEFLNQSSPF
151 YFWMNGDRID SLLENDRQQT HMLDVMQDHF SRASSIIDEL FQDRFFTREP
201 QDTYHYLPFS LPHRRPHFFF PKSRIVRSLM PFSPYEPLNF HAMFQPFLEM
251 IHEAQQAMDI HFHSPAFQHP PTEFIREGDD DRTVCREIRH NSTGCLRMKD
301 QCDKCREILS VDCSTNNPSQ AKLRRELDES LQVAERLTRK YNELLKSYQW
351 KMLNTSSLLE QLNEQFNWVS RLANLTQGED QYYLRVTTVA SHTSDSDVPS
401 GVTEVVVKLF DSDPITVTVP VEVSRKNPKF METVAEKALQ EYRKKHR
```

Unformatted sequence string: ~~447 residues~~ (for pasting into other applications).

Sort peptides by ◉ Residue Number ○ Increasing Mass ○ Decreasing Mass

Show predicted peptides also

Query	Start – End	Observed	Mr(expt)	Mr(calc)	Delta	M	Score	Expect	Rank	U	Peptide
14	326 – 336	644.8116	1287.6086	1287.6306	-0.0220	0	35	0.28	1	U	R.ELDESLQVAER.L
10	430 – 437	485.7460	969.4774	969.4477	0.0297	0	24	3	1	U	K.FMETVAEK.A + Oxidation (M)

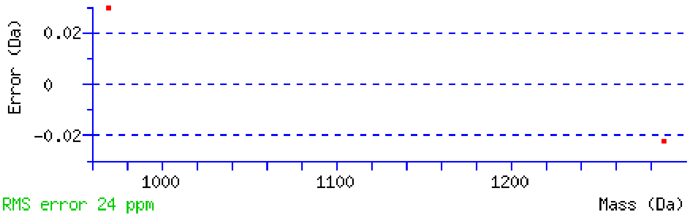

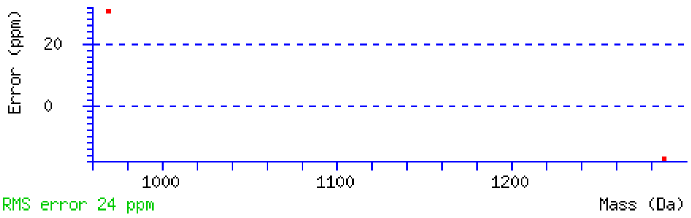


```
LOCUS       AAP88927                 447 aa            linear   PRI 22-JUL-2003
DEFINITION  clusterin (complement lysis inhibitor, SP-40,40, sulfated
            glycoprotein 2, testosterone-repressed prostate message 2,
            apolipoprotein J) [Homo sapiens].
ACCESSION   AAP88927
VERSION     AAP88927.1  GI:32891795
DBSOURCE    accession AY341244.1
KEYWORDS    .
SOURCE      Homo sapiens (human)
  ORGANISM  Homo sapiens
            Eukaryota; Metazoa; Chordata; Craniata; Vertebrata; Euteleostomi;
            Mammalia; Eutheria; Euarchontoglires; Primates; Haplorrhini;
            Catarrhini; Hominidae; Homo.
REFERENCE   1  (residues 1 to 447)
  AUTHORS   Rieder,M.J., Livingston,R.J., Daniels,M.R., Chung,M.-W.,
            Miyamoto,K.E., Nguyen,C.P., Nguyen,D.A., Poel,C.L., Robertson,P.D.,
            Schackwitz,W.S., Sherwood,J.K., Witrak,L.A. and Nickerson,D.A.
  TITLE     Direct Submission
  JOURNAL   Submitted (11-JUL-2003) Genome Sciences, University of Washington,
            1705 NE Pacific, Seattle, WA 98195, USA
COMMENT     Method: conceptual translation supplied by author.
FEATURES             Location/Qualifiers
     source          1..447
                     /organism="Homo sapiens"
```

```
                     sulfated glycoprotein 2, testosterone-repressed prostate
                     message 2, apolipoprotein J)"
     Region          23..227
                     /region_name="CLb"
                     /note="CLUSTERIN Beta chain; smart00030"
                     /db_xref="CDD:128345"
     Region          29..447
                     /region_name="Clusterin"
                     /note="Clusterin; pfam01093"
                     /db_xref="CDD:189838"
     Region          228..443
                     /region_name="CLa"
                     /note="CLUSTERIN alpha chain; smart00035"
                     /db_xref="CDD:128350"
     CDS             1..447
                     /gene="CLU"
                     /coded_by="join(AY341244.1:1915..2011,
                     AY341244.1:3400..3548,AY341244.1:5962..6132,
                     AY341244.1:7151..7562,AY341244.1:8091..8195,
                     AY341244.1:12480..12709,AY341244.1:13852..14031)"
```

Spot 9

Complement factor H

MASCOT Search Results

Protein View: gi|758073

complement factor H [Homo sapiens]

**Database:**	NCBInr
**Score:**	180
**Nominal mass (M_r):**	51000
**Calculated pI:**	6.77
**Taxonomy:**	Homo sapiens

This protein sequence matches the following other entries:

- gi|22713406 from Homo sapiens
- gi|83405578 from Homo sapiens
- gi|325463197 from synthetic construct

Sequence similarity is available as an NCBI BLAST search of gi|758073 against nr.

Search parameters

**MS data file:**	Spot-9-050815 Plasma-C3.pkl
**Enzyme:**	Trypsin: cuts C-term side of KR unless next residue is P.
**Variable modifications:**	Carbamidomethyl (C), Oxidation (M)

Protein sequence coverage: 10%

Matched peptides shown in ***bold red***.

```
  1 MRLLAKIICL MLWAICVAED CNELPPRRNT EILTGSWSDQ TYPEGTQAIY
 51 KCRPGYRSLG NVIMVCRKGE WVALNPLRKC QKRPCGHPGD TPFGTFTLTG
101 GNVFEYGVKA VYTCNEGYQL LGEINYRECD TDGWTNDIPI CEVVKCLPVT
151 APENGKIVSS AMEPDREYHF GQAVRFVCNS GYKIEGDEEM HCSDDGFWSK
201 EKPKCVEISC KSPDVINGSP ISQKIIYKEN ERFQYKCNMG YEYSERGDAV
251 CTESGWRPLP SCEEKSCDNP YIPNGDYSPL RIKHRTGDEI TYQCRNGFYP
301 ATRGNTAKCT STGWIPAPRC TLKPCDYPDI KHGGLYHENM RRPYFPVAVG
351 KYYSYYCDEH FETPSGSYWD HIHCTQDGWS PAVPCLRKCY FPYLENGYNQ
401 NYGRKFVQGK SIDVACHPGY ALPKAQTTVT CMENGWSPTP RCIRVSFTL
```

Unformatted sequence string: 449 residues (for pasting into other applications).

Sort peptides by ◉ Residue Number ○ Increasing Mass ○ Decreasing Mass

Show predicted peptides also

Query	Start – End	Observed	Mr(expt)	Mr(calc)	Delta	M	Score	Expect	Rank	U	Peptide
7	58 – 67	582.9553	1163.8960	1163.5791	0.3169	0	25	3.1	1	U	R.SLGNVIMVCR.K + Carbamidomethyl (C); Oxidation (M)
9	146 – 156	593.4587	1184.9028	1184.5860	0.3169	0	41	0.08	1	U	K.CLPVTAPENGK.I + Carbamidomethyl (C)
11	212 – 224	671.5208	1341.0270	1340.6936	0.3335	0	65	0.00026	1	U	K.SPDVINGSPISQK.I
10	309 – 319	623.4694	1244.9242	1244.5972	0.3271	0	51	0.0077	1	U	K.CTSTGWIPAPR.C + Carbamidomethyl (C)

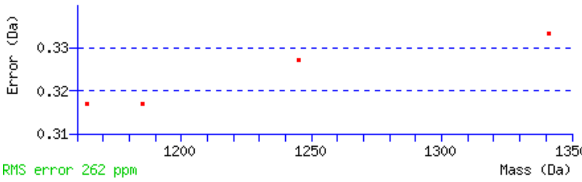

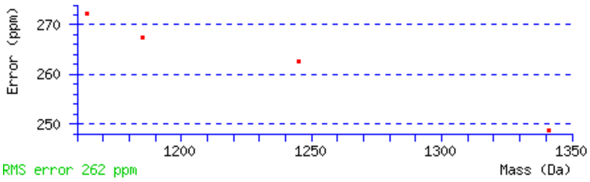

```
LOCUS       CAA30403                 449 aa            linear   PRI 07-OCT-2008
DEFINITION  complement factor H [Homo sapiens].
ACCESSION   CAA30403
VERSION     CAA30403.1  GI:758073
DBSOURCE    embl accession X07523.1
KEYWORDS    .
SOURCE      Homo sapiens (human)
  ORGANISM  Homo sapiens
            Eukaryota; Metazoa; Chordata; Craniata; Vertebrata; Euteleostomi;
            Mammalia; Eutheria; Euarchontoglires; Primates; Haplorrhini;
            Catarrhini; Hominidae; Homo.
REFERENCE   1
  AUTHORS   Ripoche,J., Day,A.J., Harris,T.J. and Sim,R.B.
  TITLE     The complete amino acid sequence of human complement factor H
  JOURNAL   Biochem. J. 249 (2), 593-602 (1988)
   PUBMED   2963625
REFERENCE   2  (residues 1 to 449)
  AUTHORS   Day,A.J.
  TITLE     Direct Submission
  JOURNAL   Submitted (03-MAY-1988) Day A.J., Dept. of Biochemistry, University
            of Oxford, MRC Immunochemistry Unit, South Parks Road, Oxford OX1
            3QU
COMMENT     Data kindly reviewed (06-JUN-1988) by Day A.J.
FEATURES             Location/Qualifiers
     source          1..449
                     /organism="Homo sapiens"
                     /db_xref="taxon:9606"
                     /clone="B-38-1 tissue=liver"
                     /clone_lib="pat153/PvuII/8"
     Protein         1..449
                     /product="complement factor H"
     sig_peptide     1..18
                     /note="put. signal peptide"
     mat_peptide     19..449
                     /product="translated region"
```

```
                     identified in several proteins of the complement system;
                     cl00043"
                     /db_xref="CDD:206798"
     Region          85..142
                     /region_name="CCP"
                     /note="Complement control protein (CCP) modules (aka short
                     consensus repeats SCRs or SUSHI repeats) have been
                     identified in several proteins of the complement system;
                     cd00033"
                     /db_xref="CDD:153056"
     Site            order(95,115)
                     /site_type="other"
                     /note="receptor-ligand interactions"
                     /db_xref="CDD:153056"
     Region          146..206
                     /region_name="CCP"
                     /note="Complement control protein (CCP) modules (aka short
                     consensus repeats SCRs or SUSHI repeats) have been
                     identified in several proteins of the complement system;
                     cd00033"
                     /db_xref="CDD:153056"
     Site            order(156,179)
                     /site_type="other"
                     /note="receptor-ligand interactions"
                     /db_xref="CDD:153056"
     Region          210..263
                     /region_name="CCP"
                     /note="Complement control protein (CCP) modules (aka short
                     consensus repeats SCRs or SUSHI repeats) have been
                     identified in several proteins of the complement system;
                     cd00033"
                     /db_xref="CDD:153056"
     Site            order(219,238)
                     /site_type="other"
                     /note="receptor-ligand interactions"
                     /db_xref="CDD:153056"
     Region          267..320
                     /region_name="CCP"
                     /note="Complement control protein (CCP) modules (aka short
                     consensus repeats SCRs or SUSHI repeats) have been
                     identified in several proteins of the complement system;
                     cl00043"
                     /db_xref="CDD:206798"
     Region          320..>449
                     /region_name="PHA02817"
                     /note="EEV Host range protein; Provisional"
                     /db_xref="CDD:165167"
     Region          389..443
                     /region_name="CCP"
                     /note="Complement control protein (CCP) modules (aka short
                     consensus repeats SCRs or SUSHI repeats) have been
                     identified in several proteins of the complement system;
                     cd00033"
                     /db_xref="CDD:153056"
     Site            order(398,417)
                     /site_type="other"
                     /note="receptor-ligand interactions"
                     /db_xref="CDD:153056"
     CDS             1..449
                     /coded_by="X07523.1:74..1423"
                     /db_xref="GDB:120041"
                     /db_xref="GOA:P08603"
                     /db_xref="HGNC:4883"
                     /db_xref="InterPro:IPR000436"
                     /db_xref="InterPro:IPR016060"
                     /db_xref="PDB:1FHC"
                     /db_xref="PDB:1HAQ"
                     /db_xref="PDB:1HCC"
                     /db_xref="PDB:1HFH"
                     /db_xref="PDB:1HFI"
                     /db_xref="PDB:1KOV"
                     /db_xref="PDB:2BZM"
                     /db_xref="PDB:2G7I"
                     /db_xref="PDB:2IC4"
                     /db_xref="PDB:2JGW"
                     /db_xref="PDB:2JGX"
                     /db_xref="PDB:2KMS"
                     /db_xref="PDB:2QFG"
                     /db_xref="PDB:2QFH"
                     /db_xref="PDB:2RLP"
                     /db_xref="PDB:2RLQ"
                     /db_xref="PDB:2UWN"
                     /db_xref="PDB:2V8E"
                     /db_xref="PDB:2W80"
                     /db_xref="PDB:2W81"
                     /db_xref="PDB:2WII"
                     /db_xref="PDB:3GAU"
                     /db_xref="PDB:3GAV"
                     /db_xref="PDB:3GAW"
                     /db_xref="PDB:3KXV"
                     /db_xref="PDB:3KZJ"
                     /db_xref="UniProtKB/Swiss-Prot:P08603"
```

Spot 10

Complement factor B

MASCOT Search Results

Protein View: gi|291922

complement factor B [Homo sapiens]

**Database:**	NCBInr
**Score:**	89
**Nominal mass (M_r):**	85450
**Calculated pI:**	6.55
**Taxonomy:**	~~Homo sapiens~~

This protein sequence matches the following other entries:

- ~~gi|2347133~~ from ~~Homo sapiens~~

Sequence similarity is available as ~~an NCBI BLAST search of gi|291922 against nr~~.

Search parameters

**MS data file:**	Spot-10-060125 CYWu B-6.pkl
**Enzyme:**	Trypsin: cuts C-term side of KR unless next residue is P.
**Variable modifications:**	~~Carbamidomethyl (C)~~, ~~Oxidation (M)~~

Protein sequence coverage: 2%

Matched peptides shown in ***bold red***.

1 MGSNLSPQLC LMPFILGLLS GGVTTTPWSL AQPQGSCSLE GVEIKGGSFR
51 LLQEGQALEY VCPSGFYPYP VQTRTCRSTG SWSTLKTQDQ KTVRKAECRA
101 IHCPRPHDFE NGEYWPRSPY YNVSDEISFH CYDGYTLRGS ANRTCQVNGR
151 WSGQTAICDN GAGYCSNPGI PIGTRKVGSQ YRLEDSVTYH CSRGLTLRGS
201 QRRTCQEGGS WSGTEPSCQD SFMYDTPQEV AEAFLSSLTE TIEGVDAEDG
251 HGPGEQQKRK IVLDPSGSMN IYLVLDGSDS IGASNFTGAK KCLVNLIEKV
301 ASYGVKPR**YG** **LVTYATYPK**I WVKVSEADSS NADWVTKQLN EINYEDHKLK
351 SGTNTKKALQ AVYSMMSWPD DVPPEGWNRT RHVIILMTDG LHNMGGDPIT
401 VIDEIRDLLY IGKDRKNPRE DYLDVYVFGV GPLVNQVNIN ALASKKDNEQ
451 HVFKVKDMEN LEDVFYQMID ESQSLSLCGM VWEHRKGTDY HKQPWQAKIS
501 VIRPSKGHES CMGAVVSEYF VLTAAHCFTV DDKEHSIKVS VGGEKRDLEI
551 EVVLFHPNYN INGKKEAGIP EFYDYDVALI KLKNKLKYGQ TIRPICLPCT
601 EGTTRALRLP PTTTCQQQK**E** **ELLPAQDIK**A LFVSEEEKKL TRKEVYIKNG
651 DKKGSCERDA QYAPGYDKVK DISEVVTPRF LCTGGVSPYA DPNTCRGDSG
701 GPLIVHKRSR FIQVGVISWG VVDVCKNQKR QKQVPAHARD FHINLFQVLP
751 WLKEKLQDED LGFL

Unformatted sequence string: ~~764 residues~~ (for pasting into other applications).

Sort peptides by ◉ Residue Number ○ Increasing Mass ○ Decreasing Mass

Show predicted peptides also

Query	Start – End	Observed	Mr(expt)	Mr(calc)	Delta	M	Score	Expect	Rank	U	Peptide
7	309 – 319	638.2852	1274.5558	1274.6547	-0.0988	0	58	0.0013	1	U	R.YGLVTYATYPK.I
6	620 – 629	578.2794	1154.5442	1154.6183	-0.0740	0	31	0.71	1	U	K.EELLPAQDIK.A

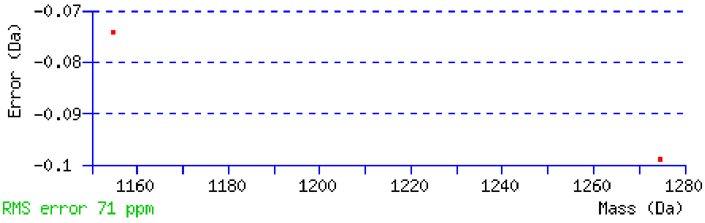

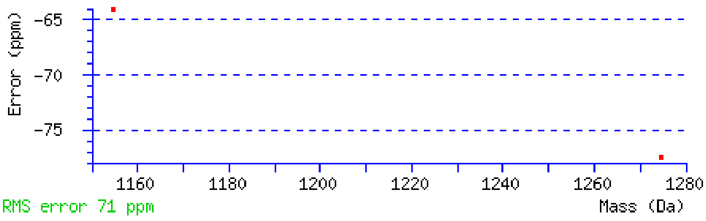


```
LOCUS       AAA16820                 764 aa            linear   PRI 16-MAR-1994
DEFINITION  complement factor B [Homo sapiens].
ACCESSION   AAA16820
VERSION     AAA16820.1  GI:291922
DBSOURCE    locus HUMCOMFACB accession L15702.1
KEYWORDS    .
SOURCE      Homo sapiens (human)
  ORGANISM  Homo sapiens
            Eukaryota; Metazoa; Chordata; Craniata; Vertebrata; Euteleostomi;
            Mammalia; Eutheria; Euarchontoglires; Primates; Haplorrhini;
            Catarrhini; Hominidae; Homo.
REFERENCE   1  (residues 1 to 764)
```

```
            phenotypic conversion by site-directed mutagenesis and expression
  JOURNAL   Mol. Immunol. 30 (17), 1587-1592 (1993)
   PUBMED   8247029
COMMENT     Method: conceptual translation.
FEATURES             Location/Qualifiers
     source          1..764
                     /organism="Homo sapiens"
                     /db_xref="taxon:9606"
     Protein         1..764
                     /product="complement factor B"
     sig_peptide     1..25
     Region          <55..83
                     /region_name="CCP"
                     /note="Complement control protein (CCP) modules (aka short
                     consensus repeats SCRs or SUSHI repeats) have been
                     identified in several proteins of the complement system;
                     cl00043"
                     /db_xref="CDD:206798"
     Region          103..158
                     /region_name="CCP"
                     /note="Complement control protein (CCP) modules (aka short
                     consensus repeats SCRs or SUSHI repeats) have been
                     identified in several proteins of the complement system;
                     cd00033"
                     /db_xref="CDD:153056"
     Site            order(113,132)
                     /site_type="other"
                     /note="receptor-ligand interactions"
                     /db_xref="CDD:153056"
     Region          165..219
                     /region_name="CCP"
                     /note="Complement control protein (CCP) modules (aka short
                     consensus repeats SCRs or SUSHI repeats) have been
                     identified in several proteins of the complement system;
                     cd00033"
                     /db_xref="CDD:153056"
     Site            order(175,192)
                     /site_type="other"
                     /note="receptor-ligand interactions"
                     /db_xref="CDD:153056"
     Region          269..466
                     /region_name="vWA_complement_factors"
                     /note="Complement factors B and C2 are two critical
                     proteases for complement activation. They both contain
                     three CCP or Sushi domains, a trypsin-type serine protease
                     domain and a single VWA domain with a conserved metal ion
                     dependent adhesion site referred...; cd01470"
                     /db_xref="CDD:29243"
     Site            order(276,278,280,353,389)
                     /site_type="other"
                     /note="metal ion-dependent adhesion site (MIDAS)"
                     /db_xref="CDD:29243"
     Region          488..755
                     /region_name="Tryp_SPc"
                     /note="Trypsin-like serine protease; Many of these are
                     synthesized as inactive precursor zymogens that are
                     cleaved during limited proteolysis to generate their
                     active forms. Alignment contains also inactive enzymes
                     that have substitutions of the catalytic triad...;
                     cd00190"
                     /db_xref="CDD:29152"
     Site            order(526,576,699)
                     /site_type="active"
                     /db_xref="CDD:29152"
     Site            order(693,718,720)
                     /site_type="other"
                     /note="substrate binding sites [chemical binding]"
                     /db_xref="CDD:29152"
     CDS             1..764
                     /coded_by="L15702.1:41..2335"
```

Mascot: http://www.matrixscience.com/

Spot 11

α1-antitrypsin

MASCOT Search Results

Protein View: gi|157831596

Chain A, Alpha1-Antitrypsin

**Database:**	NCBInr
**Score:**	87
**Expect:**	0.00052
**Nominal mass (M_r):**	44223
**Calculated pI:**	5.37
**Taxonomy:**	Homo sapiens

Sequence similarity is available as an NCBI BLAST search of gi|157831596 against nr.

Search parameters

**MS data file:**	peaklist.xml
**Enzyme:**	Trypsin: cuts C-term side of KR unless next residue is P.
**Variable modifications:**	Carbamidomethyl (C), Oxidation (M)
**Mass values searched:**	7
**Mass values matched:**	6

Protein sequence coverage: 21%

Matched peptides shown in ***bold red***.

1 EDPQGDAAQK **TDTSHHDQDH** **PTFNKITPNL** **AEFAFSLYR**Q LAHQSNSTNI
51 FFSPVSIAAA FAMLSLGAKG DTHDEILEGL NFNLTEIPEA QIHEGFQELL
101 RTLNQPDSQL QLTTGNGLFL SEGLKLVDKF LEDVKK**LYHS** **EAFTVNFGDT**
151 **EEAKK**QINDY VEKGTQGKIV DLVKELDRDT VFALVNYIFF K**GKWERPFEV**
201 **K**DTEEEDFHV DQVTTVKVPM MKRLGMFNIQ HCKKLSSWVL LMKYLGNATA
251 IFFLPDEGKL QHLENELTHD IITK**FLENED** **RR**SASLHLPK LSITGTYDLK
301 SVLGQLGITK **VFSNGADLSG** **VTEEAPLK**LS KAVHKAVLTI DEKGTEAAGA
351 MFLEAIPMSI PPEVKFNKPF VFLMIEQNTK SPLFMGKVVN PTQK

Unformatted sequence string: 394 residues (for pasting into other applications).

Sort peptides by ◉ Residue Number ○ Increasing Mass ○ Decreasing Mass

Show predicted peptides also

Start – End	Observed	Mr(expt)	Mr(calc)	ppm	M	Peptide
11 – 25	1779.5665	1778.5593	1778.7609	-113	0	K.TDTSHHDQDHPTFNK.I
26 – 39	1641.7728	1640.7656	1640.8562	-55.3	0	K.ITPNLAEFAFSLYR.Q
137 – 155	2185.8008	2184.7935	2185.0327	-109	1	K.LYHSEAFTVNFGDTEEAKK.Q
192 – 201	1275.5505	1274.5433	1274.6771	-105	1	K.GKWERPFEVK.D
275 – 282	1078.4049	1077.3976	1077.5203	-114	1	K.FLENEDRR.S
311 – 328	1833.7111	1832.7038	1832.9156	-116	0	K.VFSNGADLSGVTEEAPLK.L

No match to: 1307.5281

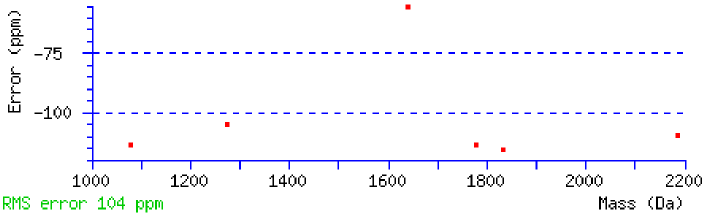

```
LOCUS       1KCT_A                   394 aa            linear   PRI 10-JUL-2009
DEFINITION  Chain A, Alpha1-Antitrypsin.
ACCESSION   1KCT_A
VERSION     1KCT_A  GI:157831596
DBSOURCE    pdb: molecule 1KCT, chain 65, release Jun 18, 2009;
            deposition: Aug 6, 1996;
            class: Serine Protease Inhibitor;
            source: Mol_id: 1; Organism_scientific: Homo Sapiens;
            Organism_common: Human; Organism_taxid: 9606; Gene: A1at;
            Expression_system: Escherichia Coli Bl21(De3);
            Expression_system_taxid: 469008; Expression_system_strain:
            Bl21(De3); Expression_system_plasmid: Pet-8c;
            Expression_system_gene: A1at;
            Exp. method: X-Ray Diffraction.
KEYWORDS    .
SOURCE      Homo sapiens (human)
```

```
            Catarrhini; Hominidae; Homo.
REFERENCE   1  (residues 1 to 394)
  AUTHORS   Song,H.K., Lee,K.N., Kwon,K.S., Yu,M.H. and Suh,S.W.
  TITLE     Crystal structure of an uncleaved alpha 1-antitrypsin reveals the
            conformation of its inhibitory reactive loop
  JOURNAL   FEBS Lett. 377 (2), 150-154 (1995)
   PUBMED   8543039
REFERENCE   2  (residues 1 to 394)
  AUTHORS   Song,H.K. and Suh,S.W.
  TITLE     Direct Submission
  JOURNAL   Submitted (06-AUG-1996)
COMMENT     SEQRES.
FEATURES             Location/Qualifiers
     source          1..394
                     /organism="Homo sapiens"
                     /db_xref="taxon:9606"
     Region          24..391
                     /region_name="PHA02660"
                     /note="serpin-like protein; Provisional"
                     /db_xref="CDD:165039"
     SecStr          27..44
                     /sec_str_type="helix"
                     /note="helix 1"
     Region          31..388
                     /region_name="alpha-1-antitrypsin_like"
                     /note="alpha-1-antitrypsin_like. This family contains a
                     variety of different members of clade A of the serpin
                     superfamily. They include the classical serine proteinase
                     inhibitors, alpha-1-antitrypsin and
                     alpha-1-antichymotrypsin, protein C inhibitor; cd02056"
                     /db_xref="CDD:29131"
     SecStr          49..54
                     /sec_str_type="sheet"
                     /note="strand 1"
     SecStr          55..63
                     /sec_str_type="helix"
                     /note="helix 2"
     SecStr          94..101
                     /sec_str_type="helix"
                     /note="helix 3"
     SecStr          110..121
                     /sec_str_type="sheet"
                     /note="strand 2"
     SecStr          128..137
                     /sec_str_type="helix"
                     /note="helix 4"
     SecStr          157..164
                     /sec_str_type="helix"
                     /note="helix 5"
     SecStr          181..192
                     /sec_str_type="sheet"
                     /note="strand 3"
     SecStr          193..196
                     /sec_str_type="sheet"
                     /note="strand 4"
     SecStr          207..211
                     /sec_str_type="sheet"
                     /note="strand 5"
     SecStr          213..217
                     /sec_str_type="sheet"
                     /note="strand 6"
     SecStr          219..224
                     /sec_str_type="sheet"
                     /note="strand 7"
     SecStr          225..233
                     /sec_str_type="sheet"
                     /note="strand 8"
     SecStr          235..244
                     /sec_str_type="sheet"
                     /note="strand 9"
     SecStr          247..256
                     /sec_str_type="sheet"
                     /note="strand 10"
     SecStr          280..283
                     /sec_str_type="sheet"
                     /note="strand 11"
     SecStr          284..290
                     /sec_str_type="sheet"
                     /note="strand 12"
     SecStr          293..300
                     /sec_str_type="sheet"
                     /note="strand 13"
     SecStr          330..338
                     /sec_str_type="sheet"
                     /note="strand 14"
     SecStr          343..346
                     /sec_str_type="sheet"
                     /note="strand 15"
     Site            order(344..354,364..369)
                     /site_type="other"
                     /note="reactive center loop"
                     /db_xref="CDD:29131"
```

```
SecStr          369..377
                /sec_str_type="sheet"
                /note="strand 17"
SecStr          379..389
                /sec_str_type="sheet"
                /note="strand 18"
```

Spot 12

α1-antitrypsin

MASCOT Search Results

Protein View: gi|157831596

Chain A, Alpha1-Antitrypsin

**Database:**	NCBInr
**Score:**	96
**Expect:**	6e-05
**Nominal mass (M_r):**	44223
**Calculated pI:**	5.37
**Taxonomy:**	Homo sapiens

Sequence similarity is available as an NCBI BLAST search of gi|157831596 against nr.

Search parameters

**MS data file:**	peaklist.xml
**Enzyme:**	Trypsin: cuts C-term side of KR unless next residue is P.
**Variable modifications:**	Carbamidomethyl (C), Oxidation (M)
**Mass values searched:**	13
**Mass values matched:**	8

Protein sequence coverage: 28%

Matched peptides shown in ***bold red***.

1 EDPQGDAAQK **TDTSHHDQDH PTFNK**ITPNL AEFAFSLYRQ LAHQSNSTNI
51 FFSPVSIAAA FAMLSLGAKG DTHDEILEGL NFNLTEIPEA QIHEGFQELL
101 RTLNQPDSQL QLTTGNGLFL SEGLKLVDKF LEDVKK**LYHS EAFTVNFGDT**
151 **EEAKK**QINDY VEKGTQGKIV DLVKELDRDT VFALVNYIFF K**GKWERPFEV**
201 **KDTEEEDFHV DQVTTVK**VPM MKR**LGMFNIQ HCK**KLSSWVL LMKYLGNATA
251 IFFLPDEGK**L QHLENELTHD IITKFLENED RR**SASLHLPK LSITGTYDLK
301 SVLGQLGITK **VFSNGADLSG VTEEAPLK**LS KAVHKAVLTI DEKGTEAAGA
351 MFLEAIPMSI PPEVKFNKPF VFLMIEQNTK SPLFMGKVVN PTQK

Unformatted sequence string: 394 residues (for pasting into other applications).

Sort peptides by ◉ Residue Number ○ Increasing Mass ○ Decreasing Mass

Show predicted peptides also

Start – End	Observed	Mr(expt)	Mr(calc)	ppm	M	Peptide
11 – 25	1779.7234	1778.7161	1778.7609	-25.2	0	K.TDTSHHDQDHPTFNK.I
137 – 155	2185.9575	2184.9502	2185.0327	-37.8	1	K.LYHSEAFTVNFGDTEEAKK.Q
192 – 201	1275.6504	1274.6431	1274.6771	-26.7	1	K.GKWERPFEVK.D
202 – 217	1891.8049	1890.7977	1890.8483	-26.8	0	K.DTEEEDFHVDQVTTVK.V
224 – 233	1263.5431	1262.5358	1262.5900	-42.9	0	R.LGMFNIQHCK.K + Carbamidomethyl (C); Oxidation (M)
260 – 274	1803.9180	1802.9107	1802.9526	-23.3	0	K.LQHLENELTHDIITK.F
275 – 282	1078.4764	1077.4692	1077.5203	-47.5	1	K.FLENEDRR.S
311 – 328	1833.8790	1832.8718	1832.9156	-23.9	0	K.VFSNGADLSGVTEEAPLK.L

No match to: 1046.4745, 1063.1987, 1307.6335, 1333.7429, 2211.0051

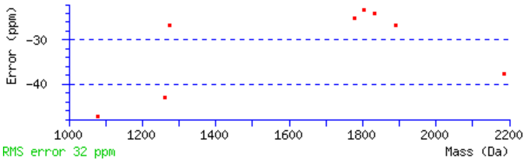

```
LOCUS       1KCT_A                  394 aa            linear   PRI 10-JUL-2009
DEFINITION  Chain A, Alpha1-Antitrypsin.
ACCESSION   1KCT_A
VERSION     1KCT_A  GI:157831596
DBSOURCE    pdb: molecule 1KCT, chain 65, release Jun 18, 2009;
            deposition: Aug 6, 1996;
            class: Serine Protease Inhibitor;
            source: Mol_id: 1; Organism_scientific: Homo Sapiens;
            Organism_common: Human; Organism_taxid: 9606; Gene: A1at;
            Expression_system: Escherichia Coli Bl21(De3);
            Expression_system_taxid: 469008; Expression_system_strain:
            Bl21(De3); Expression system plasmid: Pet-8c;
```

```
            Expression_system_gene: A1at;
            Exp. method: X-Ray Diffraction.
KEYWORDS    .
SOURCE      Homo sapiens (human)
  ORGANISM  Homo sapiens
            Eukaryota; Metazoa; Chordata; Craniata; Vertebrata; Euteleostomi;
            Mammalia; Eutheria; Euarchontoglires; Primates; Haplorrhini;
            Catarrhini; Hominidae; Homo.
REFERENCE   1  (residues 1 to 394)
  AUTHORS   Song,H.K., Lee,K.N., Kwon,K.S., Yu,M.H. and Suh,S.W.
  TITLE     Crystal structure of an uncleaved alpha 1-antitrypsin reveals the
            conformation of its inhibitory reactive loop
  JOURNAL   FEBS Lett. 377 (2), 150-154 (1995)
   PUBMED   8543039
REFERENCE   2  (residues 1 to 394)
  AUTHORS   Song,H.K. and Suh,S.W.
  TITLE     Direct Submission
  JOURNAL   Submitted (06-AUG-1996)
COMMENT     SEQRES.
FEATURES             Location/Qualifiers
     source          1..394
                     /organism="Homo sapiens"
                     /db_xref="taxon:9606"
     Region          24..391
                     /region_name="PHA02660"
                     /note="serpin-like protein; Provisional"
                     /db_xref="CDD:165039"
     SecStr          27..44
                     /sec_str_type="helix"
                     /note="helix 1"
     Region          31..388
                     /region_name="alpha-1-antitrypsin_like"
                     /note="alpha-1-antitrypsin_like. This family contains a
                     variety of different members of clade A of the serpin
                     superfamily. They include the classical serine proteinase
                     inhibitors, alpha-1-antitrypsin and
                     alpha-1-antichymotrypsin, protein C inhibitor; cd02056"
                     /db_xref="CDD:29131"
     SecStr          49..54
                     /sec_str_type="sheet"
                     /note="strand 1"
     SecStr          55..63
                     /sec_str_type="helix"
                     /note="helix 2"
     SecStr          94..101
                     /sec_str_type="helix"
                     /note="helix 3"
     SecStr          110..121
                     /sec_str_type="sheet"
                     /note="strand 2"
     SecStr          128..137
                     /sec_str_type="helix"
                     /note="helix 4"
     SecStr          157..164
                     /sec_str_type="helix"
                     /note="helix 5"
     SecStr          181..192
                     /sec_str_type="sheet"
                     /note="strand 3"
     SecStr          193..196
                     /sec_str_type="sheet"
                     /note="strand 4"
     SecStr          207..211
                     /sec_str_type="sheet"
                     /note="strand 5"
     SecStr          213..217
                     /sec_str_type="sheet"
                     /note="strand 6"
     SecStr          219..224
                     /sec_str_type="sheet"
                     /note="strand 7"
     SecStr          225..233
                     /sec_str_type="sheet"
                     /note="strand 8"
     SecStr          235..244
                     /sec_str_type="sheet"
                     /note="strand 9"
     SecStr          247..256
                     /sec_str_type="sheet"
                     /note="strand 10"
     SecStr          280..283
                     /sec_str_type="sheet"
                     /note="strand 11"
     SecStr          284..290
                     /sec_str_type="sheet"
                     /note="strand 12"
     SecStr          293..300
                     /sec_str_type="sheet"
                     /note="strand 13"
     SecStr          330..338
```

```
                     /sec_str_type="sheet"
                     /note="strand 14"
     SecStr          343..346
                     /sec_str_type="sheet"
                     /note="strand 15"
     Site            order(344..354,364..369)
                     /site_type="other"
                     /note="reactive center loop"
                     /db_xref="CDD:29131"
     SecStr          362..366
                     /sec_str_type="sheet"
                     /note="strand 16"
     SecStr          369..377
                     /sec_str_type="sheet"
                     /note="strand 17"
     SecStr          379..389
                     /sec_str_type="sheet"
                     /note="strand 18"
```

Spot 13

Apolipoprotein A-IV

MASCOT Search Results

Protein View: gi|563320

apolipoprotein A-IV, partial [Homo sapiens]

**Database:**	NCBInr
**Score:**	130
**Expect:**	2.5e-08
**Nominal mass (M_r):**	28141
**Calculated pI:**	5.39
**Taxonomy:**	~~Homo sapiens~~

Sequence similarity is available as ~~an NCBI BLAST search of gi|563320 against nr~~.

Search parameters

**MS data file:**	peaklist.xml
**Enzyme:**	Trypsin: cuts C-term side of KR unless next residue is P.
**Variable modifications:**	~~Carbamidomethyl (C)~~, ~~Oxidation (M)~~
**Mass values searched:**	24
**Mass values matched:**	11

Protein sequence coverage: 41%

Matched peptides shown in ***bold red***.

```
  1 LEPYADQLRT QVNTQAEQLR RQLDPLAQRM ERVLRENADS LQASLRPHAD
 51 ELKAKIDQNV EELKGRLTPY ADEFKVKIDQ TVEELRRSLA PYAQDTQEKL
101 NHQLEGLTFQ MKKNAEELKA RISASAEELR QRLAPLAEDV RGNLKGNTEG
151 LQKSLAELGG HLDQQVEEFR RRVEPYGENF NKALVQQMEQ LRQKLGPHAG
201 DVEGHLSFLE KDLRDKVNSF FSTFKEKESQ DKTLSLPELE QQQE
```

Unformatted sequence string: ~~244 residues~~ (for pasting into other applications).

Sort peptides by ◉ Residue Number ○ Increasing Mass ○ Decreasing Mass

Show predicted peptides also

Start – End	Observed	Mr(expt)	Mr(calc)	ppm	M	Peptide
1 – 9	1104.5139	1103.5066	1103.5611	-49.3	0	-.LEPYADQLR.T
10 – 21	1443.7653	1442.7580	1442.7590	-0.69	1	R.TQVNTQAEQLRR.Q
33 – 53	2362.4224	2361.4151	2361.2400	74.1	1	R.VLRENADSLQASLRPHADELK.A
36 – 53	1994.0566	1993.0494	1992.9864	31.6	0	R.ENADSLQASLRPHADELK.A
154 – 170	1927.9871	1926.9798	1926.9435	18.8	0	K.SLAELGGHLDQQVEEFR.R
154 – 171	2084.1997	2083.1924	2083.0446	70.9	1	K.SLAELGGHLDQQVEEFRR.R
172 – 182	1352.6367	1351.6294	1351.6520	-16.7	1	R.RVEPYGENFNK.A
183 – 192	1215.6047	1214.5975	1214.6441	-38.4	0	K.ALVQQMEQLR.Q
183 – 192	1231.6001	1230.5928	1230.6390	-37.6	0	K.ALVQQMEQLR.Q + Oxidation (M)
195 – 211	1805.9321	1804.9249	1804.9108	7.79	0	K.LGPHAGDVEGHLSFLEK.D
195 – 214	2190.2681	2189.2608	2189.1229	63.0	1	K.LGPHAGDVEGHLSFLEKDLR.D

No match to: 976.4561, 1178.9293, 1311.6724, 1325.6935, 1338.4047, 1423.7823, 1439.7916, 1508.7563, 1585.8716, 1634.8362 1813.0443, 2045.1499, 2098.2249

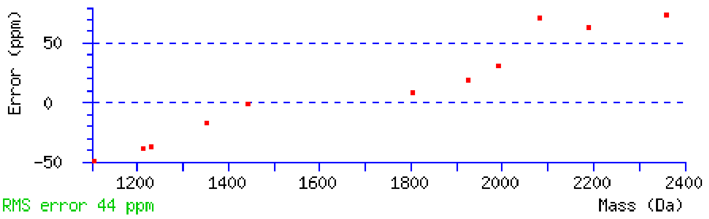

```
LOCUS       AAB59516                 244 aa            linear   PRI 08-AUG-1995
DEFINITION  apolipoprotein A-IV, partial [Homo sapiens].
ACCESSION   AAB59516
VERSION     AAB59516.1  GI:563320
DBSOURCE    locus HUMAPOAIVA accession M10373.1
KEYWORDS    .
SOURCE      Homo sapiens (human)
  ORGANISM  Homo sapiens
            Eukaryota; Metazoa; Chordata; Craniata; Vertebrata; Euteleostomi;
            Mammalia; Eutheria; Euarchontoglires; Primates; Haplorrhini;
            Catarrhini; Hominidae; Homo.
REFERENCE   1  (residues 1 to 244)
  AUTHORS   Karathanasis,S.K.
  [illegible]
```

```
   PUBMED   3931073
COMMENT     This human apolipoprotein A-IV gene sequence is found downstream
            from the A-I and C-III genes on chromosome 11 and is transcribed in
            the same direction as the A-I gene.  Occurrence of the 66 bp
            repeats seen in A-I (see segment 1) argue that this gene is closely
            related to the A-I and E genes.  A nucleotide substitution at base
            733 in another clone, lambda-gt10-3, extends the coding sequence by
            18 amino acid residues (termination codon at 787-789).  It remains
            to be estabished whether these differences represent different A-IV
            alleles or are due to cloning artifacts [1].  A potential
            polyadenylation signal was identified at positions 929-934.
            Method: conceptual translation.
FEATURES             Location/Qualifiers
     source          1..244
                     /organism="Homo sapiens"
                     /db_xref="taxon:9606"
                     /map="11q23-qter"
                     /clone="lambda-gt10-2."
                     /tissue_type="liver"
                     /dev_stage="adult"
     Protein         <1..244
                     /name="apolipoprotein A-IV"
     Region          4..189
                     /region_name="Apolipoprotein"
                     /note="Apolipoprotein A1/A4/E domain; pfam01442"
                     /db_xref="CDD:144876"
     Region          <37..184
                     /region_name="GvpL_GvpF"
                     /note="Gas vesicle synthesis protein GvpL/GvpF; pfam06386"
                     /db_xref="CDD:148160"
     CDS             1..244
                     /gene="APOA4"
                     /coded_by="M10373.1:<1..735"
                     /db_xref="GDB:G00-119-000"
```

Spot 14

Hempexin precursor

MASCOT Search Results

Protein View: gi|11321561

hemopexin precursor [Homo sapiens]

**Database:**	NCBInr
**Score:**	69
**Expect:**	0.032
**Nominal mass (M_r):**	51643
**Calculated pI:**	6.55
**Taxonomy:**	Homo sapiens

This protein sequence matches the following other entries:

- gi|1708182 from Homo sapiens
- gi|184497 from Homo sapiens
- gi|119589125 from Homo sapiens

Sequence similarity is available as an NCBI BLAST search of gi|11321561 against nr.

Search parameters

**MS data file:**	peaklist.xml
**Enzyme:**	Trypsin: cuts C-term side of KR unless next residue is P.
**Variable modifications:**	Carbamidomethyl (C), Oxidation (M)
**Mass values searched:**	12
**Mass values matched:**	6

Protein sequence coverage: 18%

Matched peptides shown in ***bold red***.

```
  1 MARVLGAPVA LGLWSLCWSL AIATPLPPTS AHGNVAEGET KPDPDVTERC
 51 SDGWSFDATT LDDNGTMLFF KGEFVWKSHK WDRELISERW KNFPSPVDAA
101 FRQGHNSVFL IKGDKVWVYP PEKKEKGYPK LLQDEFPGIP SPLDAAVECH
151 RGECQAEGVL FFQGDREWFW DLATGTMKER SWPAVGNCSS ALRWLGRYYC
201 FQGNQFLRFD PVRGEVPPRY PRDVRDYFMP CPGRGHGHRN GTGHGNSTHH
251 GPEYMRCSPH LVLSALTSDN HGATYAFSGT HYWRLDTSRD GWHSWPIAHQ
301 WPQGPSAVDA AFSWEEKLYL VQGTQVYVFL TKGGYTLVSG YPKRLEKEVG
351 TPHGIILDSV DAAFICPGSS RLHIMAGRRL WWLDLKSGAQ ATWTELPWPH
401 EKVDGALCME KSLGPNSCSA NGPGLYLIHG PNLYCYSDVE KLNAAKALPQ
451 PQNVTSLLGC TH
```

Unformatted sequence string: 462 residues (for pasting into other applications).

Sort peptides by ◉ Residue Number ○ Increasing Mass ○ Decreasing Mass

Show predicted peptides also

Start – End	Observed	Mr(expt)	Mr(calc)	ppm	M	Peptide
92 – 102	1220.4982	1219.4909	1219.5986	-88.3	0	K.NFPSPVDAAFR.Q
131 – 151	2363.8291	2362.8218	2363.1580	-142	0	K.LLQDEFPGIPSPLDAAVECHR.G + Carbamidomethyl (C)
198 – 208	1495.5565	1494.5492	1494.6714	-81.7	0	R.YYCFQGNQFLR.F + Carbamidomethyl (C)
209 – 219	1268.5666	1267.5594	1267.6673	-85.2	1	R.FDPVRGEVPPR.Y
348 – 371	2497.8784	2496.8711	2497.2271	-143	0	K.EVGTPHGIILDSVDAAFICPGSSR.L + Carbamidomethyl (C)
379 – 386	1129.5355	1128.5282	1128.6444	-103	1	R.RLWWLDLK.S

No match to: 1161.5388, 1684.7253, 1707.6141, 1838.7072, 1993.7477, 3179.8772

```
LOCUS       NP_000604                462 aa            linear   PRI 23-APR-2012
DEFINITION  hemopexin precursor [Homo sapiens].
ACCESSION   NP_000604
VERSION     NP_000604.1  GI:11321561
DBSOURCE    REFSEQ: accession NM_000613.2
KEYWORDS    .
SOURCE      Homo sapiens (human)
  ORGANISM  Homo sapiens
```

```
            Catarrhini; Hominidae; Homo.
REFERENCE   1  (residues 1 to 462)
  AUTHORS   Mauk,M.R., Smith,A. and Mauk,A.G.
  TITLE     An alternative view of the proposed alternative activities of
            hemopexin
  JOURNAL   Protein Sci. 20 (5), 791-805 (2011)
   PUBMED   21404362
  REMARK    GeneRIF: Studies indicate that hemopexin lacks the catalytic triad
            that is characteristic of many serine proteases but possesses two
            highly exposed Arg-Gly-Glu sequences that may promote interaction
            with cell surfaces.
            Review article
REFERENCE   2  (residues 1 to 462)
  AUTHORS   Tochowicz,A., Goettig,P., Evans,R., Visse,R., Shitomi,Y.,
            Palmisano,R., Ito,N., Richter,K., Maskos,K., Franke,D., Svergun,D.,
            Nagase,H., Bode,W. and Itoh,Y.
  TITLE     The dimer interface of the membrane type 1 matrix metalloproteinase
            hemopexin domain: crystal structure and biological functions
  JOURNAL   J. Biol. Chem. 286 (9), 7587-7600 (2011)
   PUBMED   21193411
  REMARK    GeneRIF: mutations of amino acids involved in the interaction
            weakened the dimer interaction of Hpx domains in solution, and
            incorporation of these mutations into the full-length enzyme
            significantly inhibited dimer-dependent functions on the cell
            surface
REFERENCE   3  (residues 1 to 462)
  AUTHORS   Bailey,S.D., Xie,C., Do,R., Montpetit,A., Diaz,R., Mohan,V.,
            Keavney,B., Yusuf,S., Gerstein,H.C., Engert,J.C. and Anand,S.
  CONSRTM   DREAM investigators
  TITLE     Variation at the NFATC2 locus increases the risk of
            thiazolidinedione-induced edema in the Diabetes REduction
            Assessment with ramipril and rosiglitazone Medication (DREAM) study
  JOURNAL   Diabetes Care 33 (10), 2250-2253 (2010)
   PUBMED   20628086
  REMARK    GeneRIF: Observational study of gene-disease association,
            gene-environment interaction, and pharmacogenomic / toxicogenomic.
            (HuGE Navigator)
REFERENCE   4  (residues 1 to 462)
  AUTHORS   Law,M.L., Cai,G.Y., Hartz,J.A., Jones,C. and Kao,F.T.
  TITLE     The hemopexin gene maps to the same location as the beta-globin
            gene cluster on human chromosome 11
  JOURNAL   Genomics 3 (1), 48-52 (1988)
   PUBMED   3220477
REFERENCE   5  (residues 1 to 462)
  AUTHORS   Morgan,W.T., Alam,J., Deaciuc,V., Muster,P., Tatum,F.M. and
            Smith,A.
  TITLE     Interaction of hemopexin with Sn-protoporphyrin IX, an inhibitor of
            heme oxygenase. Role for hemopexin in hepatic uptake of
            Sn-protoporphyrin IX and induction of mRNA for heme oxygenase
  JOURNAL   J. Biol. Chem. 263 (17), 8226-8231 (1988)
   PUBMED   3372522
REFERENCE   6  (residues 1 to 462)
  AUTHORS   Smith,A., Tatum,F.M., Muster,P., Burch,M.K. and Morgan,W.T.
  TITLE     Importance of ligand-induced conformational changes in hemopexin
            for receptor-mediated heme transport
  JOURNAL   J. Biol. Chem. 263 (11), 5224-5229 (1988)
   PUBMED   2833500
REFERENCE   7  (residues 1 to 462)
  AUTHORS   Altruda,F., Poli,V., Restagno,G. and Silengo,L.
  TITLE     Structure of the human hemopexin gene and evidence for
            intron-mediated evolution
  JOURNAL   J. Mol. Evol. 27 (2), 102-108 (1988)
   PUBMED   2842511
REFERENCE   8  (residues 1 to 462)
  AUTHORS   Taketani,S., Kohno,H., Naitoh,Y. and Tokunaga,R.
  TITLE     Isolation of the hemopexin receptor from human placenta
  JOURNAL   J. Biol. Chem. 262 (18), 8668-8671 (1987)
   PUBMED   3036819
REFERENCE   9  (residues 1 to 462)
  AUTHORS   Altruda,F., Poli,V., Restagno,G., Argos,P., Cortese,R. and
            Silengo,L.
  TITLE     The primary structure of human hemopexin deduced from cDNA
            sequence: evidence for internal, repeating homology
  JOURNAL   Nucleic Acids Res. 13 (11), 3841-3859 (1985)
   PUBMED   2989777
REFERENCE   10 (residues 1 to 462)
  AUTHORS   Takahashi,N., Takahashi,Y. and Putnam,F.W.
  TITLE     Complete amino acid sequence of human hemopexin, the heme-binding
            protein of serum
  JOURNAL   Proc. Natl. Acad. Sci. U.S.A. 82 (1), 73-77 (1985)
   PUBMED   3855550
REFERENCE   11 (sites)
  AUTHORS   Takahashi,N., Takahashi,Y. and Putnam,F.W.
  TITLE     Complete amino acid sequence of human hemopexin, the heme-binding
            protein of serum
  JOURNAL   Proc. Natl. Acad. Sci. U.S.A. 82 (1), 73-77 (1985)
   PUBMED   3855550
REFERENCE   12 (sites)
  AUTHORS   Frantikova,V., Borvak,J., Kluh,I. and Moravek,L.
  TITLE     Amino acid sequence of the N-terminal region of human hemopexin
  JOURNAL   FEBS Lett. 178 (2), 213-216 (1984)
   PUBMED   6510521
```

```
            unusual clustering of tryptophan residues
  JOURNAL   Proc. Natl. Acad. Sci. U.S.A. 81 (7), 2021-2025 (1984)
   PUBMED   6371807
COMMENT     REVIEWED REFSEQ: This record has been curated by NCBI staff. The
            reference sequence was derived from BC005395.1, AK313648.1,
            AV655383.1 and AC084337.7.
            
            Summary: This gene encodes a plasma glycoprotein that binds heme
            with high affinity. The encoded protein is an acute phase protein
            that transports heme from the plasma to the liver and may be
            involved in protecting cells from oxidative stress. [provided by
            RefSeq, Apr 2009].
            
            Publication Note:  This RefSeq record includes a subset of the
            publications that are available for this gene. Please see the Gene
            record to access additional publications.
FEATURES             Location/Qualifiers
     source          1..462
                     /organism="Homo sapiens"
                     /db_xref="taxon:9606"
                     /chromosome="11"
                     /map="11p15.5-p15.4"
     Protein         1..462
                     /product="hemopexin precursor"
                     /EC_number="3.2.1.35"
                     /note="beta-1B-glycoprotein"
     sig_peptide     1..23
                     /calculated_mol_wt=2399
     mat_peptide     24..462
                     /product="hemopexin"
                     /calculated_mol_wt=49296
     Region          24..47
                     /region_name="O-glycosylated at one, two and three sites"
                     /experiment="experimental evidence, no additional details
                     recorded"
                     /note="propagated from UniProtKB/Swiss-Prot (P02790.2)"
     Site            24
                     /site_type="glycosylation"
                     /experiment="experimental evidence, no additional details
                     recorded"
                     /citation=[12]
                     /citation=[13]
     Region          47..231
                     /region_name="HX"
                     /note="Hemopexin-like repeats.; Hemopexin is a
                     heme-binding protein that transports heme to the liver.
                     Hemopexin-like repeats occur in vitronectin and some
                     matrix metalloproteinases family (matrixins). The HX
                     repeats of some matrixins bind tissue inhibitor of...;
                     cd00094"
                     /db_xref="CDD:28978"
     Bond            bond(50,231)
                     /bond_type="disulfide"
                     /experiment="experimental evidence, no additional details
                     recorded"
                     /note="disulfide bridge bond"
                     /citation=[10]
     Site            order(57,59,98,100,144,146,189,191)
                     /site_type="metal-binding"
                     /note="Metal binding sites [ion binding]"
                     /db_xref="CDD:28978"
     Site            64
                     /site_type="glycosylation"
                     /experiment="experimental evidence, no additional details
                     recorded"
                     /citation=[12]
                     /citation=[13]
     Bond            bond(149,154)
                     /bond_type="disulfide"
                     /experiment="experimental evidence, no additional details
                     recorded"
                     /note="disulfide bridge bond"
                     /citation=[10]
                     /citation=[12]
     Site            187
                     /site_type="glycosylation"
                     /experiment="experimental evidence, no additional details
                     recorded"
                     /citation=[12]
                     /citation=[13]
     Bond            bond(188,200)
                     /bond_type="disulfide"
                     /experiment="experimental evidence, no additional details
                     recorded"
                     /note="disulfide bridge bond"
                     /citation=[10]
     Site            240
                     /site_type="glycosylation"
                     /experiment="experimental evidence, no additional details
                     recorded"
                     /citation=[12]
                     /citation=[13]
```

```
                    recorded"
                    /citation=[13]
Region              256..460
                    /region_name="HX"
                    /note="Hemopexin-like repeats.; Hemopexin is a
                    heme-binding protein that transports heme to the liver.
                    Hemopexin-like repeats occur in vitronectin and some
                    matrix metalloproteinases family (matrixins). The HX
                    repeats of some matrixins bind tissue inhibitor of...;
                    cd00094"
                    /db_xref="CDD:28978"
Bond                bond(257,460)
                    /bond_type="disulfide"
                    /experiment="experimental evidence, no additional details
                    recorded"
                    /note="disulfide bridge bond"
                    /citation=[10]
Site                order(264,266,309,311,361,363,404,406)
                    /site_type="metal-binding"
                    /note="Metal binding sites [ion binding]"
                    /db_xref="CDD:28978"
Bond                bond(366,408)
                    /bond_type="disulfide"
                    /experiment="experimental evidence, no additional details
                    recorded"
                    /note="disulfide bridge bond"
                    /citation=[10]
Bond                bond(418,435)
                    /bond_type="disulfide"
                    /experiment="experimental evidence, no additional details
                    recorded"
                    /note="disulfide bridge bond"
                    /citation=[10]
Site                453
                    /site_type="glycosylation"
                    /experiment="experimental evidence, no additional details
                    recorded"
                    /citation=[13]
CDS                 1..462
                    /gene="HPX"
                    /gene_synonym="HX"
                    /coded_by="NM_000613.2:62..1450"
                    /db_xref="CCDS:CCDS7763.1"
                    /db_xref="GeneID:3263"
                    /db_xref="HGNC:5171"
                    /db_xref="HPRD:00793"
                    /db_xref="MIM:142290"
```

Spot 15

α1-antitrypsin

MASCOT Search Results

Protein View: gi|157831596

Chain A, Alpha1-Antitrypsin

**Database:**	NCBInr
**Score:**	56
**Expect:**	0.66
**Nominal mass (M_r):**	44223
**Calculated pI:**	5.37
**Taxonomy:**	Homo sapiens

Sequence similarity is available as an NCBI BLAST search of gi|157831596 against nr.

Search parameters

**MS data file:**	peaklist.xml
**Enzyme:**	Trypsin: cuts C-term side of KR unless next residue is P.
**Variable modifications:**	Carbamidomethyl (C), Oxidation (M)
**Mass values searched:**	10
**Mass values matched:**	5

Protein sequence coverage: 17%

Matched peptides shown in ***bold red***.

1 EDPQGDAAQK TDTSHHDQDH PTFNK**ITPNL** **AEFAFSLYR**Q LAHQSNSTNI
51 FFSPVSIAAA FAMLSLGAKG DTHDEILEGL NFNLTEIPEA QIHEGFQELL
101 RTLNQPDSQL QLTTGNGLFL SEGLKLVDKF LEDVKK**LYHS** **EAFTVNFGDT**
151 **EEAKK**QINDY VEKGTQGKIV DLVK**ELDRDT** **VFALVNYIFF** **KGKWERPFEV**
201 **K**DTEEEDFHV DQVTTVKVPM MKRLGMFNIQ HCKKLSSWVL LMKYLGNATA
251 IFFLPDEGKL QHLENELTHD IITK**FLENED** **RR**SASLHLPK LSITGTYDLK
301 SVLGQLGITK VFSNGADLSG VTEEAPLKLS KAVHKAVLTI DEKGTEAAGA
351 MFLEAIPMSI PPEVKFNKPF VFLMIEQNTK SPLFMGKVVN PTQK

Unformatted sequence string: 394 residues (for pasting into other applications).

Sort peptides by ◉ Residue Number ○ Increasing Mass ○ Decreasing Mass

Show predicted peptides also

Start – End	Observed	Mr(expt)	Mr(calc)	ppm	M	Peptide
26 – 39	1641.8447	1640.8375	1640.8562	-11.4	0	K.ITPNLAEFAFSLYR.Q
137 – 155	2185.9648	2184.9576	2185.0327	-34.4	1	K.LYHSEAFTVNFGDTEEAKK.Q
175 – 191	2090.0237	2089.0164	2089.0884	-34.5	1	K.ELDRDTVFALVNYIFFK.G
192 – 201	1275.6709	1274.6636	1274.6771	-10.6	1	K.GKWERPFEVK.D
275 – 282	1078.5032	1077.4959	1077.5203	-22.6	1	K.FLENEDRR.S

No match to: 1216.6038, 2575.1479, 2691.0833, 2807.0867, 3148.2639

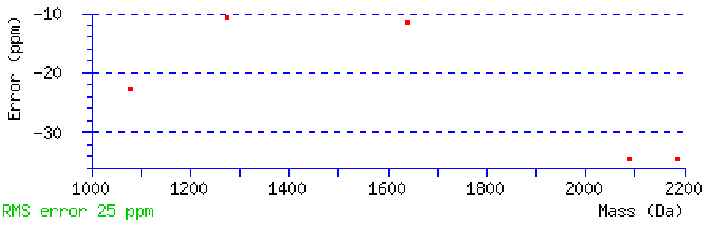


```
LOCUS       1KCT_A                  394 aa            linear   PRI 10-JUL-2009
DEFINITION  Chain A, Alpha1-Antitrypsin.
ACCESSION   1KCT_A
VERSION     1KCT_A  GI:157831596
DBSOURCE    pdb: molecule 1KCT, chain 65, release Jun 18, 2009;
            deposition: Aug 6, 1996;
            class: Serine Protease Inhibitor;
            source: Mol_id: 1; Organism_scientific: Homo Sapiens;
            Organism_common: Human; Organism_taxid: 9606; Gene: A1at;
            Expression_system: Escherichia Coli Bl21(De3);
            Expression_system_taxid: 469008; Expression_system_strain:
            Bl21(De3); Expression_system_plasmid: Pet-8c;
            Expression_system_gene: A1at;
            Exp. method: X-Ray Diffraction.
KEYWORDS    .
SOURCE      Homo sapiens (human)
  ORGANISM  Homo sapiens
```

```
REFERENCE   1  (residues 1 to 394)
  AUTHORS   Song,H.K., Lee,K.N., Kwon,K.S., Yu,M.H. and Suh,S.W.
  TITLE     Crystal structure of an uncleaved alpha 1-antitrypsin reveals the
            conformation of its inhibitory reactive loop
  JOURNAL   FEBS Lett. 377 (2), 150-154 (1995)
   PUBMED   8543039
REFERENCE   2  (residues 1 to 394)
  AUTHORS   Song,H.K. and Suh,S.W.
  TITLE     Direct Submission
  JOURNAL   Submitted (06-AUG-1996)
COMMENT     SEQRES.
FEATURES             Location/Qualifiers
     source          1..394
                     /organism="Homo sapiens"
                     /db_xref="taxon:9606"
     Region          24..391
                     /region_name="PHA02660"
                     /note="serpin-like protein; Provisional"
                     /db_xref="CDD:165039"
     SecStr          27..44
                     /sec_str_type="helix"
                     /note="helix 1"
     Region          31..388
                     /region_name="alpha-1-antitrypsin_like"
                     /note="alpha-1-antitrypsin_like. This family contains a
                     variety of different members of clade A of the serpin
                     superfamily. They include the classical serine proteinase
                     inhibitors, alpha-1-antitrypsin and
                     alpha-1-antichymotrypsin, protein C inhibitor; cd02056"
                     /db_xref="CDD:29131"
     SecStr          49..54
                     /sec_str_type="sheet"
                     /note="strand 1"
     SecStr          55..63
                     /sec_str_type="helix"
                     /note="helix 2"
     SecStr          94..101
                     /sec_str_type="helix"
                     /note="helix 3"
     SecStr          110..121
                     /sec_str_type="sheet"
                     /note="strand 2"
     SecStr          128..137
                     /sec_str_type="helix"
                     /note="helix 4"
     SecStr          157..164
                     /sec_str_type="helix"
                     /note="helix 5"
     SecStr          181..192
                     /sec_str_type="sheet"
                     /note="strand 3"
     SecStr          193..196
                     /sec_str_type="sheet"
                     /note="strand 4"
     SecStr          207..211
                     /sec_str_type="sheet"
                     /note="strand 5"
     SecStr          213..217
                     /sec_str_type="sheet"
                     /note="strand 6"
     SecStr          219..224
                     /sec_str_type="sheet"
                     /note="strand 7"
     SecStr          225..233
                     /sec_str_type="sheet"
                     /note="strand 8"
     SecStr          235..244
                     /sec_str_type="sheet"
                     /note="strand 9"
     SecStr          247..256
                     /sec_str_type="sheet"
                     /note="strand 10"
     SecStr          280..283
                     /sec_str_type="sheet"
                     /note="strand 11"
     SecStr          284..290
                     /sec_str_type="sheet"
                     /note="strand 12"
     SecStr          293..300
                     /sec_str_type="sheet"
                     /note="strand 13"
     SecStr          330..338
                     /sec_str_type="sheet"
                     /note="strand 14"
     SecStr          343..346
                     /sec_str_type="sheet"
                     /note="strand 15"
     Site            order(344..354,364..369)
                     /site_type="other"
                     /note="reactive center loop"
                     /db_xref="CDD:29131"
     SecStr          362..366
```

```
                  /sec_str_type="sheet"
                  /note="strand 17"
SecStr            379..389
                  /sec_str_type="sheet"
                  /note="strand 18"
```

Spot 16

α1-antitrypsin

{MATRIX SCIENCE} MASCOT Search Results

Protein View: gi|157831596

Chain A, Alpha1-Antitrypsin

**Database:**	NCBInr
**Score:**	89
**Expect:**	0.0003
**Nominal mass (M_r):**	44223
**Calculated pI:**	5.37
**Taxonomy:**	~~Homo sapiens~~

Sequence similarity is available as ~~an NCBI BLAST search of gi|157831596 against nr~~.

Search parameters

**MS data file:**	peaklist.xml
**Enzyme:**	Trypsin: cuts C-term side of KR unless next residue is P.
**Variable modifications:**	~~Carbamidomethyl (C)~~, ~~Oxidation (M)~~
**Mass values searched:**	39
**Mass values matched:**	11

Protein sequence coverage: 35%

Matched peptides shown in ***bold red***.

```
  1 EDPQGDAAQK TDTSHHDQDH PTFNKITPNL AEFAFSLYRQ LAHQSNSTNI
 51 FFSPVSIAAA FAMLSLGAKG DTHDEILEGL NFNLTEIPEA QIHEGFQELL
101 RTLNQPDSQL QLTTGNGLFL SEGLKLVDKF LEDVKKLYHS EAFTVNFGDT
151 EEAKKQINDY VEKGTQGKIV DLVKELDRDT VFALVNYIFF KGKWERPFEV
201 KDTEEEDFHV DQVTTVKVPM MKRLGMFNIQ HCKKLSSWVL LMKYLGNATA
251 IFFLPDEGKL QHLENELTHD IITKFLENED RRSASLHLPK LSITGTYDLK
301 SVLGQLGITK VFSNGADLSG VTEEAPLKLS KAVHKAVLTI DEKGTEAAGA
351 MFLEAIPMSI PPEVKFNKPF VFLMIEQNTK SPLFMGKVVN PTQK
```

Unformatted sequence string: ~~394 residues~~ (for pasting into other applications).

Sort peptides by ◉ Residue Number ○ Increasing Mass ○ Decreasing Mass

Show predicted peptides also

Start – End	Observed	Mr(expt)	Mr(calc)	ppm	M	Peptide
11 – 25	1779.8910	1778.8837	1778.7609	69.1	0	K.TDTSHHDQDHPTFNK.I
26 – 39	1641.9712	1640.9639	1640.8562	65.6	0	K.ITPNLAEFAFSLYR.Q
137 – 155	2186.1108	2185.1036	2185.0327	32.4	1	K.LYHSEAFTVNFGDTEEAKK.Q
192 – 201	1275.7202	1274.7129	1274.6771	28.1	1	K.GKWERPFEVK.D
202 – 217	1891.9780	1890.9708	1890.8483	64.7	0	K.DTEEEDFHVDQVTTVK.V
223 – 233	1419.8153	1418.8080	1418.6911	82.4	1	K.RLGMFNIQHCK.K + Carbamidomethyl (C); Oxidation (M)
260 – 274	1804.0609	1803.0536	1802.9526	56.0	0	K.LQHLENELTHDIITK.F
260 – 281	2707.1956	2706.1883	2706.3613	-63.9	1	K.LQHLENELTHDIITKFLENEDR.R
275 – 282	1078.5677	1077.5605	1077.5203	37.3	1	K.FLENEDRR.S
311 – 328	1834.0392	1833.0319	1832.9156	63.5	0	K.VFSNGADLSGVTEEAPLK.L
366 – 380	1872.0781	1871.0708	1870.9651	56.5	0	K.FNKPFVFLMIEQNTK.S + Oxidation (M)

No match to: 915.4419, 1063.3807, 1170.7000, 1254.7875, 1307.7749, 1326.8462, 1388.8054, 1404.8064, 1444.7032, 1529.8993 1695.0280, 1737.1493, 2092.9014, 2211.1663, 2265.0105, 2328.2542, 2371.0925, 2518.0486, 2521.0688, 2575.3713, 2711.2178, 2722.2324, 2835.2700, 2850.2341, 2950.2576, 2974.2698, 3102.2974, 3180.3398

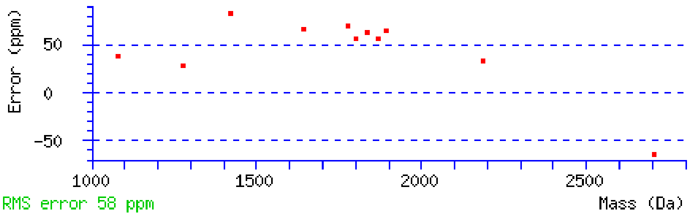


```
LOCUS       1KCT_A                   394 aa            linear   PRI 10-JUL-2009
DEFINITION  Chain A, Alpha1-Antitrypsin.
ACCESSION   1KCT_A
VERSION     1KCT_A  GI:157831596
DBSOURCE    pdb: molecule 1KCT, chain 65, release Jun 18, 2009;
            deposition: Aug 6, 1996;
            class: Serine Protease Inhibitor;
            source: Mol_id: 1; Organism_scientific: Homo Sapiens;
```

```
            Bl21(De3); Expression_system_plasmid: Pet-8c;
            Expression_system_gene: A1at;
            Exp. method: X-Ray Diffraction.
KEYWORDS    .
SOURCE      Homo sapiens (human)
  ORGANISM  Homo sapiens
            Eukaryota; Metazoa; Chordata; Craniata; Vertebrata; Euteleostomi;
            Mammalia; Eutheria; Euarchontoglires; Primates; Haplorrhini;
            Catarrhini; Hominidae; Homo.
REFERENCE   1  (residues 1 to 394)
  AUTHORS   Song,H.K., Lee,K.N., Kwon,K.S., Yu,M.H. and Suh,S.W.
  TITLE     Crystal structure of an uncleaved alpha 1-antitrypsin reveals the
            conformation of its inhibitory reactive loop
  JOURNAL   FEBS Lett. 377 (2), 150-154 (1995)
   PUBMED   8543039
REFERENCE   2  (residues 1 to 394)
  AUTHORS   Song,H.K. and Suh,S.W.
  TITLE     Direct Submission
  JOURNAL   Submitted (06-AUG-1996)
COMMENT     SEQRES.
FEATURES             Location/Qualifiers
     source          1..394
                     /organism="Homo sapiens"
                     /db_xref="taxon:9606"
     Region          24..391
                     /region_name="PHA02660"
                     /note="serpin-like protein; Provisional"
                     /db_xref="CDD:165039"
     SecStr          27..44
                     /sec_str_type="helix"
                     /note="helix 1"
     Region          31..388
                     /region_name="alpha-1-antitrypsin_like"
                     /note="alpha-1-antitrypsin_like. This family contains a
                     variety of different members of clade A of the serpin
                     superfamily. They include the classical serine proteinase
                     inhibitors, alpha-1-antitrypsin and
                     alpha-1-antichymotrypsin, protein C inhibitor; cd02056"
                     /db_xref="CDD:29131"
     SecStr          49..54
                     /sec_str_type="sheet"
                     /note="strand 1"
     SecStr          55..63
                     /sec_str_type="helix"
                     /note="helix 2"
     SecStr          94..101
                     /sec_str_type="helix"
                     /note="helix 3"
     SecStr          110..121
                     /sec_str_type="sheet"
                     /note="strand 2"
     SecStr          128..137
                     /sec_str_type="helix"
                     /note="helix 4"
     SecStr          157..164
                     /sec_str_type="helix"
                     /note="helix 5"
     SecStr          181..192
                     /sec_str_type="sheet"
                     /note="strand 3"
     SecStr          193..196
                     /sec_str_type="sheet"
                     /note="strand 4"
     SecStr          207..211
                     /sec_str_type="sheet"
                     /note="strand 5"
     SecStr          213..217
                     /sec_str_type="sheet"
                     /note="strand 6"
     SecStr          219..224
                     /sec_str_type="sheet"
                     /note="strand 7"
     SecStr          225..233
                     /sec_str_type="sheet"
                     /note="strand 8"
     SecStr          235..244
                     /sec_str_type="sheet"
                     /note="strand 9"
     SecStr          247..256
                     /sec_str_type="sheet"
                     /note="strand 10"
     SecStr          280..283
                     /sec_str_type="sheet"
                     /note="strand 11"
     SecStr          284..290
                     /sec_str_type="sheet"
                     /note="strand 12"
     SecStr          293..300
                     /sec_str_type="sheet"
                     /note="strand 13"
     SecStr          330..338
                     /sec_str_type="sheet"
```

```
                     /note="strand 15"
     Site            order(344..354,364..369)
                     /site_type="other"
                     /note="reactive center loop"
                     /db_xref="CDD:29131"
     SecStr          362..366
                     /sec_str_type="sheet"
                     /note="strand 16"
     SecStr          369..377
                     /sec_str_type="sheet"
                     /note="strand 17"
     SecStr          379..389
                     /sec_str_type="sheet"
                     /note="strand 18"
```

Spot 17

Vitamin D binding protein

MASCOT Search Results

Protein View: gi|181482

serum vitamin D-binding protein precursor [Homo sapiens]

**Database:**	NCBInr
**Score:**	313
**Nominal mass (M_r):**	53015
**Calculated pI:**	5.40
**Taxonomy:**	Homo sapiens

Sequence similarity is available as an NCBI BLAST search of gi|181482 against nr.

Search parameters

**MS data file:**	Spot-17-060316 CYWu-14.pkl
**Enzyme:**	Trypsin: cuts C-term side of KR unless next residue is P.
**Variable modifications:**	Carbamidomethyl (C), Oxidation (M)

Protein sequence coverage: 19%

Matched peptides shown in ***bold red***.

```
  1 MKRVLVLLLA VAFGHALERG RDYEKNKVCK EFSHLGKEDF TSLSLVLYSR
 51 KFPSGTFEQV SQLVKEVVSL TEACCAEGAD PDCYDTRTSA LSAKSCESNS
101 PFPVHPGTAE CCTKEGLERK LCMAALKHQP QEFPTYVEPT NDEICEAFRK
151 DPKEYANQFM WEYSTNYEQA PLSLLVSYTK SYLSMVGSCC TSASPTVCFL
201 KERLQLKHLS LLTTLSNRVC SQYAAYGEKK SRLSNLIKLA QKVPTADLED
251 VLPLAEDITN ILSKCCESAS EDCMAKELPE HTVKLCDNLS TKNSKFEDCC
301 QEKTAMDVFV CTYFMPAAQL PELPDVRLPT NKDVCDPGNT KVMDKYTFEL
351 SRRTHLPEVF LSKVLEPTLK SLGECCDVED STTCFNAKGP LLKKELSSFI
401 DKGQELCADY SENTFTEYKK KLAERLKAKL PEATPTELAK LVNKRSDFAS
451 NCCSINSPPL YCDSEIDAEL KNIL
```

Unformatted sequence string: 474 residues (for pasting into other applications).

Sort peptides by ◉ Residue Number ○ Increasing Mass ○ Decreasing Mass

Show predicted peptides also

Query	Start – End	Observed	Mr(expt)	Mr(calc)	Delta	M	Score	Expect	Rank	U	Peptide
37	95 – 114	755.5052	2263.4938	2263.9297	-0.4359	0	47	0.014	1	U	K.SCESNSPFPVHPGTAECCTK.E + 3 Carbamidomethyl (C)
25	208 – 218	627.7582	1253.5018	1253.7092	-0.2073	0	49	0.011	1	U	K.HLSLLTTLSNR.V
26	219 – 229	638.1890	1274.3634	1274.5601	-0.1967	0	31	0.73	1	U	R.VCSQYAAYGEK.K + Carbamidomethyl (C)
13	285 – 292	475.6812	949.3478	949.4539	-0.1060	0	40	0.12	1	U	K.LCDNLSTK.N + Carbamidomethyl (C)
22	296 – 303	558.1307	1114.2468	1114.4059	-0.1591	0	8	1.6e+02	5	U	K.FEDCCQEK.T + 2 Carbamidomethyl (C)
31	342 – 352	468.8389	1403.4949	1403.6755	-0.1806	1	50	0.0098	1	U	K.VMDKYTFELSR.R + Oxidation (M)
11	346 – 352	458.1790	914.3434	914.4498	-0.1063	0	31	0.77	1	U	K.YTFELSR.R
28	353 – 363	442.8670	1325.5792	1325.7456	-0.1664	1	31	0.74	1	U	R.RTHLPEVFLSK.V
30	428 – 440	684.7847	1367.5548	1367.7660	-0.2111	1	32	0.77	1	U	K.AKLPEATPTELAK.L

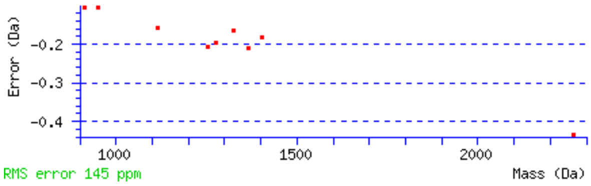

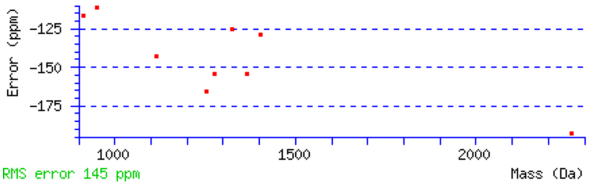


```
LOCUS       AAA52173                 474 aa            linear   PRI 02-NOV-1994
DEFINITION  serum vitamin D-binding protein precursor [Homo sapiens].
ACCESSION   AAA52173
VERSION     AAA52173.1  GI:181482
DBSOURCE    locus HUMDBP accession M12654.1
KEYWORDS    .
SOURCE      Homo sapiens (human)
  ORGANISM  Homo sapiens
            Eukaryota; Metazoa; Chordata; Craniata; Vertebrata; Euteleostomi;
            Mammalia; Eutheria; Euarchontoglires; Primates; Haplorrhini;
            Catarrhini; Hominidae; Homo.
REFERENCE   1  (residues 1 to 474)
  AUTHORS   Cooke,N.E. and David,E.V.
  TITLE     Serum vitamin D-binding protein is a third member of the albumin
            and alpha fetoprotein gene family
  JOURNAL   J. Clin. Invest. 76 (6), 2420-2424 (1985)
   PUBMED   2416779
COMMENT     Draft entry and sequence in computer readable form for [1] kindly
            provided by N.E.Cooke, 24-JUN-1986.
            Method: conceptual translation.
FEATURES             Location/Qualifiers
     source          1..474
                     /organism="Homo sapiens"
                     /db_xref="taxon:9606"
                     /map="4q12-q13"
     Protein         1..474
                     /name="serum vitamin D-binding protein precursor"
     sig_peptide     1..16
                     /note="serum vitamin D-binding protein signal peptide"
     mat_peptide     17..474
                     /product="serum vitamin D-binding protein"
     Region          26..206
```

```
                     alpha-fetoprotein which binds various cations, fatty acids
                     and bilirubin; vitamin D-binding protein which binds to
                     vitamin D, its metabolites, and fatty acids; cd00015"
                     /db_xref="CDD:28899"
     Site            order(48,52,67,88,92,95,120,123..124)
                     /site_type="binding"
                     /db_xref="CDD:28899"
     Site            order(156,159..160,163,179,182)
                     /site_type="binding"
                     /db_xref="CDD:28899"
     Region          217..392
                     /region_name="ALBUMIN"
                     /note="Albumin domain, contains five or six internal
                     disulphide bonds; albuminoid superfamily includes
                     alpha-fetoprotein which binds various cations, fatty acids
                     and bilirubin; vitamin D-binding protein which binds to
                     vitamin D, its metabolites, and fatty acids; cl00031"
                     /db_xref="CDD:206791"
     Site            order(239,243,258,278,282,285,309,312..313)
                     /site_type="binding"
                     /db_xref="CDD:28899"
     Site            order(343,346..347,350,365,368)
                     /site_type="binding"
                     /db_xref="CDD:28899"
     Region          405..472
                     /region_name="VitD-bind_III"
                     /note="Vitamin D binding protein, domain III; pfam09164"
                     /db_xref="CDD:204156"
     CDS             1..474
                     /gene="GC"
                     /coded_by="M12654.1:29..1453"
                     /db_xref="GDB:G00-119-263"
```

Spot 18

Vitamin D binding protein

MATRIX SCIENCE MASCOT Search Results

Protein View: gi|34785355

Group-specific component (vitamin D binding protein) [Homo sapiens]

**Database:**	NCBInr
**Score:**	133
**Expect:**	1.2e-08
**Nominal mass (M_r):**	52902
**Calculated pI:**	5.33
**Taxonomy:**	~~Homo sapiens~~

This protein sequence matches the following other entries:

- ~~gi|158255090~~ from ~~Homo sapiens~~
- ~~gi|164691123~~ from ~~Homo sapiens~~

Sequence similarity is available as ~~an NCBI BLAST search of gi|34785355 against nr~~.

Search parameters

**MS data file:**	peaklist.xml
**Enzyme:**	Trypsin: cuts C-term side of KR unless next residue is P.
**Variable modifications:**	~~Carbamidomethyl (C)~~, ~~Oxidation (M)~~
**Mass values searched:**	19
**Mass values matched:**	13

Protein sequence coverage: 30%

Matched peptides shown in ***bold red***.

1 MKRVLVLLLA VAFGHALERG RDYEKNKVCK **EFSHLGKEDF** **TSLSLVLYSR**
51 **KFPSGTFEQV** **SQLVKEVVSL** **TEACCAEGAD** **PDCYDTR**TSA LSAK**SCESNS**
101 **PFPVHPGTAE** **CCTK**EGLERK LCMAALK**HQP** **QEFPTYVEPT** **NDEICEAFRK**
151 DPKEYANQFM WEYSTNYGQA PLSLLVSYTK SYLSMVGSCC TSASPTVCFL
201 KERLQLK**HLS** **LLTTLSNRVC** **SQYAAYGEK**K SRLSNLIKLA QKVPTADLED
251 VLPLAEDITN ILSKCCESAS EDCMAKELPE HTVKLCDNLS TKNSKFEDCC
301 QEKTAMDVFV CTYFMPAAQL PELPDVELPT NKDVCDPGNT K**VMDKYTFEL**
351 **SRRTHLPEVF** **LSK**VLEPTLK SLGECCDVED STTCFNAKGP LLKKELSSFI
401 DKGQELCADY SENTFTEYKK KLAERLKAKL PDATPTELAK LVNKRSDFAS
451 NCCSINSPPL YCDSEIDAEL KNIL

Unformatted sequence string: ~~474 residues~~ (for pasting into other applications).

Sort peptides by ◉ Residue Number ○ Increasing Mass ○ Decreasing Mass

Show predicted peptides also

Start – End	Observed	Mr(expt)	Mr(calc)	ppm	M	Peptide
31 – 50	2328.1658	2327.1585	2327.1797	-9.13	1	K.EFSHLGKEDFTSLSLVLYSR.K
51 – 65	1694.9000	1693.8927	1693.9039	-6.60	1	R.KFPSGTFEQVSQLVK.E
66 – 87	2517.8616	2516.8543	2517.0094	-61.6	0	K.EVVSLTEACCAEGADPDCYDTR.T + 3 Carbamidomethyl (C)
95 – 114	2264.8792	2263.8719	2263.9297	-25.5	0	K.SCESNSPFPVHPGTAECCTK.E + 3 Carbamidomethyl (C)
128 – 149	2707.1255	2706.1182	2706.2020	-31.0	0	K.HQPQEFPTYVEPTNDEICEAFR.K + Carbamidomethyl (C)
128 – 150	2835.1292	2834.1219	2834.2970	-61.8	1	K.HQPQEFPTYVEPTNDEICEAFRK.D + Carbamidomethyl (C)
208 – 218	1254.7236	1253.7164	1253.7092	5.73	0	K.HLSLLTTLSNR.V
219 – 229	1275.5934	1274.5861	1274.5601	20.4	0	R.VCSQYAAYGEK.K + Carbamidomethyl (C)
342 – 352	1388.7064	1387.6991	1387.6806	13.4	1	K.VMDKYTFELSR.R
342 – 352	1404.7148	1403.7076	1403.6755	22.9	1	K.VMDKYTFELSR.R + Oxidation (M)
346 – 352	915.4381	914.4308	914.4498	-20.7	0	K.YTFELSR.R
353 – 363	1326.7743	1325.7670	1325.7456	16.2	1	R.RTHLPEVFLSK.V
354 – 363	1170.6486	1169.6413	1169.6445	-2.71	0	R.THLPEVFLSK.V

No match to: 1078.5139, 1307.6805, 1420.7223, 1641.8333, 1779.7733, 2185.9224

LOCUS AAH57228 474 aa linear PRI 15-JUL-2006

```
ACCESSION   AAH57228
VERSION     AAH57228.1  GI:34785355
DBSOURCE    accession BC057228.1
KEYWORDS    MGC.
SOURCE      Homo sapiens (human)
  ORGANISM  Homo sapiens
            Eukaryota; Metazoa; Chordata; Craniata; Vertebrata; Euteleostomi;
            Mammalia; Eutheria; Euarchontoglires; Primates; Haplorrhini;
            Catarrhini; Hominidae; Homo.
REFERENCE   1  (residues 1 to 474)
  AUTHORS   Strausberg,R.L., Feingold,E.A., Grouse,L.H., Derge,J.G.,
            Klausner,R.D., Collins,F.S., Wagner,L., Shenmen,C.M., Schuler,G.D.,
            Altschul,S.F., Zeeberg,B., Buetow,K.H., Schaefer,C.F., Bhat,N.K.,
            Hopkins,R.F., Jordan,H., Moore,T., Max,S.I., Wang,J., Hsieh,F.,
            Diatchenko,L., Marusina,K., Farmer,A.A., Rubin,G.M., Hong,L.,
            Stapleton,M., Soares,M.B., Bonaldo,M.F., Casavant,T.L.,
            Scheetz,T.E., Brownstein,M.J., Usdin,T.B., Toshiyuki,S.,
            Carninci,P., Prange,C., Raha,S.S., Loquellano,N.A., Peters,G.J.,
            Abramson,R.D., Mullahy,S.J., Bosak,S.A., McEwan,P.J.,
            McKernan,K.J., Malek,J.A., Gunaratne,P.H., Richards,S.,
            Worley,K.C., Hale,S., Garcia,A.M., Gay,L.J., Hulyk,S.W.,
            Villalon,D.K., Muzny,D.M., Sodergren,E.J., Lu,X., Gibbs,R.A.,
            Fahey,J., Helton,E., Ketteman,M., Madan,A., Rodrigues,S.,
            Sanchez,A., Whiting,M., Madan,A., Young,A.C., Shevchenko,Y.,
            Bouffard,G.G., Blakesley,R.W., Touchman,J.W., Green,E.D.,
            Dickson,M.C., Rodriguez,A.C., Grimwood,J., Schmutz,J., Myers,R.M.,
            Butterfield,Y.S., Krzywinski,M.I., Skalska,U., Smailus,D.E.,
            Schnerch,A., Schein,J.E., Jones,S.J. and Marra,M.A.
  CONSRTM   Mammalian Gene Collection Program Team
  TITLE     Generation and initial analysis of more than 15,000 full-length
            human and mouse cDNA sequences
  JOURNAL   Proc. Natl. Acad. Sci. U.S.A. 99 (26), 16899-16903 (2002)
   PUBMED   12477932
REFERENCE   2  (residues 1 to 474)
  CONSRTM   NIH MGC Project
  TITLE     Direct Submission
  JOURNAL   Submitted (29-AUG-2003) National Institutes of Health, Mammalian
            Gene Collection (MGC), Bethesda, MD 20892-2590, USA
  REMARK    NIH-MGC Project URL: http://mgc.nci.nih.gov
COMMENT     Contact: MGC help desk
            Email: cgapbs-r@mail.nih.gov
            Tissue Procurement: CLONTECH
            cDNA Library Preparation: CLONTECH Laboratories, Inc.
            cDNA Library Arrayed by: The I.M.A.G.E. Consortium (LLNL)
            DNA Sequencing by: National Institutes of Health Intramural
            Sequencing Center (NISC),
            Gaithersburg, Maryland;
            Web site: http://www.nisc.nih.gov/
            Contact: nisc_mgc@nhgri.nih.gov
            Akhter,N., Ayele,K., Beckstrom-Sternberg,S.M., Benjamin,B.,
            Blakesley,R.W., Bouffard,G.G., Breen,K., Brinkley,C., Brooks,S.,
            Dietrich,N.L., Granite,S., Guan,X., Gupta,J., Haghighi,P.,
            Hansen,N., Ho,S.-L., Karlins,E., Kwong,P., Laric,P., Legaspi,R.,
            Maduro,Q.L., Masiello,C., Maskeri,B., Mastrian,S.D.,McCloskey,J.C.,
            McDowell,J., Pearson,R., Stantripop,S., Thomas,P.J., Touchman,J.W.,
            Tsurgeon,C., Vogt,J.L., Walker,M.A., Wetherby,K.D., Wiggins,L.,
            Young,A., Zhang,L.-H. and Green,E.D.

            Clone distribution: MGC clone distribution information can be found
            through the I.M.A.G.E. Consortium/LLNL at: http://image.llnl.gov
            Series: IRAL Plate: 48 Row: f Column: 1
            This clone was selected for full length sequencing because it
            passed the following selection criteria: matched mRNA gi: 32483409.
            Method: conceptual translation.
FEATURES             Location/Qualifiers
     source          1..474
                     /organism="Homo sapiens"
                     /db_xref="taxon:9606"
                     /clone="MGC:61910 IMAGE:4767205"
                     /tissue_type="Liver"
                     /clone_lib="NIH_MGC_76"
                     /lab_host="DH10B"
                     /note="Vector: pDNR-LIB"
     Protein         1..474
                     /product="group-specific component (vitamin D binding
                     protein)"
     Region          26..206
                     /region_name="ALBUMIN"
                     /note="Albumin domain, contains five or six internal
                     disulphide bonds; albuminoid superfamily includes
                     alpha-fetoprotein which binds various cations, fatty acids
                     and bilirubin; vitamin D-binding protein which binds to
                     vitamin D, its metabolites, and fatty acids; cd00015"
                     /db_xref="CDD:28899"
     Site            order(48,52,67,88,92,95,120,123..124)
                     /site_type="binding"
                     /db_xref="CDD:28899"
     Site            order(156,159..160,163,179,182)
                     /site_type="binding"
                     /db_xref="CDD:28899"
     Region          217..392
                     /region_name="ALBUMIN"
```

```
                     and bilirubin; vitamin D-binding protein which binds to
                     vitamin D, its metabolites, and fatty acids; cl00031"
                     /db_xref="CDD:206791"
     Site            order(239,243,258,278,282,285,309..310)
                     /site_type="binding"
                     /db_xref="CDD:28899"
     Site            order(343,346..347,350,365,368)
                     /site_type="binding"
                     /db_xref="CDD:28899"
     Region          405..472
                     /region_name="VitD-bind_III"
                     /note="Vitamin D binding protein, domain III; pfam09164"
                     /db_xref="CDD:204156"
     CDS             1..474
                     /gene="GC"
                     /gene_synonym="DBP"
                     /gene_synonym="DBP/GC"
                     /gene_synonym="VDBG"
                     /gene_synonym="VDBP"
                     /coded_by="BC057228.1:63..1487"
                     /db_xref="GeneID:2638"
                     /db_xref="HGNC:4187"
                     /db_xref="MIM:139200"
```

Mascot: http://www.matrixscience.com/

Spot 19

Vitamin D binding protein

MASCOT Search Results

Protein View: gi|34785355

Group-specific component (vitamin D binding protein) [Homo sapiens]

**Database:**	NCBInr
**Score:**	140
**Expect:**	2.5e-09
**Nominal mass (M_r):**	52902
**Calculated pI:**	5.33
**Taxonomy:**	Homo sapiens

This protein sequence matches the following other entries:

- gi|158255090 from Homo sapiens
- gi|164691123 from Homo sapiens

Sequence similarity is available as an NCBI BLAST search of gi|34785355 against nr.

Search parameters

**MS data file:**	peaklist.xml
**Enzyme:**	Trypsin: cuts C-term side of KR unless next residue is P.
**Variable modifications:**	Carbamidomethyl (C), Oxidation (M)
**Mass values searched:**	39
**Mass values matched:**	19

Protein sequence coverage: 47%

Matched peptides shown in ***bold red***.

```
  1 MKRVLVLLLA VAFGHALERG RDYEKNKVCK EFSHLGKEDF TSLSLVLYSR
 51 KFPSGTFEQV SQLVKEVVSL TEACCAEGAD PDCYDTRTSA LSAKSCESNS
101 PFPVHPGTAE CCTKEGLERK LCMAALKHQP QEFPTYVEPT NDEICEAFRK
151 DPKEYANQFM WEYSTNYGQA PLSLLVSYTK SYLSMVGSCC TSASPTVCFL
201 KERLQLKHLS LLTTLSNRVC SQYAAYGEKK SRLSNLIKLA QKVPTADLED
251 VLPLAEDITN ILSKCCESAS EDCMAKELPE HTVKLCDNLS TKNSKFEDCC
301 QEKTAMDVFV CTYFMPAAQL PELPDVELPT NKDVCDPGNT KVMDKYTFEL
351 SRRTHLPEVF LSKVLEPTLK SLGECCDVED STTCFNAKGP LLKKELSSFI
401 DKGQELCADY SENTFTEYKK KLAERLKAKL PDATPTELAK LVNKRSDFAS
451 NCCSINSPPL YCDSEIDAEL KNIL
```

Unformatted sequence string: 474 residues (for pasting into other applications).

Sort peptides by ◉ Residue Number ○ Increasing Mass ○ Decreasing Mass

Show predicted peptides also

Start – End	Observed	Mr(expt)	Mr(calc)	ppm	M	Peptide
31 – 50	2328.2542	2327.2469	2327.1797	28.8	1	K.EFSHLGKEDFTSLSLVLYSR.K
38 – 50	1529.8993	1528.8920	1528.7773	75.0	0	K.EDFTSLSLVLYSR.K
51 – 65	1695.0280	1694.0207	1693.9039	68.9	1	R.KFPSGTFEQVSQLVK.E
66 – 87	2518.0486	2517.0413	2517.0094	12.7	0	K.EVVSLTEACCAEGADPDCYDTR.T + 3 Carbamidomethyl (C)
95 – 114	2265.0105	2264.0032	2263.9297	32.5	0	K.SCESNSPFPVHPGTAECCTK.E + 3 Carbamidomethyl (C)
128 – 149	2707.1956	2706.1883	2706.2020	-5.08	0	K.HQPQEFPTYVEPTNDEICEAFR.K + Carbamidomethyl (C)
128 – 150	2835.2700	2834.2627	2834.2970	-12.1	1	K.HQPQEFPTYVEPTNDEICEAFRK.D + Carbamidomethyl (C)
181 – 201	2371.0925	2370.0853	2370.0364	20.6	0	K.SYLSMVGSCCTSASPTVCFLK.E + 3 Carbamidomethyl (C); Oxidation (M)
204 – 218	1737.1493	1736.1420	1736.0308	64.1	1	R.LQLKHLSLLTTLSNR.V
208 – 218	1254.7875	1253.7802	1253.7092	56.7	0	K.HLSLLTTLSNR.V
219 – 229	1275.7202	1274.7129	1274.5601	120	0	R.VCSQYAAYGEK.K + Carbamidomethyl (C)
293 – 303	1444.7032	1443.6960	1443.5759	83.2	1	K.NSKFEDCCQEK.T + 2 Carbamidomethyl (C)
342 – 352	1388.8054	1387.7981	1387.6806	84.7	1	K.VMDKYTFELSR.R
342 – 352	1404.8064	1403.7991	1403.6755	88.1	1	K.VMDKYTFELSR.R + Oxidation (M)
346 – 352	915.4419	914.4346	914.4498	-16.6	0	K.YTFELSR.R
353 – 363	1326.8462	1325.8389	1325.7456	70.4	1	R.RTHLPEVFLSK.V
354 – 363	1170.7000	1169.6927	1169.6445	41.2	0	R.THLPEVFLSK.V
371 – 388	2092.9014	2091.8941	2091.8184	36.2	0	K.SLGECCDVEDSTTCFNAK.G + 3 Carbamidomethyl (C)
395 – 419	2974.2698	2973.2625	2973.3226	-20.2	1	K.ELSSFIDKGQELCADYSENTFTEYK.K + Carbamidomethyl (C)

No match to: 1063.3807, 1078.5677, 1307.7749, 1419.8153, 1641.9712, 1779.8910, 1804.0609, 1834.0392, 1872.0781, 1891.9780, 2186.1108, 2211.1663, 2521.0688, 2575.3713, 2711.2178, 2722.2324, 2850.2341, 2950.2576, 3102.2974, 3180.3398

```
DEFINITION  Group-specific component (vitamin D binding protein) [Homo
            sapiens].
ACCESSION   AAH57228
VERSION     AAH57228.1  GI:34785355
DBSOURCE    accession BC057228.1
KEYWORDS    MGC.
SOURCE      Homo sapiens (human)
  ORGANISM  Homo sapiens
            Eukaryota; Metazoa; Chordata; Craniata; Vertebrata; Euteleostomi;
            Mammalia; Eutheria; Euarchontoglires; Primates; Haplorrhini;
            Catarrhini; Hominidae; Homo.
REFERENCE   1  (residues 1 to 474)
  AUTHORS   Strausberg,R.L., Feingold,E.A., Grouse,L.H., Derge,J.G.,
            Klausner,R.D., Collins,F.S., Wagner,L., Shenmen,C.M., Schuler,G.D.,
            Altschul,S.F., Zeeberg,B., Buetow,K.H., Schaefer,C.F., Bhat,N.K.,
            Hopkins,R.F., Jordan,H., Moore,T., Max,S.I., Wang,J., Hsieh,F.,
            Diatchenko,L., Marusina,K., Farmer,A.A., Rubin,G.M., Hong,L.,
            Stapleton,M., Soares,M.B., Bonaldo,M.F., Casavant,T.L.,
            Scheetz,T.E., Brownstein,M.J., Usdin,T.B., Toshiyuki,S.,
            Carninci,P., Prange,C., Raha,S.S., Loquellano,N.A., Peters,G.J.,
            Abramson,R.D., Mullahy,S.J., Bosak,S.A., McEwan,P.J.,
            McKernan,K.J., Malek,J.A., Gunaratne,P.H., Richards,S.,
            Worley,K.C., Hale,S., Garcia,A.M., Gay,L.J., Hulyk,S.W.,
            Villalon,D.K., Muzny,D.M., Sodergren,E.J., Lu,X., Gibbs,R.A.,
            Fahey,J., Helton,E., Ketteman,M., Madan,A., Rodrigues,S.,
            Sanchez,A., Whiting,M., Madan,A., Young,A.C., Shevchenko,Y.,
            Bouffard,G.G., Blakesley,R.W., Touchman,J.W., Green,E.D.,
            Dickson,M.C., Rodriguez,A.C., Grimwood,J., Schmutz,J., Myers,R.M.,
            Butterfield,Y.S., Krzywinski,M.I., Skalska,U., Smailus,D.E.,
            Schnerch,A., Schein,J.E., Jones,S.J. and Marra,M.A.
  CONSRTM   Mammalian Gene Collection Program Team
  TITLE     Generation and initial analysis of more than 15,000 full-length
            human and mouse cDNA sequences
  JOURNAL   Proc. Natl. Acad. Sci. U.S.A. 99 (26), 16899-16903 (2002)
   PUBMED   12477932
REFERENCE   2  (residues 1 to 474)
  CONSRTM   NIH MGC Project
  TITLE     Direct Submission
  JOURNAL   Submitted (29-AUG-2003) National Institutes of Health, Mammalian
            Gene Collection (MGC), Bethesda, MD 20892-2590, USA
  REMARK    NIH-MGC Project URL: http://mgc.nci.nih.gov
COMMENT     Contact: MGC help desk
            Email: cgapbs-r@mail.nih.gov
            Tissue Procurement: CLONTECH
            cDNA Library Preparation: CLONTECH Laboratories, Inc.
            cDNA Library Arrayed by: The I.M.A.G.E. Consortium (LLNL)
            DNA Sequencing by: National Institutes of Health Intramural
            Sequencing Center (NISC),
            Gaithersburg, Maryland;
            Web site: http://www.nisc.nih.gov/
            Contact: nisc_mgc@nhgri.nih.gov
            Akhter,N., Ayele,K., Beckstrom-Sternberg,S.M., Benjamin,B.,
            Blakesley,R.W., Bouffard,G.G., Breen,K., Brinkley,C., Brooks,S.,
            Dietrich,N.L., Granite,S., Guan,X., Gupta,J., Haghighi,P.,
            Hansen,N., Ho,S.-L., Karlins,E., Kwong,P., Laric,P., Legaspi,R.,
            Maduro,Q.L., Masiello,C., Maskeri,B., Mastrian,S.D.,McCloskey,J.C.,
            McDowell,J., Pearson,R., Stantripop,S., Thomas,P.J., Touchman,J.W.,
            Tsurgeon,C., Vogt,J.L., Walker,M.A., Wetherby,K.D., Wiggins,L.,
            Young,A., Zhang,L.-H. and Green,E.D.

            Clone distribution: MGC clone distribution information can be found
            through the I.M.A.G.E. Consortium/LLNL at: http://image.llnl.gov
            Series: IRAL Plate: 48 Row: f Column: 1
            This clone was selected for full length sequencing because it
            passed the following selection criteria: matched mRNA gi: 32483409.
            Method: conceptual translation.
FEATURES             Location/Qualifiers
     source          1..474
                     /organism="Homo sapiens"
                     /db_xref="taxon:9606"
                     /clone="MGC:61910 IMAGE:4767205"
                     /tissue_type="Liver"
                     /clone_lib="NIH_MGC_76"
                     /lab_host="DH10B"
                     /note="Vector: pDNR-LIB"
     Protein         1..474
                     /product="group-specific component (vitamin D binding
                     protein)"
     Region          26..206
                     /region_name="ALBUMIN"
                     /note="Albumin domain, contains five or six internal
                     disulphide bonds; albuminoid superfamily includes
                     alpha-fetoprotein which binds various cations, fatty acids
                     and bilirubin; vitamin D-binding protein which binds to
                     vitamin D, its metabolites, and fatty acids; cd00015"
                     /db_xref="CDD:28899"
     Site            order(48,52,67,88,92,95,120,123..124)
                     /site_type="binding"
                     /db_xref="CDD:28899"
     Site            order(156,159..160,163,179,182)
                     /site_type="binding"
                     /db_xref="CDD:28899"
     Region          217..392
                     /region_name="ALBUMIN"
                     /note="Albumin domain, contains five or six internal
                     disulphide bonds; albuminoid superfamily includes
                     alpha-fetoprotein which binds various cations, fatty acids
```

```
     Site            order(239,243,258,278,282,285,309..310)
                     /site_type="binding"
                     /db_xref="CDD:28899"
     Site            order(343,346..347,350,365,368)
                     /site_type="binding"
                     /db_xref="CDD:28899"
     Region          405..472
                     /region_name="VitD-bind_III"
                     /note="Vitamin D binding protein, domain III; pfam09164"
                     /db_xref="CDD:204156"
     CDS             1..474
                     /gene="GC"
                     /gene_synonym="DBP"
                     /gene_synonym="DBP/GC"
                     /gene_synonym="VDBG"
                     /gene_synonym="VDBP"
                     /coded_by="BC057228.1:63..1487"
                     /db_xref="GeneID:2638"
                     /db_xref="HGNC:4187"
                     /db_xref="MIM:139200"
```

Spot 20

Fibrinogen gamma chain

MASCOT Search Results

Protein View: gi|182439

fibrinogen gamma chain [Homo sapiens]

**Database:**	NCBInr
**Score:**	108
**Expect:**	3.9e-06
**Nominal mass (M_r):**	49450
**Calculated pI:**	5.61
**Taxonomy:**	~~Homo sapiens~~

Sequence similarity is available as ~~an NCBI BLAST search of gi|182439 against nr~~.

Search parameters

**MS data file:**	peaklist.xml
**Enzyme:**	Trypsin: cuts C-term side of KR unless next residue is P.
**Variable modifications:**	~~Carbamidomethyl (C)~~, ~~Oxidation (M)~~
**Mass values searched:**	7
**Mass values matched:**	7

Protein sequence coverage: 21%

Matched peptides shown in ***bold red***.

1 MSWSLHPRNL ILYFYALLFL SSTCVAYVAT RDNCCILDER FGSYCPTTCG
51 IADFLSTYQT KVDKDLQSLE DILHQVENKT SEVKQLIK**AI** **QLTYNPDESS**
101 **KPNMIDAATL** **K**SRIMLEEIM K**YEASILTHD** **SSIRYLQEIY** **NSNNQK**IVNL
151 KEKVAQLEAQ CQEPCKDTVQ IHDITGKDCQ DIANKGAKQS GLYFIKPLKA
201 NQQFLVYCEI DGSGNGWTVF QKRLDGSVDF KKNWIQYKEG FGHLSPTGTT
251 EFWLGNEK**IH** **LISTQSAIPY** **ALRVELEDWN** **GR**TSTADYAM FK**VGPEADKY**
301 **R**LTYAYFAGG DAGDAFDGFD FGDDPSDKFF TSHNGMQFST WDNDNDKFEG
351 NCAEQDGSGW WMNKCHAGHL NGVYYQGGTY SKASTPNGYD NGIIWATWKT
401 RWYSMKKTTM KIIPFNR**LTI** **GEGQQHHLGG** **AK**QAGDV

Unformatted sequence string: ~~437 residues~~ (for pasting into other applications).

Sort peptides by ◉ Residue Number ○ Increasing Mass ○ Decreasing Mass

Show predicted peptides also

Start – End	Observed	Mr(expt)	Mr(calc)	ppm	M	Peptide
89 – 111	2536.2080	2535.2007	2535.2526	-20.5	0	K.AIQLTYNPDESSKPNMIDAATLK.S + Oxidation (M)
122 – 134	1491.7621	1490.7548	1490.7365	12.3	0	K.YEASILTHDSSIR.Y
135 – 146	1513.6602	1512.6529	1512.7208	-44.9	0	R.YLQEIYNSNNQK.I
259 – 273	1682.9773	1681.9700	1681.9515	11.0	0	K.IHLISTQSAIPYALR.V
274 – 282	1117.5176	1116.5103	1116.5200	-8.66	0	R.VELEDWNGR.T
293 – 301	1034.5195	1033.5123	1033.5192	-6.76	1	K.VGPEADKYR.L
418 – 432	1545.8191	1544.8118	1544.8059	3.80	0	R.LTIGEGQQHHLGGAK.Q

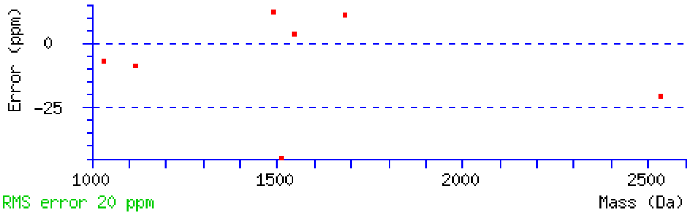


```
LOCUS       AAB59531                 437 aa            linear   PRI 16-JUN-1999
DEFINITION  fibrinogen gamma chain [Homo sapiens].
ACCESSION   AAB59531
VERSION     AAB59531.1  GI:182439
DBSOURCE    locus HUMFBRG accession M10014.1
KEYWORDS    .
SOURCE      Homo sapiens (human)
  ORGANISM  Homo sapiens
            Eukaryota; Metazoa; Chordata; Craniata; Vertebrata; Euteleostomi;
            Mammalia; Eutheria; Euarchontoglires; Primates; Haplorrhini;
            Catarrhini; Hominidae; Homo.
REFERENCE   1  (residues 1 to 437)
  AUTHORS   Rixon,M.W., Chung,D.W. and Davie,E.W.
  TITLE     Nucleotide sequence of the gene for the gamma chain of human
            fibrinogen
  JOURNAL   Biochemistry 24 (8), 2077-2086 (1985)
```

```
            alternative processing and polyadenylation reaction that occurs in
            an intervening sequence of the gamma chain.  In the mechanism, the
            alternative site is occasionally selected in place of the
            predominant site in the tenth exon, leading to termination of the
            precursor mRNA within the ninth intron, thus giving rise to the
            gamma-prime chain.  Comparisons of the amino acid sequences of the
            alpha, beta and gamma chains of fibrinogen showed that the three
            chains are related and probably evolved from a common ancestor. The
            beta and gamma chains share a significantly higher degree of
            homology with each other than with the alpha chain.  A comparison
            of the nucleotide sequences of the human and rat gamma chains shows
            that regions of high homology are localized [3].  Two tandem direct
            repeats were found at bases 1280-1309 and 1310-1340 in the 5'-end
            of the sequence, and two single-copy repeats were found in intron H
            at bases 8324-8468, and in exon 9 at bases 9367-9524. Promoter
            sequences were found at bases 1691 (TATA box) and 1724 (CAAT box)
            [4].  [3],[2] also reported the amino acid and nucleotide sequences
            of the human fibrinogen alpha-A chain and beta-B chains.  Draft
            entry and sequence on diskette kindly provided by D.W.Chung, May
            1985 [5].
            Complete source information:
            Human liver: cDNA to mRNA, library of S.L.C.Woo and T.Chandra,
            clone pHI-gamma-2 [1], clones pHI-gamma-prime-[1,2,3] [4];
            libraries of K.Matteson and D.Stafford [3]; library of D.Woods,
            clone pGF1 [2]; DNA, library of T.Maniatis [4], clones
            p-gamma-[2G7,12A9,12F3,12B8,13C8] [5].
            Method: conceptual translation.
FEATURES             Location/Qualifiers
     source          1..437
                     /organism="Homo sapiens"
                     /db_xref="taxon:9606"
                     /map="4q28"
     Protein         1..437
                     /product="fibrinogen gamma chain"
     sig_peptide     1..26
     mat_peptide     27..437
                     /product="fibrinogen gamma chain"
                     /note="G00-119-132"
     Region          29..173
                     /region_name="Fib_alpha"
                     /note="Fibrinogen alpha/beta chain family; pfam08702"
                     /db_xref="CDD:149682"
     Region          174..415
                     /region_name="FReD"
                     /note="Fibrinogen-related domains (FReDs); C terminal
                     globular domain of fibrinogen. Fibrinogen is involved in
                     blood clotting, being activated by thrombin to assemble
                     into fibrin clots. The N-termini of 2 times 3 chains come
                     together to form a globular...; cd00087"
                     /db_xref="CDD:28971"
     Site            301
                     /site_type="other"
                     /note="gamma-gamma dimer interface [polypeptide binding]"
                     /db_xref="CDD:28971"
     Site            order(344,346,348)
                     /site_type="other"
                     /note="Ca2+ binding site [ion binding]"
                     /db_xref="CDD:28971"
     Site            order(352,355..356,365..366)
                     /site_type="other"
                     /note="polymerization pocket"
                     /db_xref="CDD:28971"
     CDS             1..437
                     /gene="FGG"
                     /coded_by="join(M10014.1:1799..1876,M10014.1:1973..2017,
                     M10014.1:2207..2390,M10014.1:2510..2603,
                     M10014.1:4211..4341,M10014.1:4645..4778,
                     M10014.1:5758..5942,M10014.1:7426..7703,
                     M10014.1:9342..9511,M10014.1:10054..10068)"
```

Spot 21

Fibrinogen gamma chain

MASCOT Search Results

Protein View: gi|182439

fibrinogen gamma chain [Homo sapiens]

**Database:**	NCBInr
**Score:**	134
**Expect:**	9.8e-09
**Nominal mass (M_r):**	49450
**Calculated pI:**	5.61
**Taxonomy:**	Homo sapiens

Sequence similarity is available as an NCBI BLAST search of gi|182439 against nr.

Search parameters

**MS data file:**	peaklist.xml
**Enzyme:**	Trypsin: cuts C-term side of KR unless next residue is P.
**Variable modifications:**	Carbamidomethyl (C), Oxidation (M)
**Mass values searched:**	18
**Mass values matched:**	11

Protein sequence coverage: 36%

Matched peptides shown in ***bold red***.

```
  1 MSWSLHPRNL ILYFYALLFL SSTCVAYVAT RDNCCILDER FGSYCPTTCG
 51 IADFLSTYQT KVDKDLQSLE DILHQVENKT SEVKQLIKAI QLTYNPDESS
101 KPNMIDAATL KSRIMLEEIM KYEASILTHD SSIRYLQEIY NSNNQKIVNL
151 KEKVAQLEAQ CQEPCKDTVQ IHDITGKDCQ DIANKGAKQS GLYFIKPLKA
201 NQQFLVYCEI DGSGNGWTVF QKRLDGSVDF KKNWIQYKEG FGHLSPTGTT
251 EFWLGNEKIH LISTQSAIPY ALRVELEDWN GRTSTADYAM FKVGPEADKY
301 RLTYAYFAGG DAGDAFDGFD FGDDPSDKFF TSHNGMQFST WDNDNDKFEG
351 NCAEQDGSGW WMNKCHAGHL NGVYYQGGTY SKASTPNGYD NGIIWATWKT
401 RWYSMKKTTM KIIPFNRLTI GEGQQHHLGG AKQAGDV
```

Unformatted sequence string: 437 residues (for pasting into other applications).

Sort peptides by ◉ Residue Number ○ Increasing Mass ○ Decreasing Mass

Show predicted peptides also

Start – End	Observed	Mr(expt)	Mr(calc)	ppm	M	Peptide
32 – 40	1194.5762	1193.5689	1193.4805	74.1	0	R.DNCCILDER.F + 2 Carbamidomethyl (C)
89 – 111	2536.3257	2535.3184	2535.2526	25.9	0	K.AIQLTYNPDESSKPNMIDAATLK.S + Oxidation (M)
122 – 134	1491.8589	1490.8516	1490.7365	77.2	0	K.YEASILTHDSSIR.Y
135 – 146	1513.8315	1512.8243	1512.7208	68.4	0	R.YLQEIYNSNNQK.I
154 – 177	2768.3655	2767.3582	2767.3269	11.3	1	K.VAQLEAQCQEPCKDTVQIHDITGK.D + 2 Carbamidomethyl (C)
189 – 199	1293.8672	1292.8599	1292.7492	85.6	0	K.QSGLYFIKPLK.A
259 – 273	1683.0481	1682.0408	1681.9515	53.1	0	K.IHLISTQSAIPYALR.V
274 – 282	1117.6117	1116.6044	1116.5200	75.6	0	R.VELEDWNGR.T
293 – 301	1034.6045	1033.5972	1033.5192	75.4	1	K.VGPEADKYR.L
348 – 364	1958.9633	1957.9560	1957.7723	93.8	0	K.FEGNCAEQDGSGWWMNK.C
365 – 382	2011.9912	2010.9839	2010.9007	41.4	0	K.CHAGHLNGVYYQGGTYSK.A + Carbamidomethyl (C)

No match to: 980.5553, 1012.5537, 1121.5870, 1149.6017, 1164.7363, 2211.1465, 2239.0947

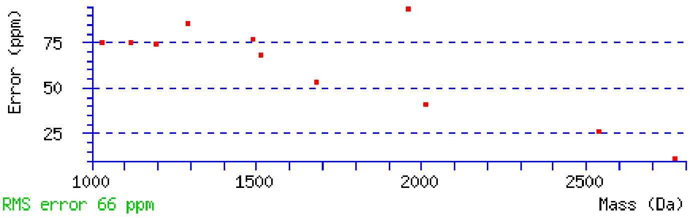


```
LOCUS       AAB59531                 437 aa            linear   PRI 16-JUN-1999
DEFINITION  fibrinogen gamma chain [Homo sapiens].
ACCESSION   AAB59531
VERSION     AAB59531.1  GI:182439
DBSOURCE    locus HUMFBRG accession M10014.1
KEYWORDS    .
SOURCE      Homo sapiens (human)
  ORGANISM  Homo sapiens
            Eukaryota; Metazoa; Chordata; Craniata; Vertebrata; Euteleostomi;
```

```
  AUTHORS   Rixon,M.W., Chung,D.W. and Davie,E.W.
  TITLE     Nucleotide sequence of the gene for the gamma chain of human
            fibrinogen
  JOURNAL   Biochemistry 24 (8), 2077-2086 (1985)
   PUBMED   2990550
COMMENT     The gamma-prime chain in human fibrinogen results from an
            alternative processing and polyadenylation reaction that occurs in
            an intervening sequence of the gamma chain.  In the mechanism, the
            alternative site is occasionally selected in place of the
            predominant site in the tenth exon, leading to termination of the
            precursor mRNA within the ninth intron, thus giving rise to the
            gamma-prime chain.  Comparisons of the amino acid sequences of the
            alpha, beta and gamma chains of fibrinogen showed that the three
            chains are related and probably evolved from a common ancestor. The
            beta and gamma chains share a significantly higher degree of
            homology with each other than with the alpha chain.  A comparison
            of the nucleotide sequences of the human and rat gamma chains shows
            that regions of high homology are localized [3].  Two tandem direct
            repeats were found at bases 1280-1309 and 1310-1340 in the 5'-end
            of the sequence, and two single-copy repeats were found in intron H
            at bases 8324-8468, and in exon 9 at bases 9367-9524. Promoter
            sequences were found at bases 1691 (TATA box) and 1724 (CAAT box)
            [4].  [3],[2] also reported the amino acid and nucleotide sequences
            of the human fibrinogen alpha-A chain and beta-B chains.  Draft
            entry and sequence on diskette kindly provided by D.W.Chung, May
            1985 [5].
            Complete source information:
            Human liver: cDNA to mRNA, library of S.L.C.Woo and T.Chandra,
            clone pHI-gamma-2 [1], clones pHI-gamma-prime-[1,2,3] [4];
            libraries of K.Matteson and D.Stafford [3]; library of D.Woods,
            clone pGF1 [2]; DNA, library of T.Maniatis [4], clones
            p-gamma-[2G7,12A9,12F3,12B8,13C8] [5].
            Method: conceptual translation.
FEATURES             Location/Qualifiers
     source          1..437
                     /organism="Homo sapiens"
                     /db_xref="taxon:9606"
                     /map="4q28"
     Protein         1..437
                     /product="fibrinogen gamma chain"
     sig_peptide     1..26
     mat_peptide     27..437
                     /product="fibrinogen gamma chain"
                     /note="G00-119-132"
     Region          29..173
                     /region_name="Fib_alpha"
                     /note="Fibrinogen alpha/beta chain family; pfam08702"
                     /db_xref="CDD:149682"
     Region          174..415
                     /region_name="FReD"
                     /note="Fibrinogen-related domains (FReDs); C terminal
                     globular domain of fibrinogen. Fibrinogen is involved in
                     blood clotting, being activated by thrombin to assemble
                     into fibrin clots. The N-termini of 2 times 3 chains come
                     together to form a globular...; cd00087"
                     /db_xref="CDD:28971"
     Site            301
                     /site_type="other"
                     /note="gamma-gamma dimer interface [polypeptide binding]"
                     /db_xref="CDD:28971"
     Site            order(344,346,348)
                     /site_type="other"
                     /note="Ca2+ binding site [ion binding]"
                     /db_xref="CDD:28971"
     Site            order(352,355..356,365..366)
                     /site_type="other"
                     /note="polymerization pocket"
                     /db_xref="CDD:28971"
     CDS             1..437
                     /gene="FGG"
                     /coded_by="join(M10014.1:1799..1876,M10014.1:1973..2017,
                     M10014.1:2207..2390,M10014.1:2510..2603,
                     M10014.1:4211..4341,M10014.1:4645..4778,
                     M10014.1:5758..5942,M10014.1:7426..7703,
                     M10014.1:9342..9511,M10014.1:10054..10068)"
```
